# Intrinsic mechanical properties of two-dimensional covalent organic frameworks†

**DOI:** 10.1039/d5sc02180d

**Published:** 2025-07-21

**Authors:** Liangtao Xiong, Chengbin Fu, Jiaxin Tian, Yubo Geng, Lixin Han, Han Zhang, Haoyuan Li

**Affiliations:** a School of Microelectronics, Shanghai University Shanghai 201800 China lihaoyuan@shu.edu.cn; b Department of Chemistry, College of Sciences, Shanghai University Shanghai 200444 China; c Key Laboratory of Advanced Display and System Applications, Ministry of Education, Shanghai University Shanghai 200072 China

## Abstract

Understanding the mechanical properties of two-dimensional covalent organic frameworks (2D COFs) is critical to their design for flexible devices, energy storage and catalysis applications. To date, only a limited number of 2D COFs have been examined, leaving the material design principles largely undefined. Furthermore, the measured results are often complicated by various extrinsic factors, causing difficulties in deciphering the underlying relationships. Here, we establish rules governing the intrinsic mechanical properties of 2D COFs based on molecular simulations of 86 structures under uniaxial tensile stress. Interestingly, we found that the mechanical properties of these nanoscale structures can be comprehended through principles traditionally applied to macroscopic objects. This enables quantitatively predicting the mechanical properties of 2D COFs based on their chemical linkage, topology, and pore dimensions, thereby facilitating material design. Counterintuitively, integrating rigid molecular groups into a 2D framework can potentially compromise overall mechanical strength by inducing imbalanced local strain. These findings pave the way for designing robust 2D COFs for diverse applications and serve as a solid foundation for fully unraveling the roles of various extrinsic factors in the future.

## Abstract 2

We show that macroscopic principles can predict the nanoscale behavior of 2D covalent organic frameworks and that rigid molecular groups may weaken their overall strength by inducing local strain imbalance.

## Graphical abstract

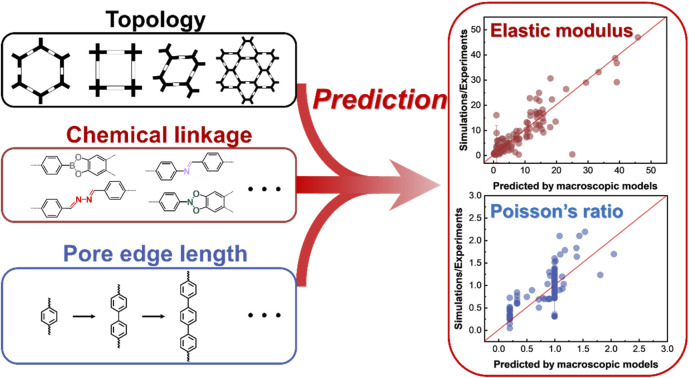

## 1. Introduction

Two-dimensional covalent organic frameworks (2D COFs) are porous crystalline organic polymers with diverse structures.^1–6^ A fundamental property regarding their applications is their response to tensile stress.^7–23^ For instance, when 2D COFs are used as artificial solid electrolyte interfaces in lithium batteries, they must exhibit high Young's moduli and fracture strength to inhibit the growth of lithium dendrites while maintaining film integrity.^15–19^ Conversely, flexible devices necessitate moderate Young's moduli and high fracture strains.^7,8^ For catalytic applications, it has been shown that 2D COFs with a higher fracture strain are beneficial for a higher reaction rate.^23^ Thus, a thorough understanding of the mechanical properties of 2D COFs is pivotal for guiding their molecular design. However, this endeavor is complicated by challenges in accurately measuring these properties and the incomplete identification of influencing factors. Investigating the intrinsic mechanical properties of 2D COFs is hindered by challenges in preparing large defect-free films and the limited measurement techniques available.^24–29^ Notably, a 90 nm-thick TAPB-PDA COF film has been stretched using a robotic arm on a water surface, resulting in a Young's modulus of 1.45 GPa.^30^ However, this method is unsuitable for thinner films prone to defects and cracks. To elucidate the mechanical properties of 2D COF films, atomic force microscopy has been employed to obtain force–separation curves in nanoindentation experiments, which can be fitted to appropriate theoretical models (*e.g.*, Schwering-type solution^31^ and Hertz model^32^) to calculate Young's moduli.^33–35^ This technique has been applied to various 2D COFs, including TTA-DHTA COF,^33^ TAPB-PDA COF,^15,34,36^ DMTP-PDA COF,^36^ 2DCOF-1,^35^ TAPB-BTCA-MCOF,^34^ and PPDA-BTCA-MCOF.^34^ To assess the deformation and fracture behaviors of 2D COFs, *in situ* scanning electron microscopy has been used on a 17 nm-wide TAPB-DHTA COF film with a supporting cantilever, which bends and introduces a tensile load. This experiment reported a Young's modulus of 10.38 GPa and a fracture strength of 0.75 GPa.^37^ Owing to the difficulty in measuring the mechanical properties of 2D COFs, data available are sparse and insufficient to elucidate molecular design principles. Moreover, discrepancies are commonly observed in reported Young's modulus values for identical 2D COFs, likely owing to variations in the sample quality and approximations introduced by different mathematical models used to interpret experimental data.^15,30,34,36^

Molecular simulations have emerged as a valuable tool for understanding the mechanical properties of 2D COFs.^13,38–43^ Among these techniques, density-functional theory (DFT) calculations stand out for their accuracy but are limited to relatively smaller systems, typically accommodating a few hundred atoms, which restricts the size of 2D COFs they can deal with.^38,43^ Classical force fields, on the other hand, allow for the simulation of much larger systems, extending to scales of tens of nanometers. For example, the adaptive intermolecular reactive empirical bond order (AIREBO) and Tersoff classical force fields were used in the study of DTPA-COF, where a phase transition was identified.^39^ Moreover, the AIREBO potential has been used to investigate Young's moduli and Poisson's ratios of multilayered structures of COF-1, COF-5, and TP-COF.^13^ Additionally, we have used all-atom optimized potentials for liquid simulations (OPLS-AA) force field^44^ to study the Young's moduli and Poisson's ratios of COF-5 and TAPB-PDA COF sheets.^41^ However, classical force fields cannot describe bond breaking, limiting their applicability to understanding mechanical properties under high strain or during fracture. To address this, reactive force field (ReaxFF) has been applied to simulate the fracturing behavior of a monolayer of COF-1 measuring 7.7 × 9.3 nm^2^; this approach revealed stress–strain curves with two distinct linear regions corresponding to Young's moduli of 20 GPa and 160 GPa, an ultimate strength of 27.9 GPa, and an ultimate strain of 37%.^40^ However, the application of ReaxFF is restricted owing to the available parameter sets, which are not universally applicable to all 2D COFs. Recently, machine-learning (ML) potentials have been used to study the elastic constants of COF-1 and COF-5 at various temperatures.^42^ Despite their promise, trained ML potentials are generally restricted to a limited number of molecular systems at this stage, thereby constraining their broader applicability. Therefore, only a few 2D COFs have been theoretically evaluated for their mechanical properties so far, primarily those with hexagonal topologies. The diverse chemical structures and topologies of 2D COFs—including hexagonal, tetragonal, rhombic, and star-pore configurations—pose challenges in elucidating their mechanical properties for targeted material design.

Another obstacle in understanding the mechanical properties of 2D COFs is that they are influenced by various imperfect factors during sample preparation and experimental measurement, which makes the obtained results likely a combined outcome of both intrinsic and extrinsic factors and deriving universal rules from the already limited data becomes even more difficult. It has been shown that the mechanical properties of 2D COFs may depend on the number of layers, pore size, density, defects, and temperature.^13,15,34–37,41,42,45,46^ So far, our understanding of the detailed roles of these extrinsic factors remains limited, with some studies showing controversial results. While an increase in the number of layers was believed to lead to higher Young's modulus in one study,^15^ TAPB-PDA COF sheets were shown to have layer sliding in nanoindentation tests by AFM and resulted in a decrease in Young's modulus.^36^ On the other hand, both nanoindentation experiments^34^ and molecule dynamic (MD) simulations with the AIREBO potential^13^ indicated that Young's modulus of 2D COFs decreases with increasing pore size (or decreasing density), which were thought to be related to interlayer interactions or the softer, connected benzene rings. Structural defects can be detrimental to the mechanical properties of 2D COFs and may alter the shape of the stress–strain curve, as suggested by nanoindentation tests, transmission electron microscope experiments, and MD simulations.^41,45,46^ However, *in situ* scanning electron microscope measurement showed that the fracture strength of COF_TAPB–DHTA_ films with cracks was the same as that of crack-free films,^37^ and nanoindentation experiments revealed that the force–displacement curve of the fractured region in single-crystal 2D COF-1 films remained unchanged.^35^ Finally, molecular simulations suggested that high temperatures may lead to a reduction in mechanical properties; when the temperature of COF-1 and COF-5 increased from ∼40–60 K to 500 K, their Young's moduli decreased from 6.6 GPa and 7.2 GPa to 2.9 GPa and 3.8 GPa, respectively (decreased by 56% and 47% respectively); this was understood by the thermal rippling (out-of-plane structural deformation) at high temperatures.^42^

**Fig. 1:**
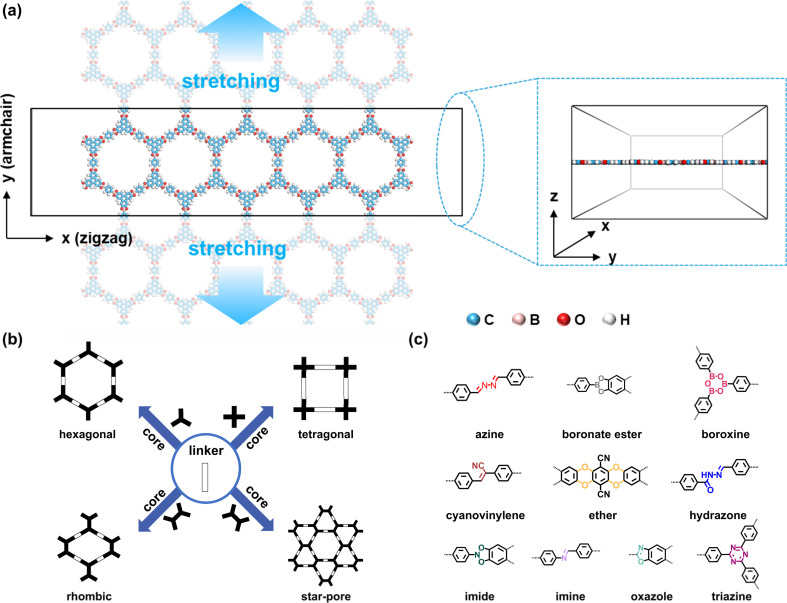
(a) Illustration of a 2D COF under uniaxial tensile stress using the DFTB method. (b) Hexagonal, tetragonal, rhombic, and star-pore topologies. (c) Chemical linkages investigated herein.

In this study, we aim to establish the rules governing the intrinsic mechanical properties of 2D COFs (in the absence of film imperfection and nonuniform forces across layers that cause layer sliding), which is a necessary step in ultimately unraveling their complete mechanical properties (under effects of defects, grain boundaries, and possible layer sliding). The various extrinsic factors (imperfection in the 2D COF films and sliding from possible nonuniform forces across layers) mostly negatively impact the mechanical properties. Therefore, such rules can provide the theoretical limit of the mechanical properties for materials design. To achieve this goal, we systematically investigate the mechanical properties of 86 2D COFs under uniaxial tensile stress (Fig. 1a), encompassing hexagonal, tetragonal, rhombic, star-pore topologies (Fig. 1b), and chemical linkages such as azine,^47^ boronate ester,^1^ boroxine,^1^ cyanovinylene,^48^ ether,^49^ hydrazone,^50^ imide,^51^ imine,^52^ oxazole,^53,54^ and triazine^55^ (Fig. 1c). Detailed chemical structures and lattice parameters of the studied 2D COFs are summarized in Tables S1, S2 and Fig. S13 of ESI.† To effectively obtain their mechanical properties, we rely on a recently developed self-consistent charge density-functional tight-binding (SCC-DFTB) method, achieving accuracies comparable to DFT at reduced computational cost and offering a broader range of applications (can describe high strains and have ready-to-use parameter sets) compared to classical and reactive force fields.^56–58^ This method has been used in the study of 2D COFs,^59–62^ including the calculation of the bulk modulus of a single-layer CDB-TBPor-COF^59^ and the force constants of a series of honeycomb 2D COFs.^61^ Our benchmarks showed that the energy per layer does not change for monolayer and few-layer sheets (the dihedrals may differ, see Fig. S7–S11†), similar to the result in previous molecular modeling.^36^ Therefore, we choose to model monolayer to allow us to focus on modeling extended sizes, suppressing the finite site effect (see Fig. S6† for a benchmark on the model size). The calculated Young's moduli and Poisson's ratios of the modeled 2D COFs are summarized in Table S3.† Overall, the values obtained through DFTB agree well with previously calculated results (Section 4 of ESI†). Our findings reveal that the intrinsic elastic moduli of 2D COFs retain the characteristics of their corresponding macroscopic networks, with proportional coefficients determined on the basis of the type of chemical linkage. This quantitative relationship offers a predictive tool for estimating the elastic moduli of 2D COFs prior to their synthesis. Additionally, the calculations show that the stress–strain curves exhibit multiple linear or fluctuating stages under high strain, depending on the type of molecular deformations occurring during stretching. Fracture characteristics, interestingly, are often not correlated with the weakest bonds. These results establish general design principles for manipulating the mechanical properties of 2D COFs, thereby guiding the targeted molecular and topological design of these materials for various applications.

## 2. Results and discussions

### 2.1 Similarities between 2D COFs and macroscopic networks

**Fig. 2:**
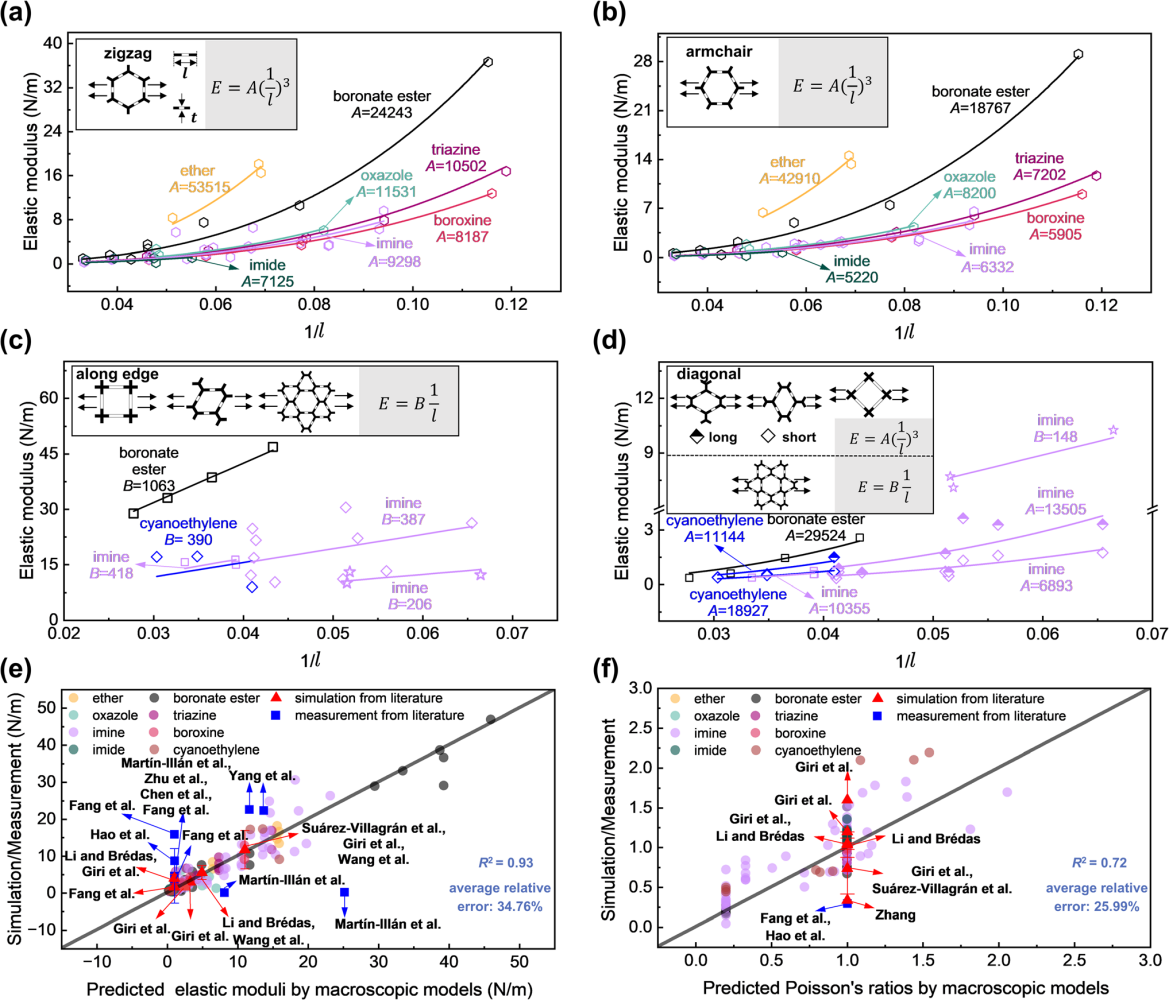
Relationships between the elastic modulus and the inverse of pore edge length (*l*) for hexagonal 2D COFs when stretched in the (a) zigzag and (b) armchair directions. Relationships between the elastic modulus and the inverse of pore edge length for tetragonal, rhombic, and star-pore 2D COFs when stretched (c) along the pore edge and (d) diagonal directions. The insets illustrate the stretching direction, quantitative relationships, and the definitions of the pore edge length and width (*t*). Comparative analysis of the predicted (e) elastic moduli and (f) Poisson's ratios of 2D COFs obtained using macroscopic models listed in Table 1*versus* those obtained through simulation/measurement (Tables S5 and S6 in Section 8 of the ESI†). Our calculations are performed at ≤5% strain. The enlarged versions of (a–f) are shown in Fig. S43–S48 of the ESI.†

We found that under low strain (less than approximately 5%), the mechanical properties of 2D COFs with identical chemical linkages exhibit characteristics akin to those observed in their corresponding macroscopic networks (Fig. 2a–d and Table 1). Specifically, the elastic moduli (*E*) of these 2D COFs are proportional to either 1/*l*·*E*_s_*t* or (1/*l*)^3^·*E*_s_*t*^3^. Here, *E*_s_ denotes Young's modulus of the bulk material constituting the macroscopic network and can be analogously considered as the effective modulus of a 3D dense material; *t* and *l* denote the width and length of the pore edge (Fig. 2a). We note that, for multilayer 2D COF sheets, a factor of *n* (denoting the number of layers) enters the above expressions (see Table 1), which is the upper limit of the elastic moduli in the absence of layer sliding. Based on this quantitative relationship, Young's modulus decreases with the pore size. This trend is consistent with the previous nanoindentation experiments experiment by AFM^34^ and MD simulations with (AIREBO) potential,^13^ in which the reasons were speculated to be the weakening of interlayer interactions or the increase in the strain of benzene ring structures. Here, we show that this fundamental quantitative relationship is indeed the origin of previous observations.

**Table 1:** Elastic moduli and Poisson's ratios of hexagonal, tetragonal, rhombic, and star-pore macroscopic networks

Topology	Direction	Elastic modulus	Poisson's ratio
Hexagonal^63,64^	Zigzag	3^−1/2^·4(1/*l*)^3^·*E*_s_*t*^3^·*n*	1
	Armchair	3^−1/2^·4(1/*l*)^3^·*E*_s_*t*^3^·*n*	1
Tetragonal^63,65,66^	Along edge	1/*l*·*E*_s_*t*·*n*	0.2
	Diagonal	2(1/*l*)^3^·*E*_s_*t*^3^·*n*	1
Rhombic^66,67^^a^	Along edge	1/*l*·*E*_s_*t*·*n*	0.2
	Short diagonal	(cos(*θ*/2)/sin(*θ*/2)^3^)·(1/*l*)^3^·*E*_s_*t*^3^·*n*	cot(*θ*/2)^2^
	Long diagonal	(cos(*θ*/2)/sin(*θ*/2)^3^)·(1/*l*)^3^·*E*_s_*t*^3^·*n*	cot(*θ*/2)^2^
Star-pore^63^	Along edge	3^−1/2^(1/*l*)·*E*_s_*t*·*n*	0.33
	Diagonal	3^−1/2^(1/*l*)·*E*_s_*t*·*n*	0.33

a Further information on the elastic moduli of rhombic networks in the diagonal direction can be found in Table S4, Fig. S16, and eqn (S1)–(S7) in Sections 6 and 7 of the ESI. *θ* denotes the internal angle pointing to the stretching direction. *n* denotes the number of layers.

Table 2 lists the *E*_s_*t*^3^ and *E*_s_*t* values, which decrease in the order of ether, boronate ester, oxazole, triazine, imine, boroxine, and imide linkages. We note that, while chemical bonds approximately follow harmonic potentials around their equilibrium positions, the manifestation of mechanical properties of these nanostructures akin to those at the macroscopic scale, with coefficients determined by the type of chemical linkages, is unexpected. Further analysis indicated that the type of chemical linkage considerably influences the structural deformation characteristics of the 2D COFs under low strain (Fig. S17 and S18†). In particular, chemical linkages with large *E*_s_*t*^3^ values are associated with deformations in bond lengths and angles, indicating higher rigidity. Conversely, those with small *E*_s_*t*^3^ values correspond to more dihedral angle changes, thereby exhibiting softer structural behavior.

**Table 2:** *E*_s_ *t* ^3^ and *E*_s_*t* values for different chemical linkages^a^^,^^b^

Coefficient	*E*_s_ *t* (10^−10^ N)	*E*_s_ *t* ^3^ (10^−30^ N m^2^)
Ether	—	20 877
Boronate ester	1063	11 129
Oxazole	—	4272
Triazine	—	3833
Imine	355	4339
Boroxine	—	3051
Imide	—	2673
Cyanoethylene	390	6561

a The values are averaged over different topologies and directions. The detailed *E*_s_*t*^*x*^ values are listed in Table S7.

b Azine and hydrazone chemical linkages have too few data points for fitting functions and thus are not included in the table.

Similar to the case of elastic moduli, Poisson's ratios of 2D COFs preserve the characteristics of their corresponding macroscopic networks. Specifically, the hexagonal 2D COFs exhibit isotropic characteristics in their Poisson's ratios, ranging from 0.6–1.5 (Fig. S19†); these ratios align closely with the Poisson's ratio of the hexagonal network, which is 1.^64^ By contrast, the tetragonal and rhombic 2D COFs exhibit directional dependence. Their Poisson's ratios along the edge direction are 0.16–0.44 and approximately 0.25, respectively (Fig. S20 and S21†), both approximating their macroscopic network counterpart of 0.2.^66^ The Poisson's ratio of the tetragonal 2D COFs in the diagonal direction is approximately 1.05, resembling the value of their corresponding macroscopic network, which is 1.^65^ The Poisson's ratios of the rhombic 2D COFs range from 0.50 to 1.54 along the short diagonal direction and from 0.89 to 2.19 along the long diagonal direction; these ratios are close to that of the corresponding macroscopic networks of cot(*θ*/2)^2^ (Fig. S21†).^67^ Notably, the star-pore 2D COFs deviate from this trend, and their Poisson's ratios are in the range of 0.59–0.91, exceeding the value of 0.33 observed for their corresponding macroscopic networks (Fig. S22†).^63^ This may be attributed to deviations in the orientations of their topological edges from ideal star-pore networks (Fig. S23†), which resulted in increased angular deformations and consequently greater contraction.

The aforementioned quantitative relationships help predict the elastic moduli and Poisson's ratios of 2D COFs with different pore sizes and chemical linkages prior to their synthesis (Fig. 2e and f). This capability plays a key role in the rational design of 2D COFs tailored to meet specific mechanical requirements. To demonstrate the applicability of these quantitative relationships, we have compiled literature data on the elastic moduli and Poisson's ratios of 2D COFs from both simulations^13,37,39–42^ and measurements.^15,30,33–37^ Overall, simulation results from other studies are consistent with our derived quantitative relationships. However, deviations are observed in the reported experimental values; this is expected since extrinsic factors such as defects^41,45^ may be present that comprise the intrinsic mechanical properties, as discussed above.^15,30,34,36^

### 2.2 Mechanical properties under high strain

**Fig. 3:**
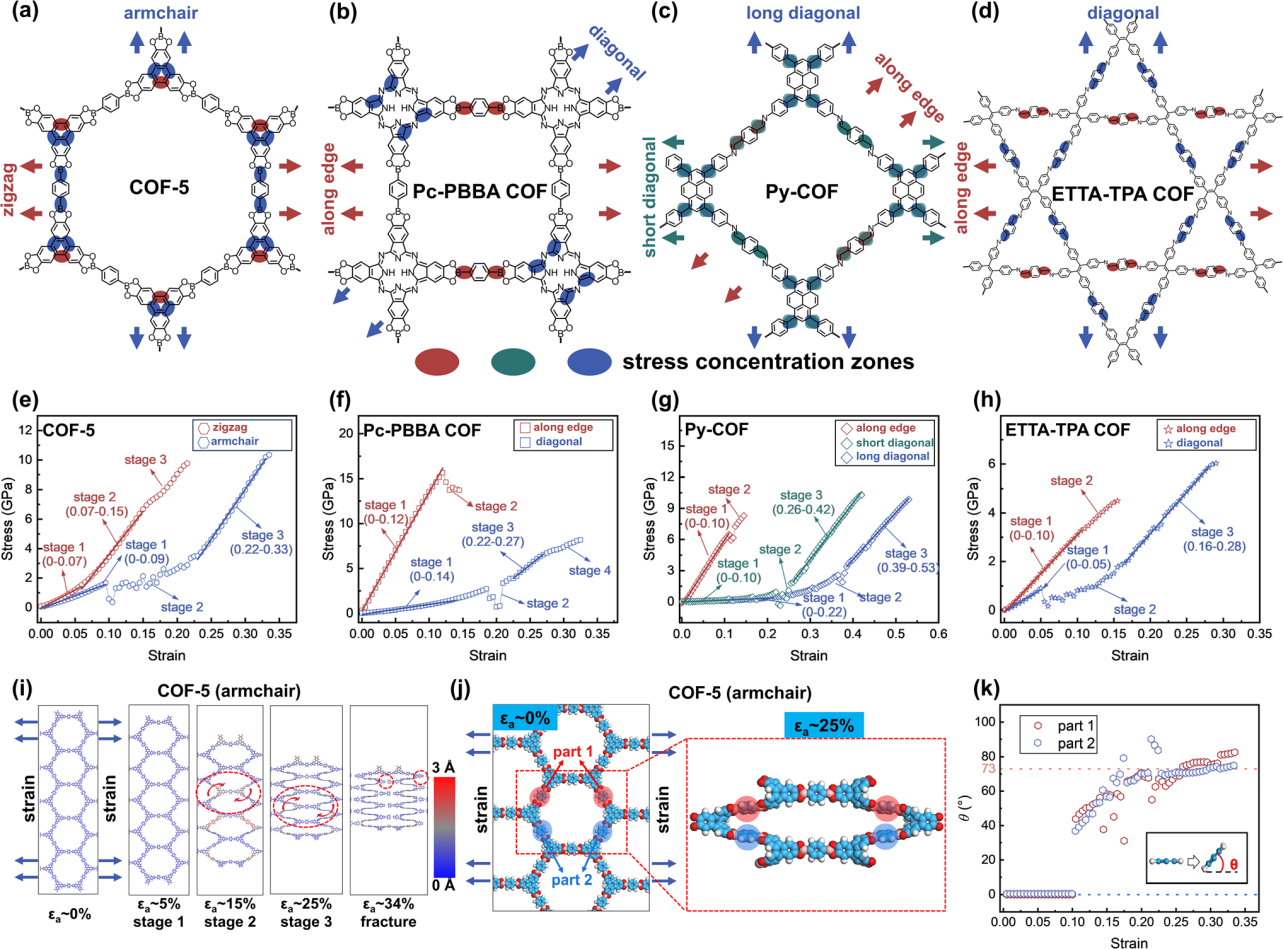
Chemical structures of (a) COF-5, (b) Pc-PBBA COF, (c) Py-COF, and (d) ETTA-TPA COF. Arrows indicate the stretching directions. Highlighted regions denote stress concentration zones during stretching. Stress–strain curves of (e) hexagonal COF-5, (f) tetragonal Pc-PBBA COF, (g) rhombic Py-COF, and (h) star-pore ETTA-TPA COF. (i) Structural changes under strain when COF-5 is stretched in the armchair direction. The dashed circles denote regions exhibiting twisting or breakage. The color bar indicates the degree of out-of-plane deformation. (j) Twisting of benzene rings (parts 1 and 2) in the PBBA unit of COF-5. (k) Evolution of the torsional angles of these benzene rings in the PBBA unit relative to the 2D plane during stretching along the armchair direction. The inset illustrates the torsional angles between the benzene ring and the 2D plane.

To elucidate the mechanical properties under high strain, four representative 2D COFs are further studied, including hexagonal COF-5,^1^ tetragonal Pc-PBBA COF,^68^ rhombic Py-COF,^69^ and star-pore ETTA-TPA COF^70^ (Fig. 3a–d). Their stress–strain curves exhibit multiple distinct stages characterized by either linear or fluctuating behavior (Fig. 3e–h). For example, when COF-5 is subjected to tensile stress along the zigzag direction, the stress–strain curve displays an initial linear region (Fig. 3e). At a strain value of approximately 7%, it transitions into a second linear stage with an increase in slope. As the strain continues to increase to 15%, the material enters a nonlinear phase characterized by a reduced slope. The material exhibits an ultimate strength and fracture strength of 9.8 GPa at a strain of 21%. In contrast, when COF-5 is stretched along the armchair direction, the stress–strain response exhibits three distinct stages prior to fracture: an initial linear stage, followed by a fluctuation stage, and finally a high-slope linear stage. In this direction, the ultimate and fracture strain occurs at a strain of 33.5%, with both ultimate strength and fracture strength measured at 10.3 GPa. These values are higher than those observed in the zigzag direction.

We note that 2D COF films have been found to fracture at small strains experimentally.^37,46^ For example, *in situ* scanning electron microscopy measurement showed that TAPB-DHTA COF films fracture strain of 3–13%, in contrast to the same authors' MD (ReaxFF) calculation suggesting fracture strain of 27–35%.^37^ The smaller experimental fracture strain can be attributed to the presence of defects,^41^ highlighting the need to further improve their material qualities.

For the tetragonal Pc-PBBA COF, rhombic Py-COF, and star-pore ETTA-TPA COF, tensile stress applied along the edge reveals only one or two stages, characterized by significantly steeper stress–strain slopes and reduced fracture strains of 14.5%, 14.5%, and 15.5%, respectively. Specifically, the Pc-PBBA COF exhibits an ultimate strength of 15.6 GPa at a strain of 12% and a fracture strength of 13.7 GPa at 14.5%. For both the Py-COF and ETTA-TPA COF, the ultimate and fracture strengths coincide at the same strain levels, measuring 8.3 GPa and 4.5 GPa, respectively. Consequently, these three 2D COFs display high tensile strength but low fracture toughness when strained along the edge, indicating a propensity for brittle failure compared to COF-5. On the other hand, tensile deformation in the diagonal direction involves multiple deformation stages and substantially higher fracture strains—32.5% for Pc-PBBA COF, 53% and 42% for the short and long diagonals of Py-COF, and 29% for ETTA-TPA COF. The corresponding fracture strength values are 8.2 GPa for Pc-PBBA COF, 10.3 GPa (short diagonal), and 9.9 GPa (long diagonal) for Py-COF, and 6.0 GPa for ETTA-TPA COF, respectively. This indicates that these COFs can withstand higher strain without fracturing when stretched diagonally, highlighting their enhanced mechanical resilience in this direction. The stress–strain characteristics of 2D COFs are intrinsically linked to the deformation patterns of their pores and molecular groups under tensile force. For COF-5, when stretched in the zigzag direction, the pore shape undergoes a monotonic evolution while maintaining a planar configuration, with a minor contraction observed in the perpendicular direction (Fig. S28†). Similar behavior is noted for Pc-PBBA COF, Py-COF, and ETTA-TPA COF when stretched along their edges. In these instances, the strain is primarily attributed to bond elongation, resulting in high slopes on the stress–strain curves, culminating in fracture upon bond failure (Fig. S29–S31†). When COF-5 is stretched in the armchair direction, or when Pc-PBBA COF, Py-COF, and ETTA-TPA COF are stretched diagonally, these materials exhibit considerable sheet contraction perpendicular to the stretching axis, coupled with notable out-of-plane deformations (Fig. 3i and S29–S31†). The applied stress induces twisting of bond and dihedral angles, leading to small initial slopes on the stress–strain curves; these out-of-plane deformation fluctuations contribute to nonlinear stress–strain responses. As angle deformations reach their limits, bond stretching becomes predominant, evidenced by increased slopes on the stress–strain curves. Specifically, for COF-5 stretched in the armchair direction, the benzene rings in the 1,4-phenylene-bis(PBBA) unit exhibit out-of-plane twisting starting around 10% strain (Fig. 3j and k), causing fluctuations in the stress–strain curve. This twisting phase achieves a maximum angle of approximately 73° at 22% strain (we note that layer stacking may suppress the degree of dihedral angle change, see Fig. S10†), beyond which further strain primarily extends the bonds. This transition marks the onset of a third stage in the stress–strain curve, characterized by a steep slope. The predominance of angular adjustments during stress application leads to higher deformation before fracture, which applies to the diagonal stretching of Pc-PBBA COF, Py-COF, and ETTA-TPA COF as well.

Further analysis reveals that Poisson's ratios exhibit substantial alterations at elevated strain levels, characterized by intricate patterns associated with variations in pore dimensions. Detailed information is provided in Fig. S32 and S33 in Section 14 of the ESI.†

### 2.3 Fracturing of 2D COFs

**Fig. 4:**
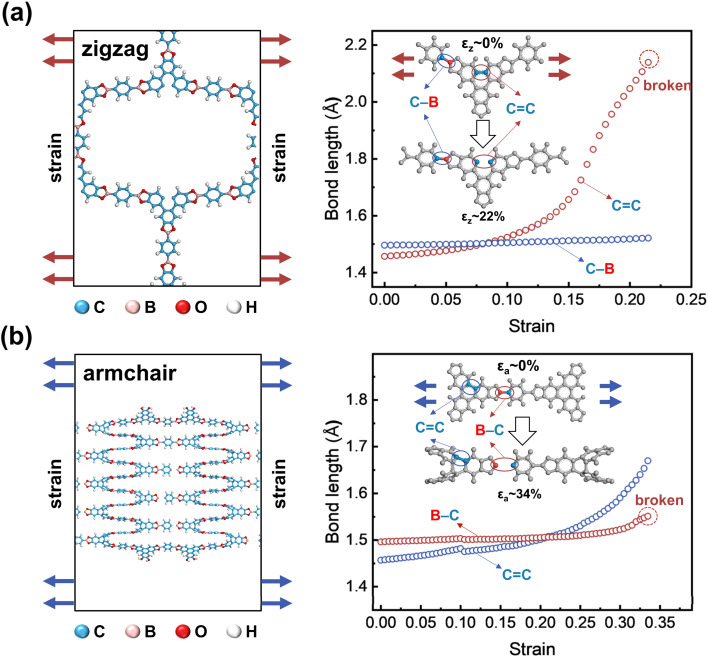
Locations of bond breakage for COF-5 stretched in the (a) zigzag and (b) armchair directions. The right panels show evolutions of the C–B and CC bond strain during stretching. The arrows indicate the directions of stretching applied.

Finally, we discussed how these four 2D COFs are fractured microscopically. Our results indicate that fractures do not occur at the locations where monomers are chemically linked (Table S8†). For instance, when COF-5 is stretched in the zigzag direction, the fracture initiates primarily within the conjugated segments of the 2,3,6,7,10,11-hexahydroxytriphenylene (HHTP) core monomer unit. As shown in Fig. 4a, the rigid triangular branching structure of the HHTP core impedes the redistribution of the local strains through dihedral angle adjustments, culminating in stress concentration zones. This exerts a significant force on one of the CC bonds, elongating it to approximately 2.1 Å—an increase of approximately 47% strain from its equilibrium length—before a fracture occurs, while the average strain across the 2D sheet remains at 21.5% (Fig. 4a). As the CC bond endures the majority of stress, the weaker C–B bonds (bond energies of C–B and CC are approximately 480–500 kJ mol^−1^ and 680–720 kJ mol^−1^, respectively; Fig. S34† and ref. 71) in the PBBA linker monomer unit are not stretched. These findings indicate the paradoxical effect of integrating rigid molecular groups into the 2D framework, which may detrimentally affect their overall mechanical strength.^72,73^

Conversely, when COF-5 is stretched along the armchair direction, local stress concentrations again emerge at the HHTP core, causing two CC bonds to stretch (Fig. 4b). However, in this case, the C–B bonds within the PBBA units, which align with the stretching direction, experience elongation. Under these conditions, the weaker C–B bonds break first when stretched to a strain of 4%, whereas the CC bonds remain intact at a strain of 15% (Fig. 4b). These observations emphasize the role of monomeric structural rigidity and bond strength disparities in determining fracture initiation sites.

For the Pc-PBBA COF, tensile stress along the edge results in the breakage of the weaker C–B bonds in the PBBA linker. Conversely, diagonal stretching induces localized stress within the tetragonally branched phthalocyanine tetra (Pc) core, leading to the cleavage of four CC bonds (Fig. S35†). In the case of Py-COF, when stretched either along the edge or short diagonal direction, the weaker C–C bonds in the terephthalaldehyde (TA) linker break (Fig. S36†). However, when stretched in the long diagonal direction, the local stress is transferred to the C–C bonds linked to the rigid structures in the 1,3,6,8-tetrakis(4-aminophenyl)pyrene (PyTTA) core, which break under high strain (Fig. S36†). For the star-pore ETTA-TPA COF, stretching along either the edge or diagonal direction leads to the breakage of the C–C bonds in the TA linker (Fig. S37†). Unlike the rigid cores found in COF-5 and the Pc-PBBA COF, the branched structures in the ETTA-TPA COF are interconnected by single bonds at the 4,4′,4′′,4′′′-(ethene-1,1,2,2-tetrayl)tetraaniline (ETTA) core. These single bonds facilitate angular adjustments, thereby preventing the formation of stress concentration zones in the conjugated cores.

### 2.4 Screening the mechanical properties of 2D COFs

**Fig. 5:**
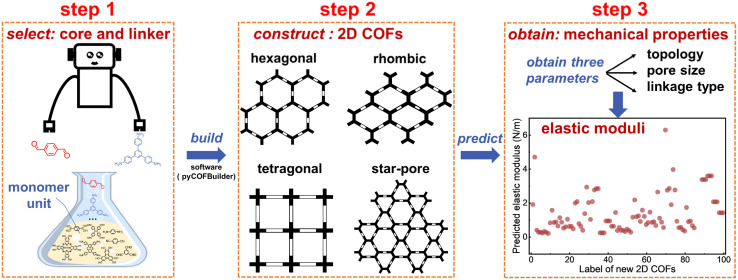
Flowchart for predicting the mechanical properties of new 2D COFs in materials design. See Fig. S37† for verification of the predicted results by DFTB calculations.

Based on the above-discovered relationships, we have developed a protocol for exploring the mechanical properties of new 2D COF structures to facilitate material design. As shown in Fig. 5, this protocol incorporates a monomer database from which cores and linkers can be selected; the 2D COF structure is then constructed, giving detailed topology, pore size, and linkage type; our discovered quantitative rules are then used to estimate the elastic modulus and Poisson's ratio. Technical details of this protocol can be found in Section 18 of the ESI.† Using this method, we efficiently predicted the mechanical properties of 100 randomly generated 2D COFs (see Fig. 5 and Tables S9, S10†).

## 3. Conclusions and outlook

In summary, we employed an efficient DFTB method to investigate the intrinsic mechanical properties of 2D COFs under tensile stress, encompassing hexagonal, tetragonal, rhombic, star-pore topologies, and chemical linkages of azine, boronate ester, boroxine, cyanovinylene, ether, hydrazone, imide, imine, oxazole, and triazine. These findings provide valuable insights into the rational design of 2D COFs with tailored mechanical properties.

Notably, at low strain (less than approximately 5%), the elastic moduli and Poisson's ratios of 2D COFs retain the characteristics of their corresponding macroscopic networks. The quantitative relationships discovered between the elastic moduli and *E*_s_*t* (or *E*_s_*t*^3^) help estimate the rigidity of 2D COFs. These relationships enable rapid estimation of elastic moduli and Poisson's ratios based on topology, pore size, and chemical linkage, thereby quantitatively informing the design of 2D COFs. For achieving high moduli in all directions, hexagonal or star-pore 2D COFs with small pore widths are desirable, particularly when employing ether or boronate ester linkages. Conversely, highly ductile 2D COFs can be designed using oxazole, triazine, imine, boroxine, and imide linkages in conjunction with larger pores. Tetragonal or rhombic topologies are recommended for designing 2D COFs with anisotropic mechanical properties.

For robust mechanical applications, the integrity of the 2D COF sheet structure must be maintained after prolonged use, necessitating that chemical bonds do not break under high strain during mechanical stretching. Fracture in 2D COFs primarily occurs at locations of local stress concentration, often away from the chemical linkages between monomer units. This phenomenon indicates that the molecular structures of monomers should be judiciously selected. Particularly, during stretching along the edge of tetragonal Pc-PBBA COF, rhombic Py-COF, and star-pore ETTA-TPA COF, or along the zigzag direction of hexagonal COF-5, elongation leads to the breaking of weaker C–C or C–B bonds in the linkers (Fig. 3a–d). Counterintuitively, integrating rigid molecular groups into a 2D framework can potentially compromise their overall mechanical strength. For diagonal or armchair direction stretching, stress concentration zones may form if rigid branched structures are employed, resulting in a high and imbalanced local strain that fractures even strong CC bonds. This susceptibility to fracture can be mitigated using soft-branched structures that allow easy angular adjustment. Additionally, tetragonal or rhombic topologies exhibit low fracture strain along the edge, indicating that their stretching should be carefully controlled to prevent breakage.

The above findings for intrinsic 2D COFs are useful in materials design, providing information on the theoretical limit of their mechanical properties. Such knowledge also serves as a solid foundation for the further understanding of the roles of extrinsic and imperfect factors, ultimately unraveling the complete mechanical properties of 2D COFs.

## 4. Methods

We apply uniaxial strain to 2D COFs. When stretched in the *x*-direction, the strain (*ε*_*x*_) is defined as:1
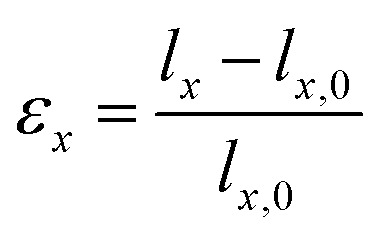
where *l*_*x*_ represents the length of the sheet at strain *ε*_*x*_ and *l*_*x*,0_ is the initial length in the *x*-direction. According to Hooke's Law, the applied force *F*_*x*_ is related to the change in sheet length by:

2 *F*_*x*_ = −*k*_*x*_(*l*_*x*_ − *l*_*x*,0_)

where *k*_*x*_ represents the elastic coefficient, which is related to the potential energy (*U*) by:3
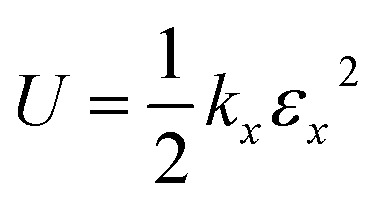


The Young's modulus (*Y*_*x*_) is the ratio of stress (*σ*_*x*_, defined as *F*_*x*_/*S*; *S* represents the interface area perpendicular to *x*-direction) to *ε*_*x*_:4
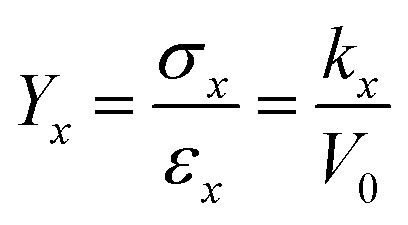
where *V*_0_ is the volume of 2D COFs, defined as *l*_*x*,0_ × *d* × *l*_*y*,0_, *l*_*y*,0_ is the initial length in the *y*-direction, *d* is the layer thickness.

Poisson's ratio (*P*_*x*_) is defined as:5
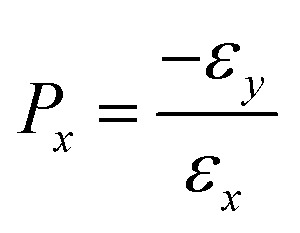


We employed the self-consistent charge approach within the density-functional tight-binding method.^74,75^ Two sets of Slater–Koster parameters, “matsci”^76^ and “mio”,^74^ were used in the calculations, encompassing “B–O–C–H” and “H–C–N–O–S” atoms, respectively. Based on our benchmarks (Fig. S1–S3†), the SCC iteration convergence criterion and maximum force component were set to 10^−5^ e and 10^−4^ hartree per bohr, respectively; the Brillouin zone was sampled using a 1 × 1 × 1 Monkhorst Pack *k*-point grid. The calculations were done using the DFTB+ software^77^ (version 21.2).

Periodic boundary condition was applied in the *x* or *y*-directions (the direction of the uniaxial tension) of 2D COFs, with a size of at least 15 angstroms in the *z*-direction (out-of-plane) to avoid interlayer interactions. We consider stretching along the main axes (zigzag, armchair, diagonal) of 2D COF models that are computationally feasible to construct. Structure optimizations were first performed for both the dimensions and atomic positions. Then, uniaxial tension was performed at a strain rate of 0.005 per step. At each step, structure optimization was performed, and the resulting structure was used to generate the initial strained coordinates for the next step. Our test by reversing this process showed that the energy profile remains the same, showing that this treatment is robust and the structure would not be struck in local minimums along the stretching path (Fig. S4†).

To further demonstrate the validity of the DFTB method for calculating these 2D COFs, we selected four representative single-pore 2D COFs containing triazine (H1), imine (H7), oxazole (H10), and boronate ester (H28) linkages, with dimensions of 5.1 × 1.5 × 3.0 nm^3^ (98 atoms), 5.0 × 1.9 × 3.0 nm^3^ (117 atoms), 5.0 × 2.1 × 3.0 nm^3^ (112 atoms), and 6.5 × 3.0 × 3.0 nm^3^ (184 atoms), respectively, and compared the results with calculations using the GGA-PBE functional^78,79^ (performed using VASP software,^80^ version 6.3). The results show that the energy profiles derived from DFTB and GGA-PBE calculations are very close up to the point of fracture (Fig. S5†).

We further tested the impact of model size on computational results. Young's moduli tend to stabilize at 4–5 pores in the direction perpendicular to the uniaxial tensile direction, as shown in Fig. S6.† Therefore, we model the 2D COFs with a 5-pore width.

## Author contributions

L. T. Xiong performed molecular dynamics calculations, analyzed the data, and contributed to writing the manuscript. C. B. Fu contributed to quantum chemistry calculations. J. X. Tian, Y. B. Geng, L. X. Han, H. Zhang, and H. Y. Li contributed to the data analysis. H. Y. Li conceived the idea, supervised the project, and contributed to writing the manuscript.

## Conflicts of interest

There are no conflicts of interest to declare.

## Supplementary Material

SC-016-D5SC02180D-s001

## Data Availability

The data that support the findings of this study are available from the corresponding authors upon reasonable request.
