# Supplementary material for: Intrinsic mechanical properties of two-dimensional covalent organic frameworks
Source: Chem Sci. 2025 Jul 21;16(35):15913–25. doi: 10.1039/d5sc02180d (PMC12290467; doi:10.1039/d5sc02180d)
Supplement: SC-016-D5SC02180D-s001 [file SC-016-D5SC02180D-s001.pdf]

Supplementary Information (SI) for Chemical Science.
This journal is © The Royal Society of Chemistry 2025

Supplementary Information

Intrinsic Mechanical Properties of Two-Dimensional Covalent Organic Frameworks

*Liangtao Xiong[a,b], Chengbin Fu[a,b], Jiaxin Tian[a], Yubo Geng[a], Lixin Han[a], Han Zhang[a], and Haoyuan Li[a,b,c]**

[a]School of Microelectronics, Shanghai University, Shanghai 201800, China

[b]Department of Chemistry, College of Sciences, Shanghai University, Shanghai 200444, China

[c]Key Laboratory of Advanced Display and System Applications, Ministry of Education, Shanghai University, Shanghai 200072, China

*Email: lihaoyuan@shu.edu.cn

Table of Contents

1. Benchmarks of the DFTB calculations S3

2. The chemical structures of 86 2D COFs in this study S10

3. Lattice parameters S25

4. Young's moduli and Poisson's ratios of 86 2D COFs in this study S27

5. Pore shapes during stretching S33

6. The internal angles of rhombic 2D COFs S34

7. The derivation of the elastic modulus of a rhombic network S35

8. The elastic moduli and Poisson's ratios of 2D COFs of simulation/measurement from literature S36

9. Calculated E_st^x for hexagonal, tetragonal, rhombic, and star-pore 2D COFs S38

10. The changes in bond lengths, angles, and dihedral angles for hexagonal 2D COFs in the armchair and zigzag directions S40

11. Poisson's ratios of 2D COFs retain the characteristics of their corresponding macroscopic networks S42

12. Stresss–train curves of 2D COFs with hexagonal, tetragonal, rhombic, and star-pore topologies. S45

13. Structures change under different strains for COF-5, Pc-PBBA COF, Py-COF, and ETTA-TPA COF S47

14. The evolution of Poisson's ratios and pore sizes of 2D COFs during stretching.S49

15. Fracture strain, ultimate strength, fracture strength, and location of fracture for selected 2D COFs S51

16. The bond energies of C–B and C=C in COF-5 ....S51

17. The locations of bond breakage for additional 2D COFs S52

18. Screening the mechanical properties of 2D COFs S56

1. Benchmarks of the DFTB calculations

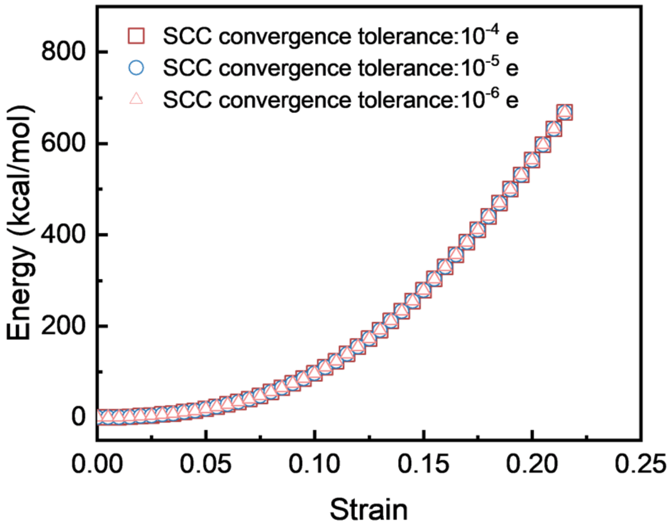

Figure S1. Potential energies of single-pore COF-5 under uniaxial tension calculated using the DFTB method with different SCC iteration convergence tolerances.

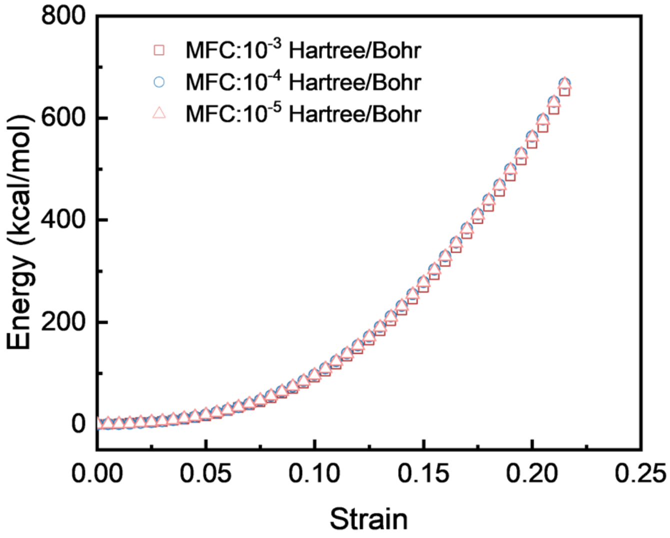

Figure S2. Potential energies of single-pore COF-5 under uniaxial tension calculated using the DFTB method with different maximum force components (MFC).

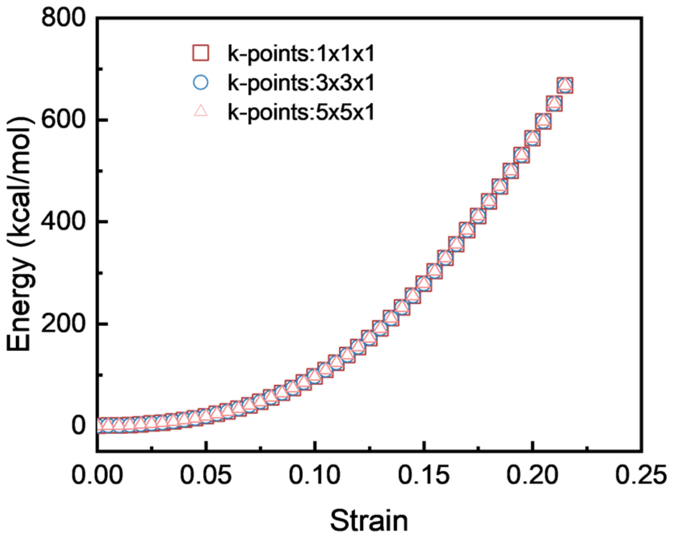

Figure S3. Potential energies of single-pore COF-5 under uniaxial tension calculated using the DFTB method with different k-point grids.

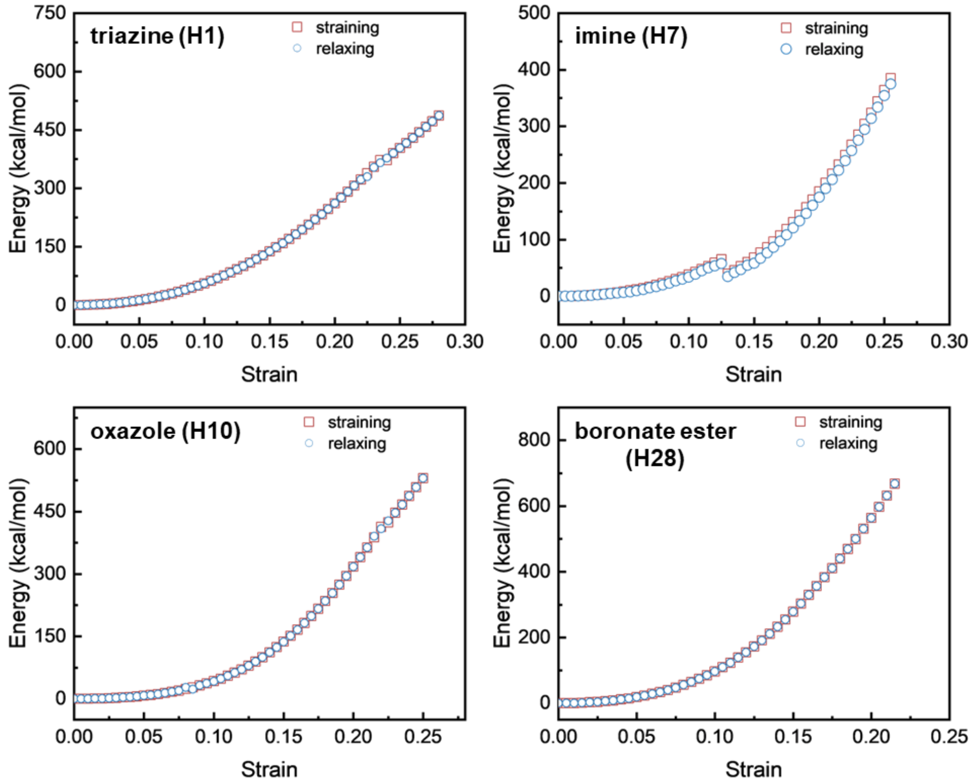

Figure S4. Calculated potential energies of four 2D COFs containing triazine (H1), imine (H7), oxazole (H10), and boronate ester (H28) chemical linkages were calculated under uniaxial tension and relaxing using the DFTB method.

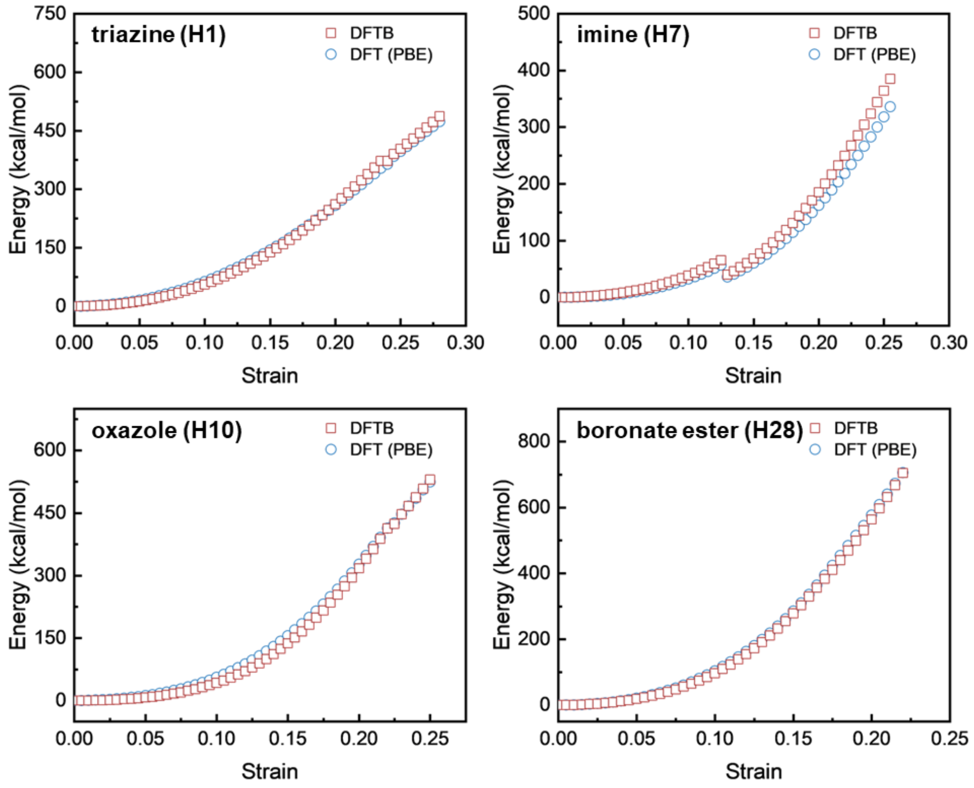

Figure S5. The potential energies of four 2D COFs containing triazine (H1), imine (H7), oxazole (H10), and boronate ester (H28) chemical linkages were calculated by DFT (GGA-PBE functional) and DFTB under uniaxial tension.

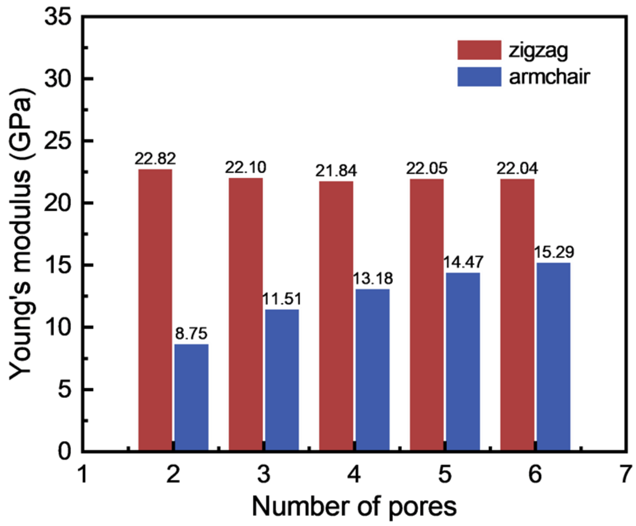

Figure S6. Calculated Young's moduli of COF-5 with different numbers of pores perpendicular to uniaxial tensile direction using the DFTB method.

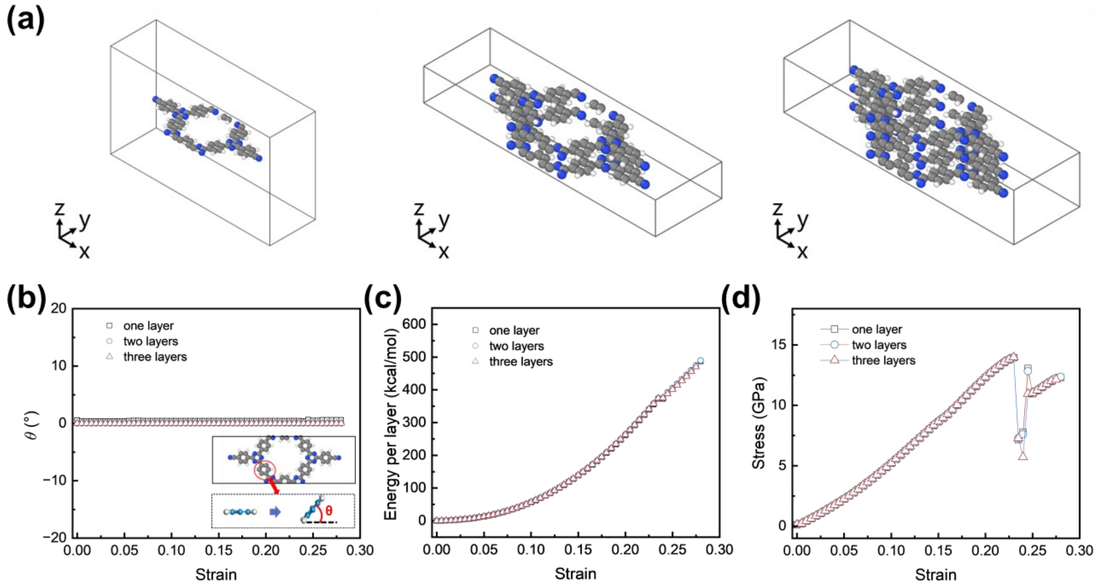

Figure S7. (a) Single-layer, two-layer, and three-layer structures of a 2D COF with triazine linkages (H1). (b) Dihedral angles of benzene rings, (c) potential energy, and (d) stress–strain curves when stressed in the zigzag direction.

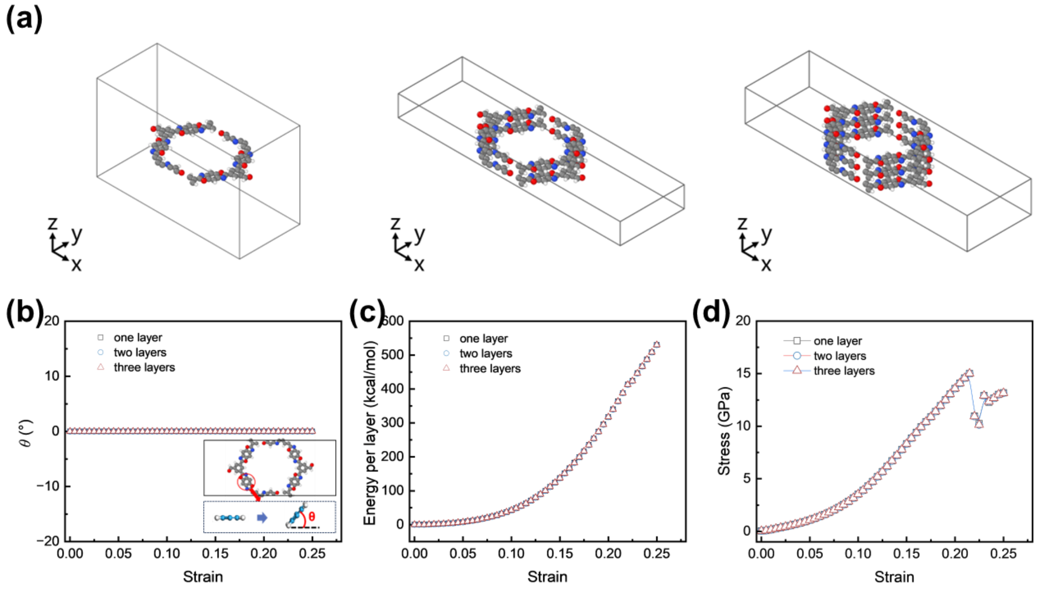

Figure S8. (a) Single-layer, two-layer, and three-layer structures of a 2D COF with oxazole linkages (H10). (b) Dihedral angles of benzene rings, (c) potential energy, and (d) stress–strain curves when stressed in the zigzag direction.

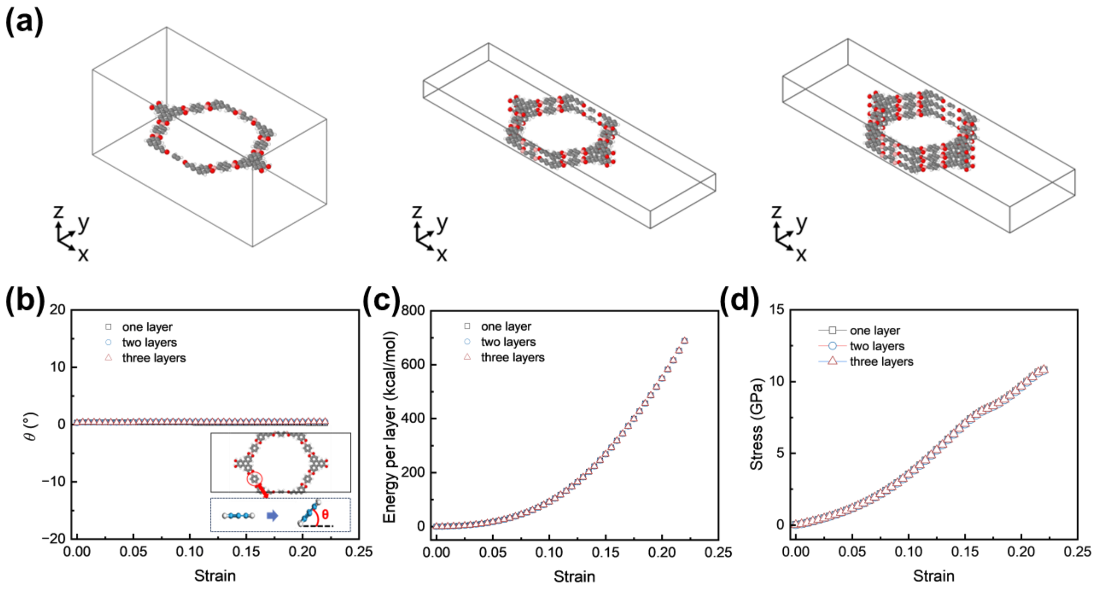

Figure S9. (a) Single-layer, two-layer, and three-layer structures of a 2D COF with boronate ester linkages (H28). (b) Dihedral angles of benzene rings, (c) potential energy, and (d) stress–strain curves when stressed in the zigzag direction.

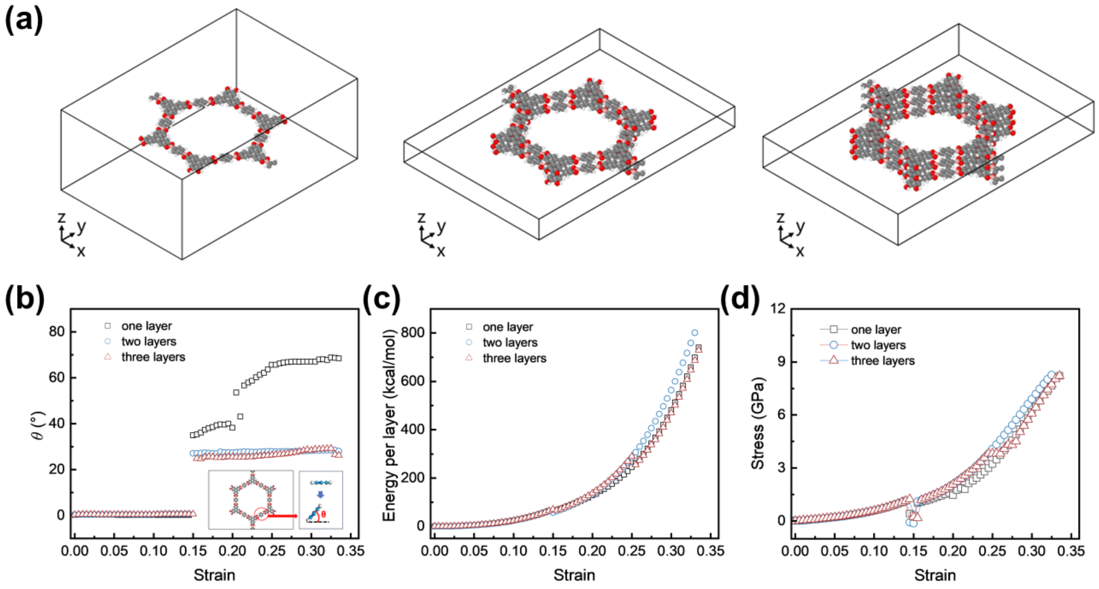

Figure S10. (a) Single-layer, two-layer, and three-layer structures of COF-5 (H28). (b) Dihedral angles of benzene rings, (c) potential energy, and (d) stress–strain curves when stressed in the armchair direction.

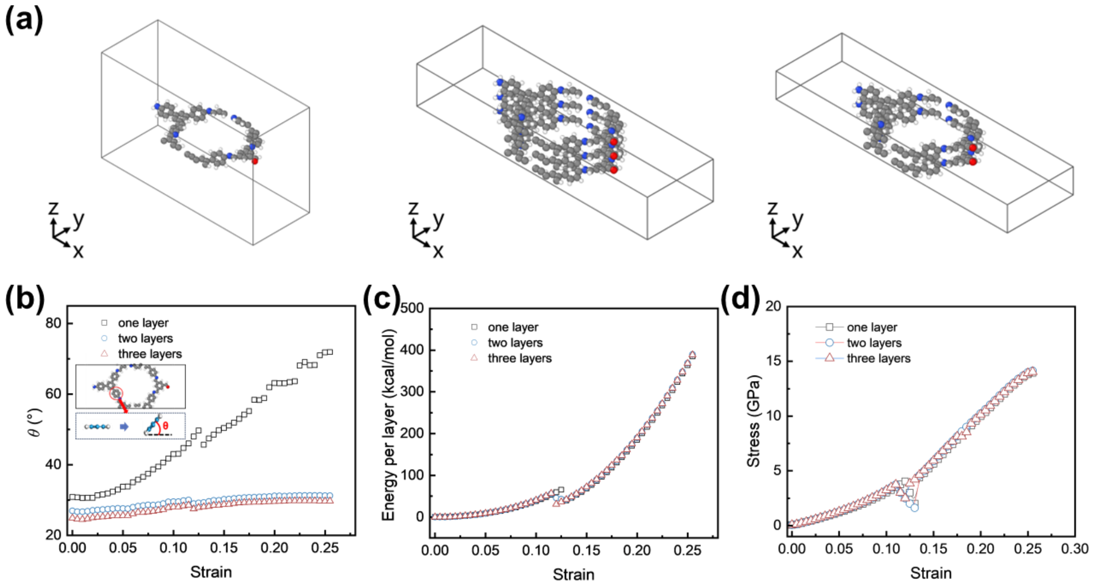

Figure S11. (a) Single-layer, two-layer, and three-layer structures of a 2D COF with imine linkages (H7). (b) Dihedral angles of benzene rings, (c) potential energy, and (d) stress–strain curves when stressed in the zigzag direction.

To further assess the accuracy of the calculation protocol, we first applied it to the extensively studied graphene. We constructed a graphene with the size of 2 nm × 11 nm (**Figure S12a**). Our calculated Young's modulus in the armchair direction is 1088 GPa (**Figure S12b**), which is consistent with the calculation result of 1020 GPa[1] obtained through uniaxial tensile with the Perdew-Burke-Ernzerhof functional. Additionally, Tan et al. simulated the Young's modulus of graphene to be 1000 GPa[2] using the Tersoff and Lennard-Jones potentials through a nanoindentation model, while Koening et al. measured the Young's modulus of graphene under vacuum deformation to be 1000 GPa[3] using atomic force microscopy. Our calculated Poisson's ratio of 0.25 (**Figure S12c**), also falls within the reported range of 0.12-0.45 in the literature.[4]

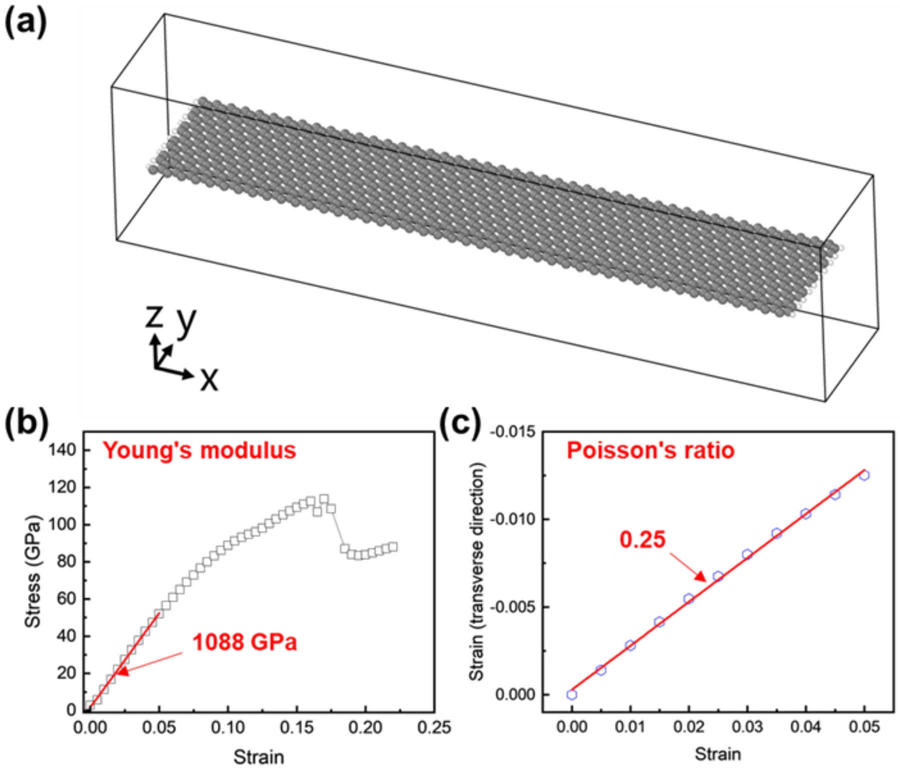

Figure S12. (a) Modeled graphene layer, the calculated (b) stress–strain curve and (c) Poisson's ratio.

2. The chemical structures of 86 2D COFs in this study

Table S1. The chemical structures of 86 2D COFs in this study.

label	chemical structure	label	chemical structure
**H1**	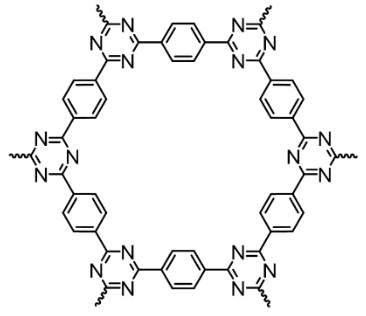	**H2**	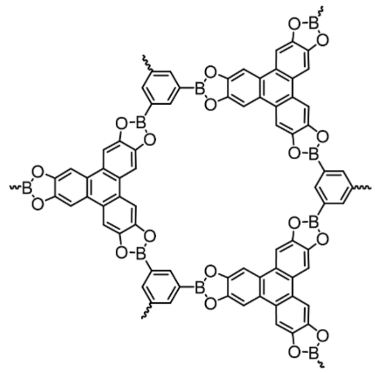
**H3**	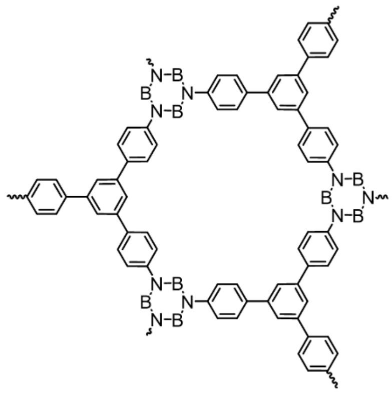	**H4**	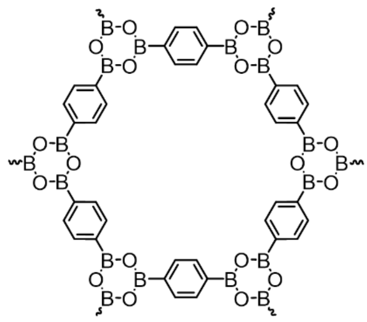
**H5**	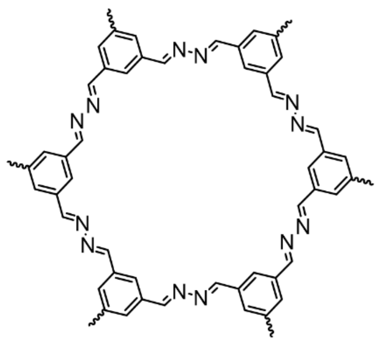	**H6**	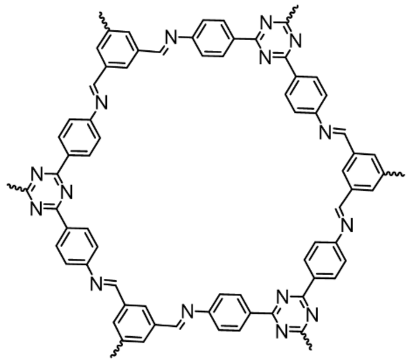

**H7**	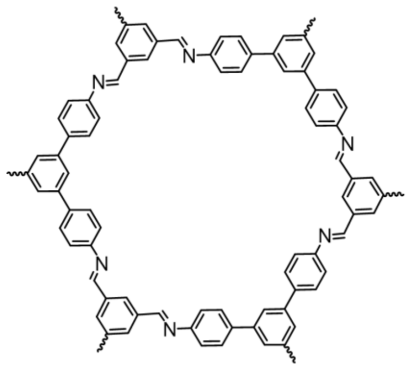	**H8**	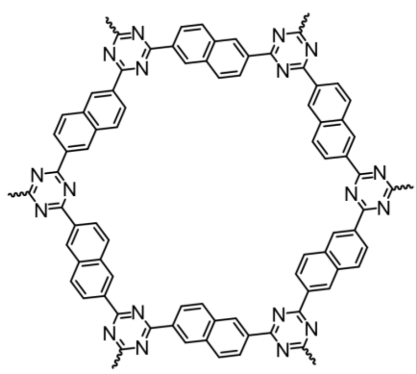
**H9**	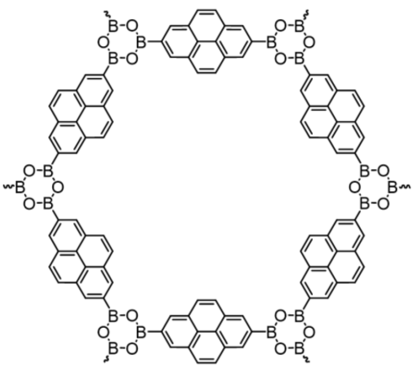	**H10**	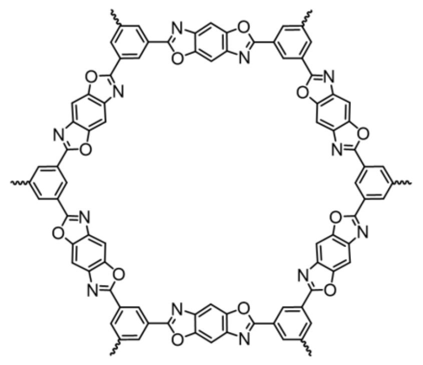
**H11**	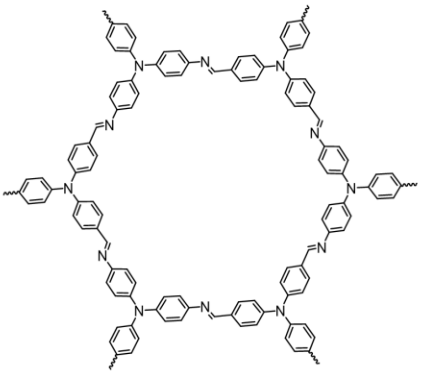	**H12**	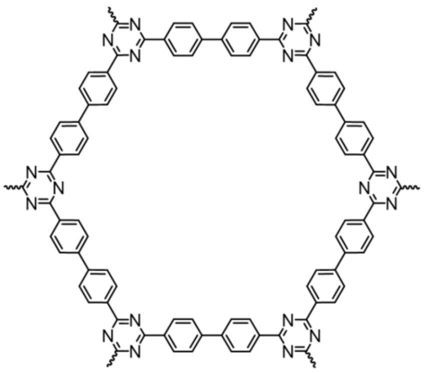

**H13**	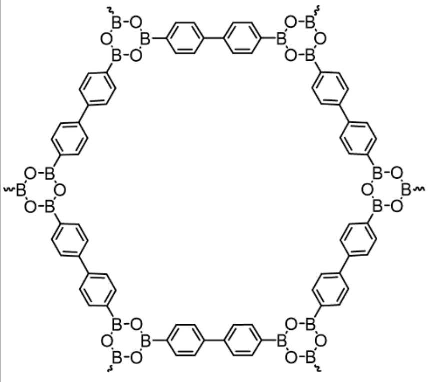	**H14**	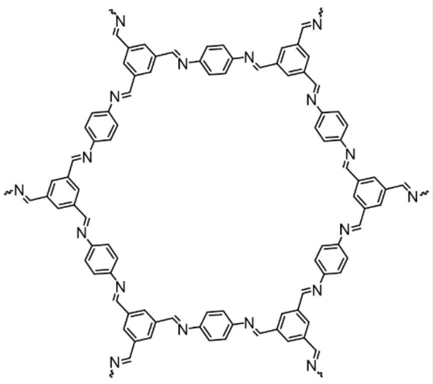
**H15**	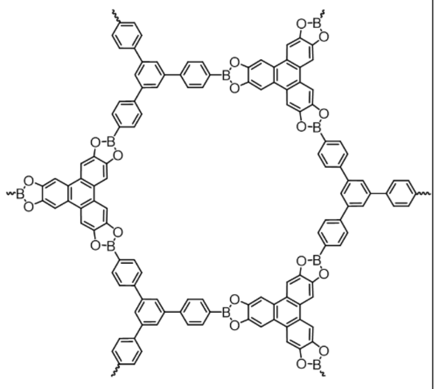	**H16**	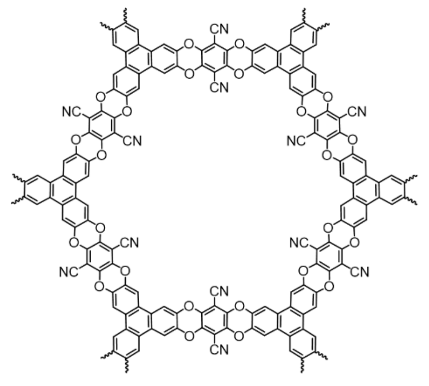
**H17**	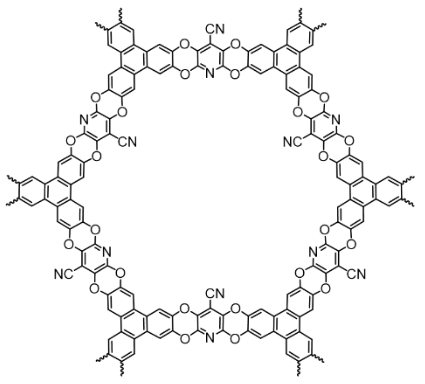	**H18**	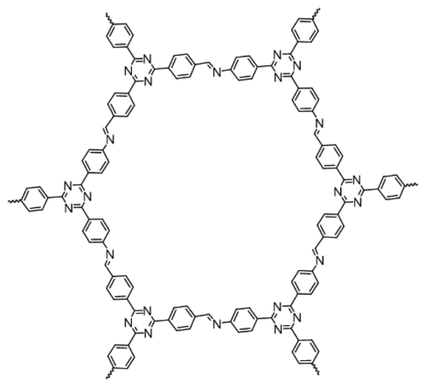

**H19**	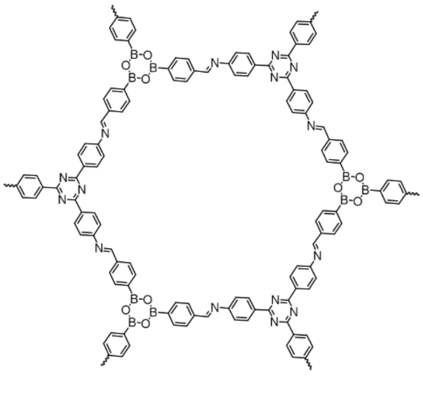	**H20**	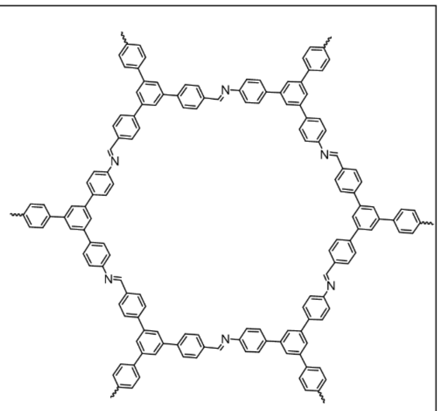
**H21**	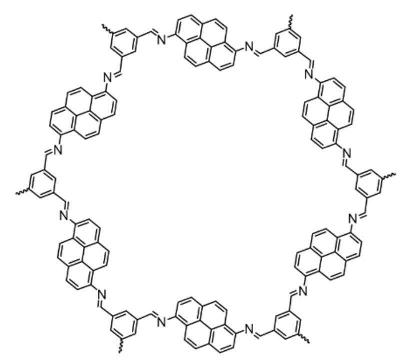	**H22**	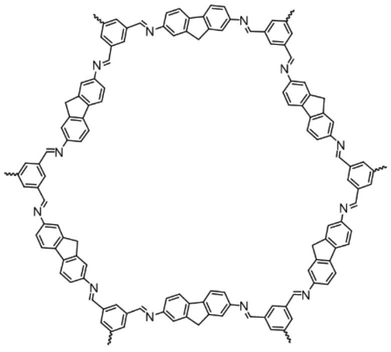
**H23**	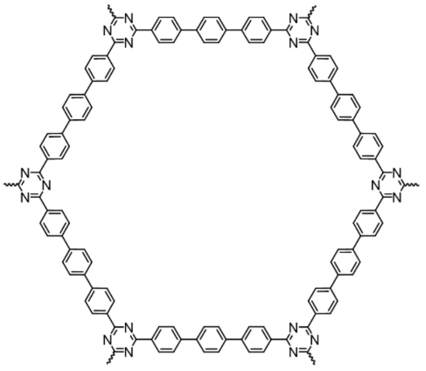	**H24**	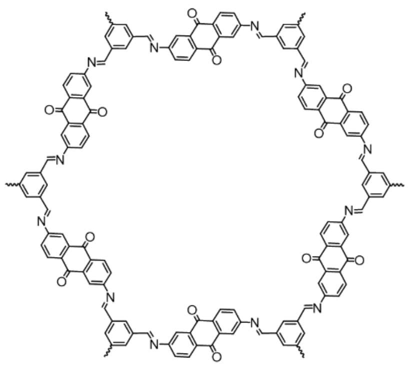

**H25**	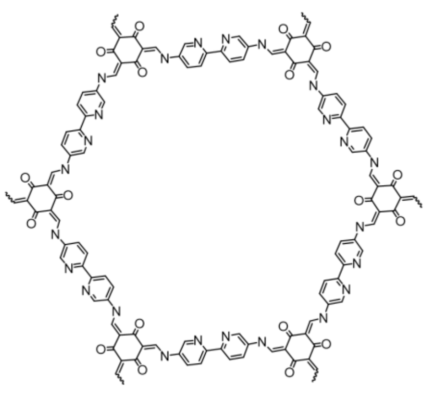	**H26**	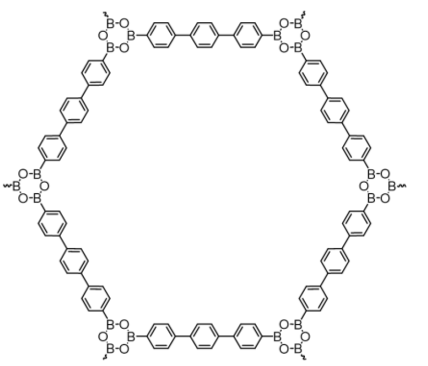
**H27**	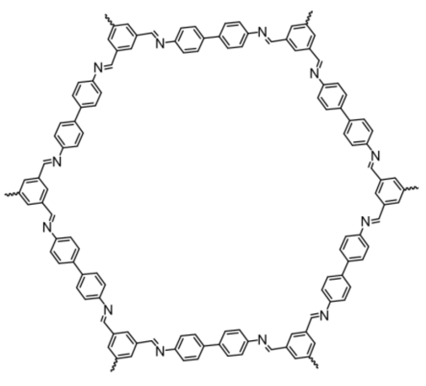	**H28**	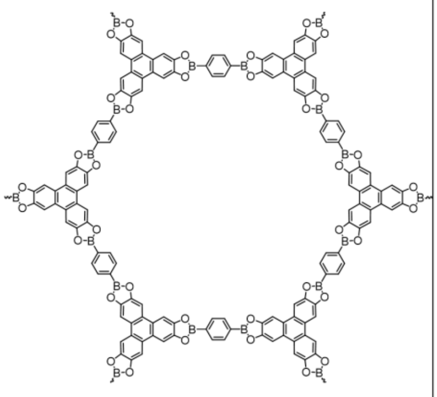
**H29**	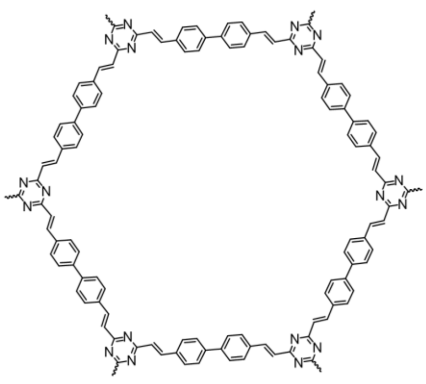	**H30**	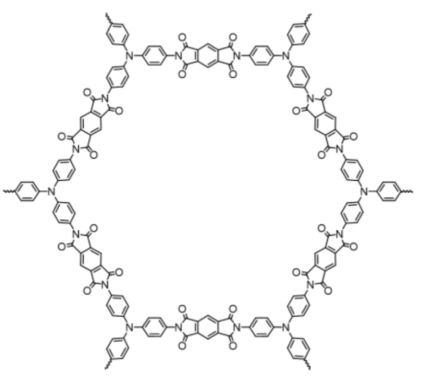

**H31**	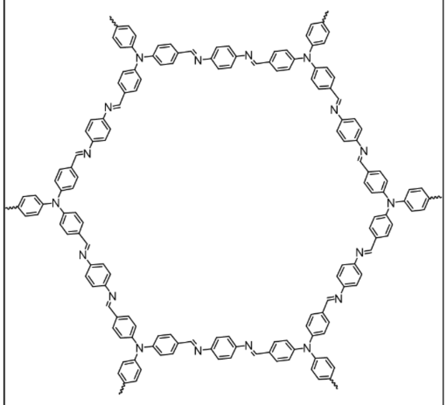	**H32**	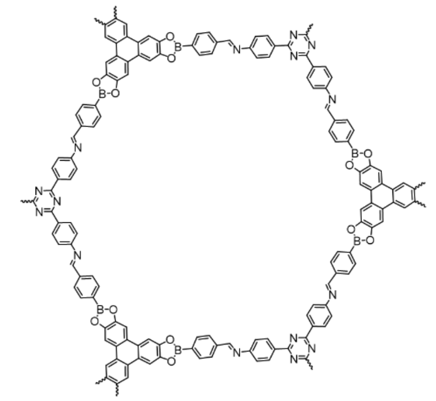
**H33**	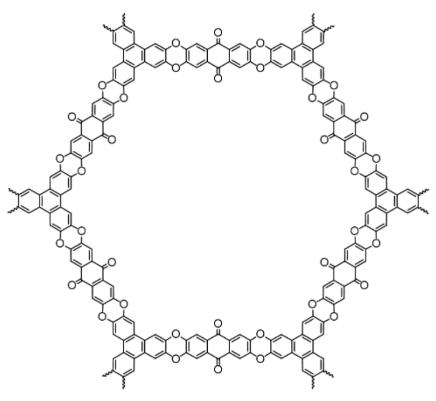	**H34**	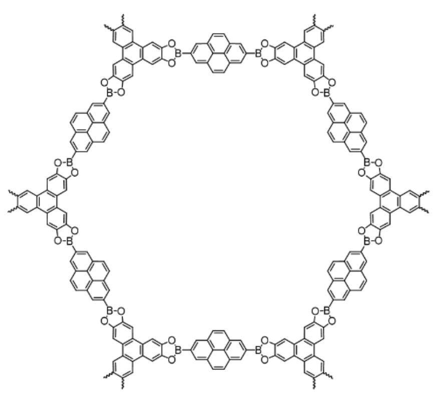
**H35**	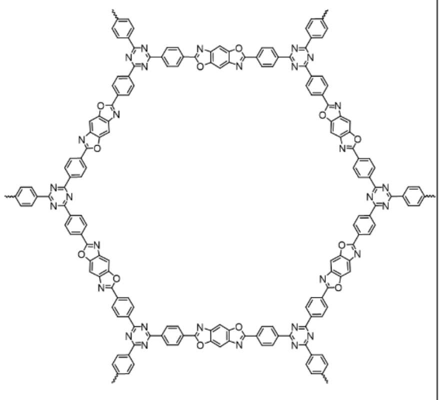	**H36**	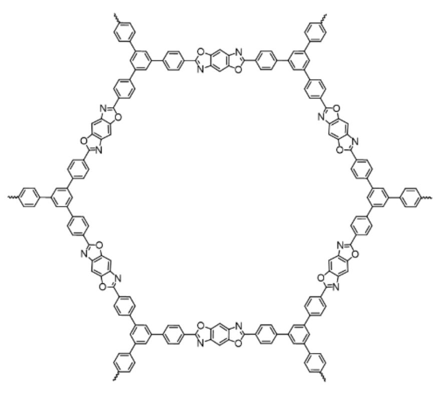

**H37**	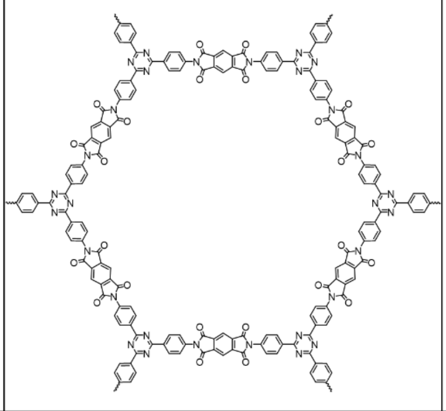	**H38**	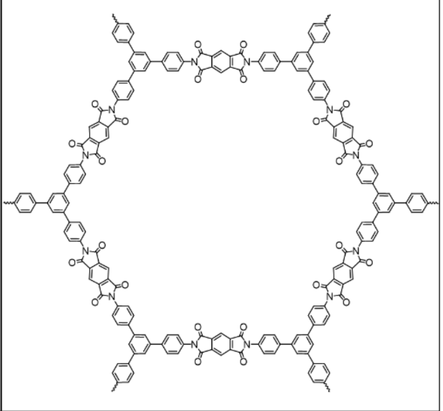
**H39**	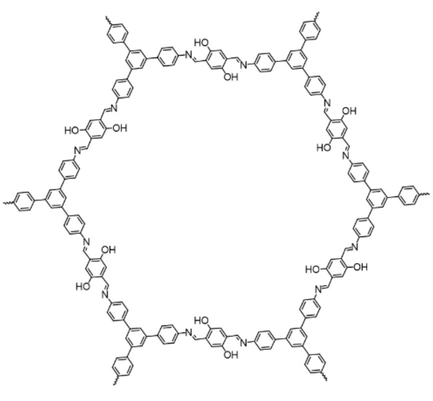	**H40**	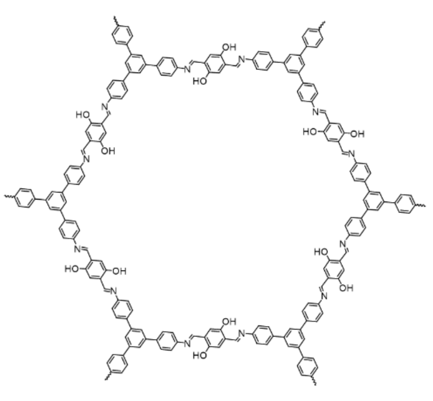
**H41**	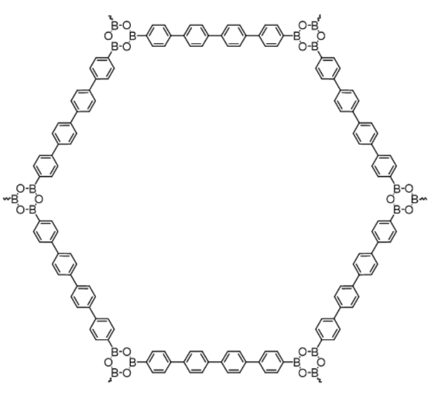	**H42**	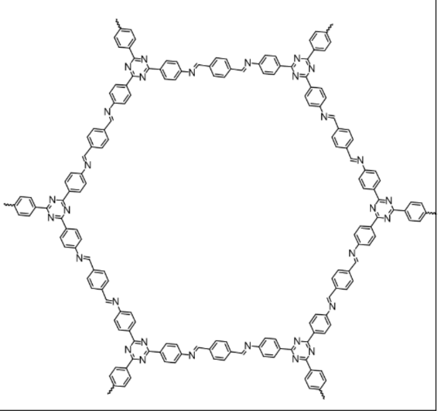

**H43**	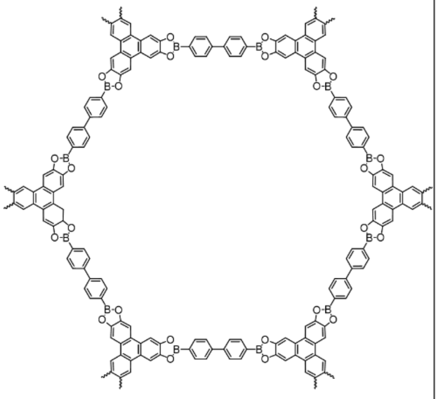	**H44**	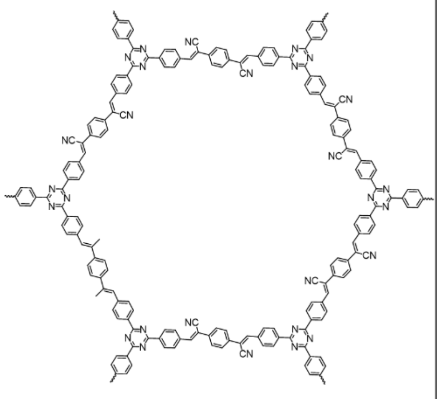
**H45**	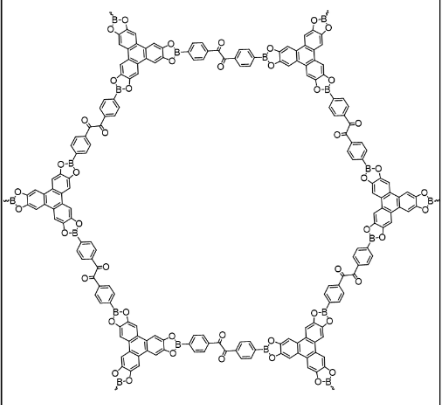	**H46**	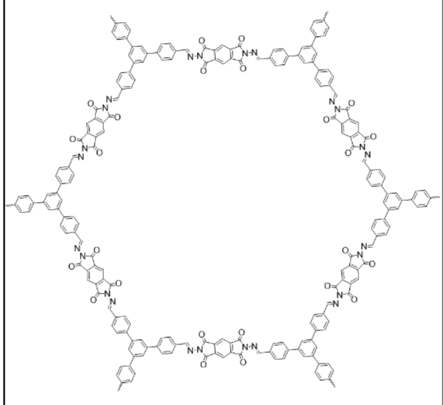
**H47**	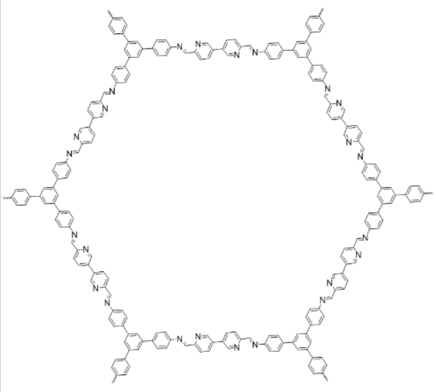	**H48**	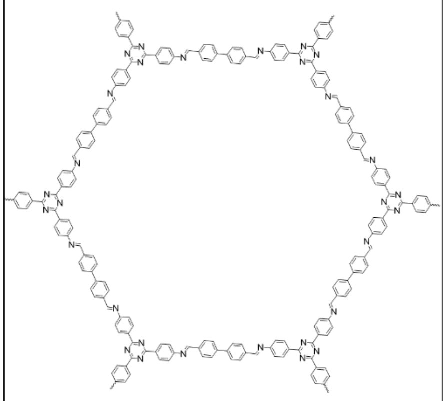

**H49**	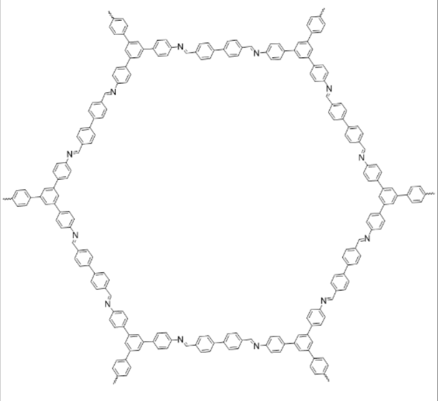	**H50**	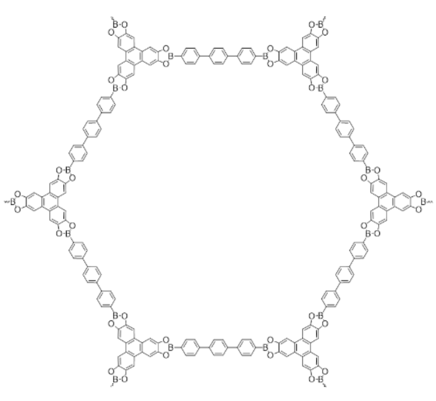
**H51**	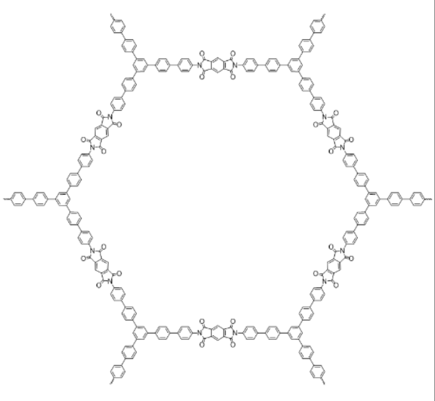	**H52**	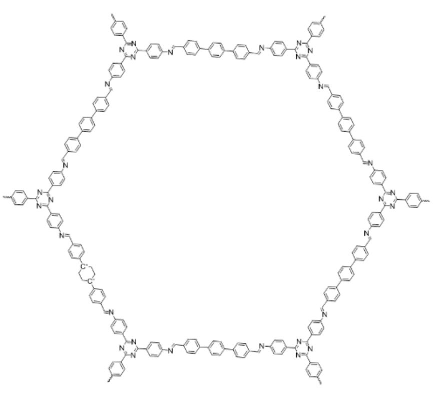
**H53**	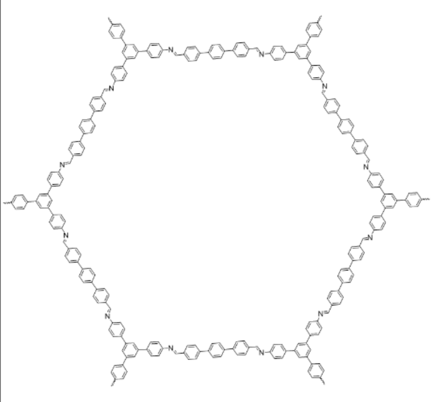	**H54**	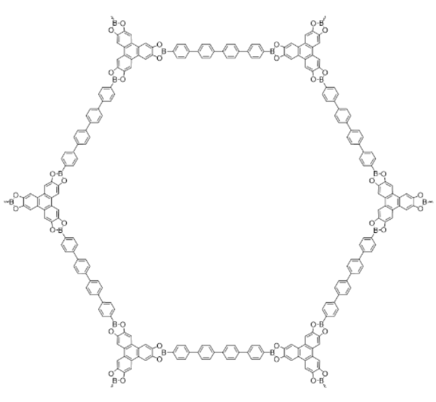

**H55**	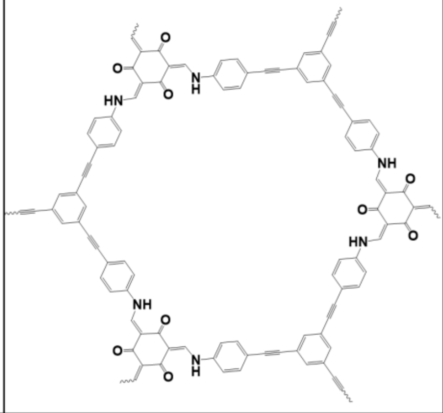	**H56**	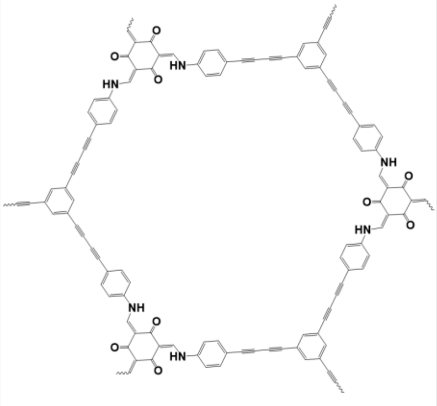
**H57**	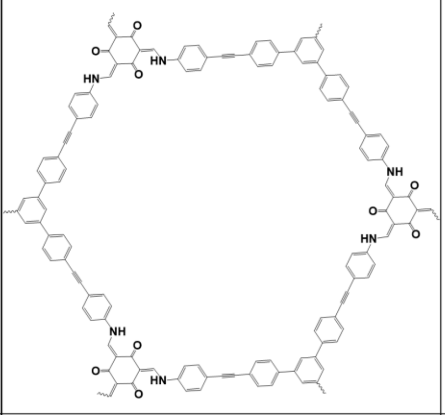	**T1**	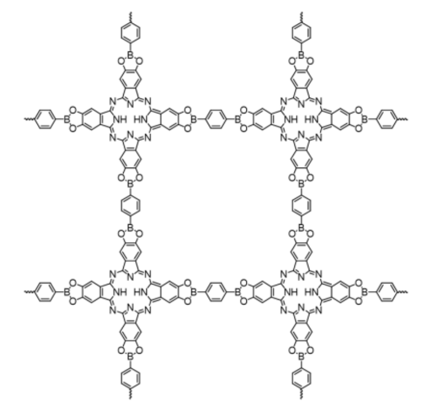
**T2**	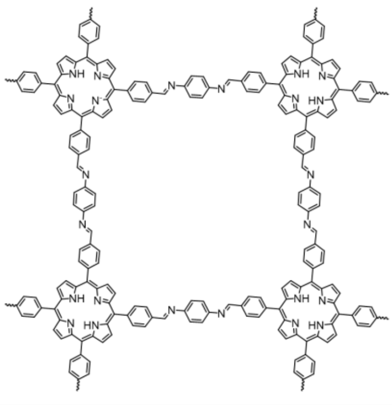	**T3**	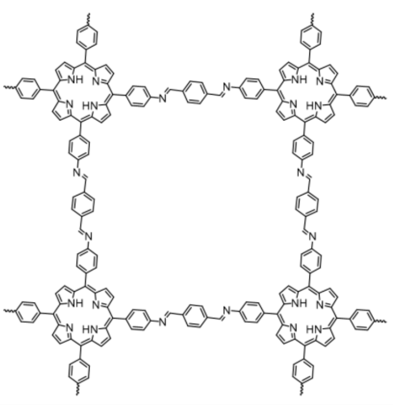

**T4**	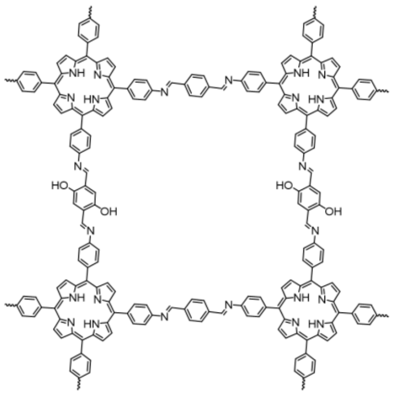	**T5**	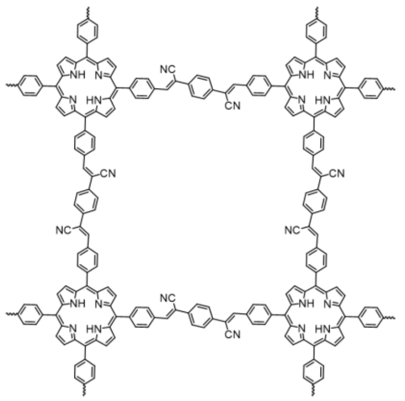
**T6**	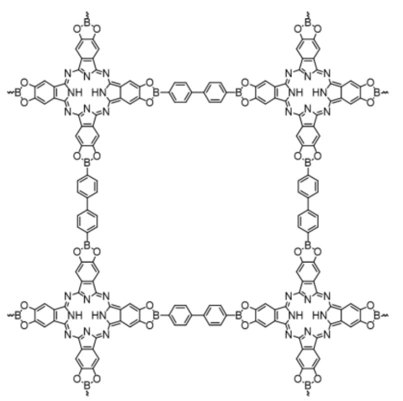	**T7**	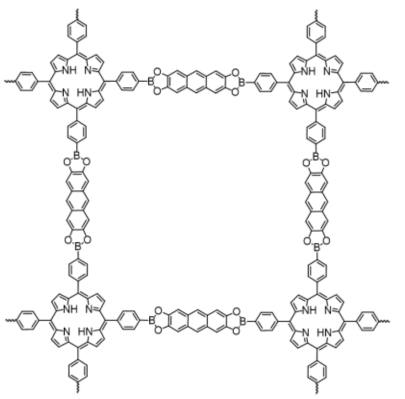
**T8**	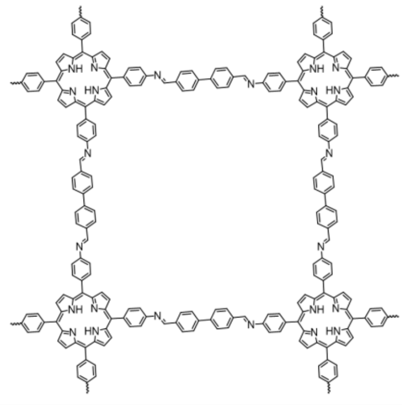	**T9**	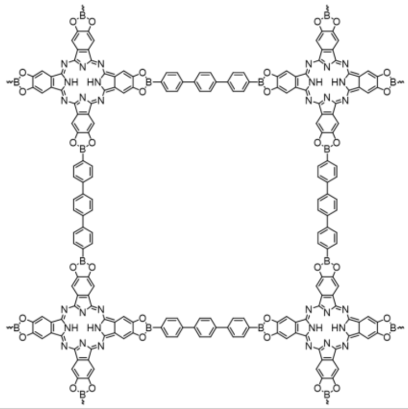

**T10**	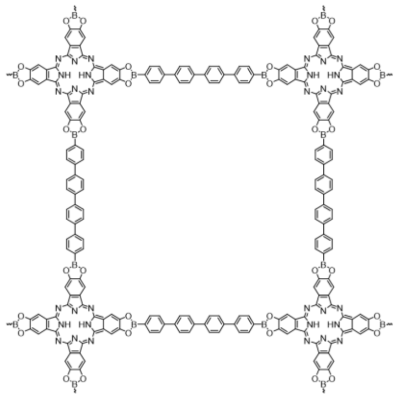	**R1**	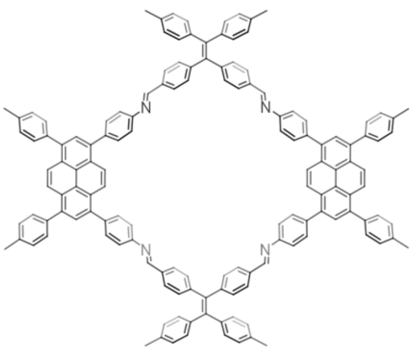
**R2**	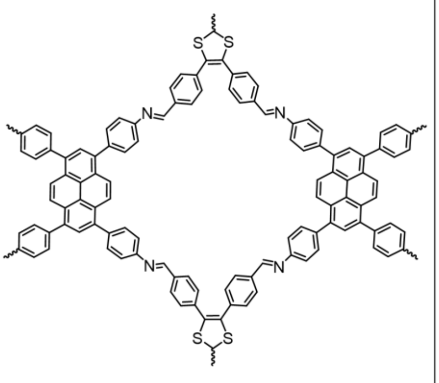	**R3**	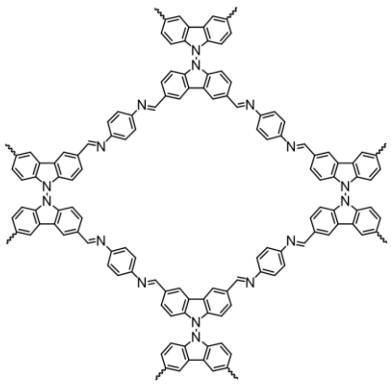
**R4**	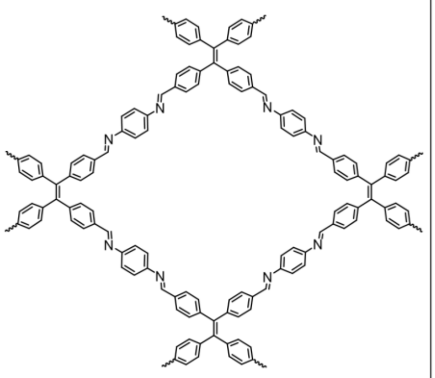	**R5**	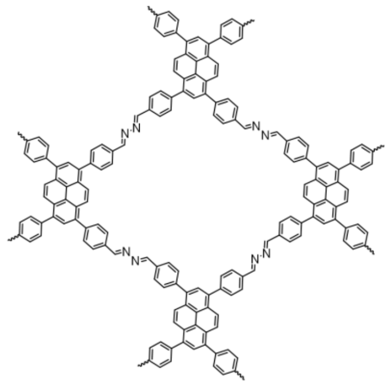

**R6**	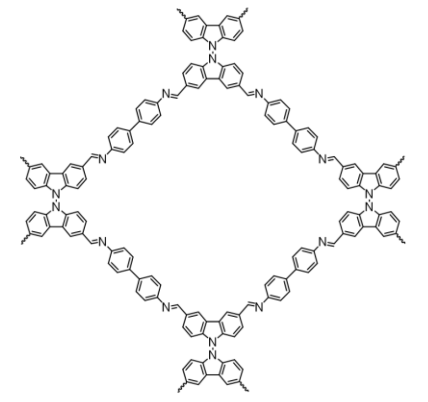	**R7**	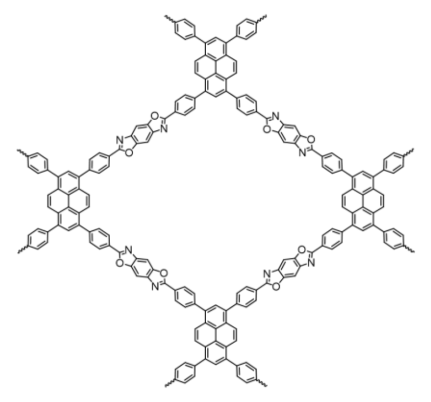
**R8**	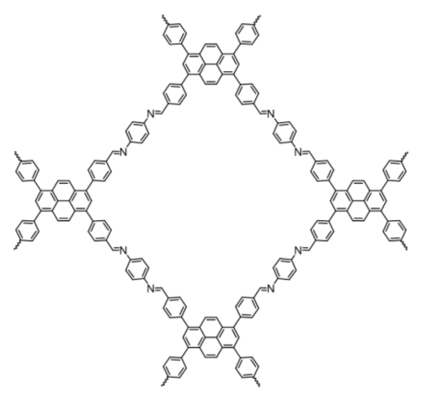	**R9**	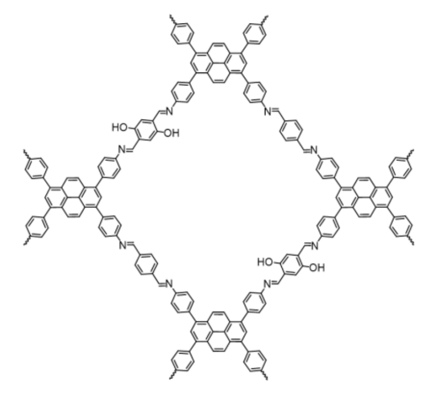
**R10**	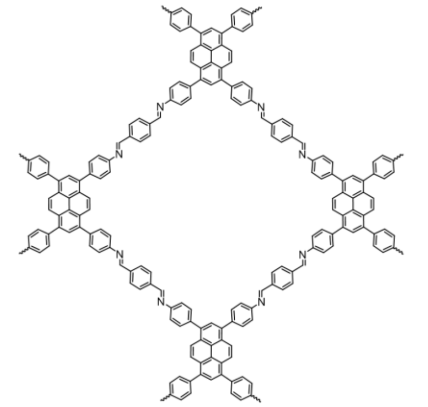	**R11**	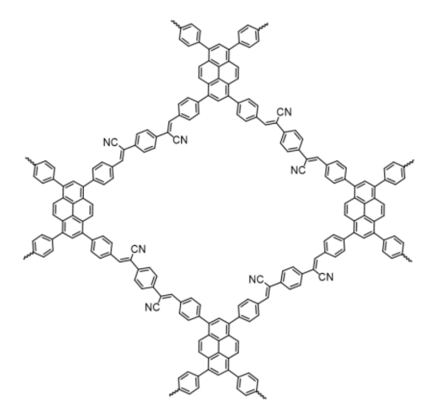

**R12**	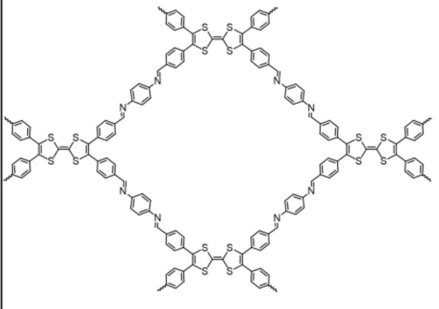	**R13**	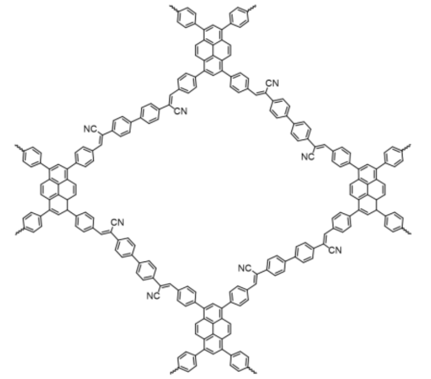
**R14**	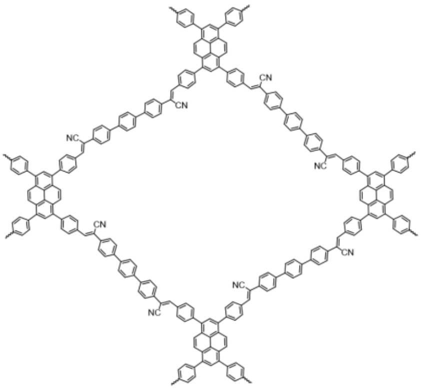	**S1**	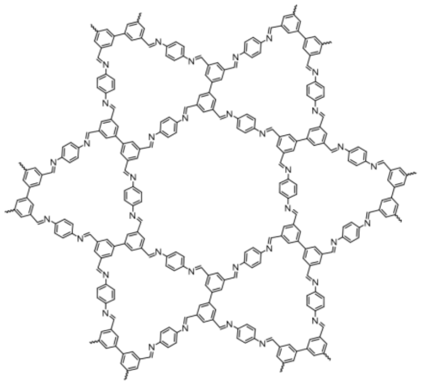
**S2**	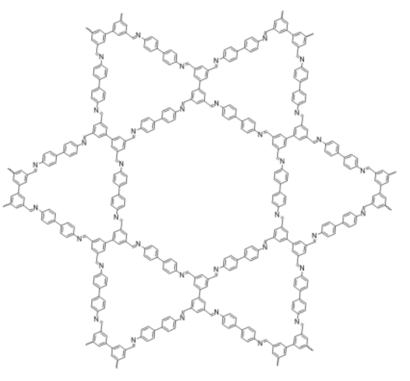	**S3**	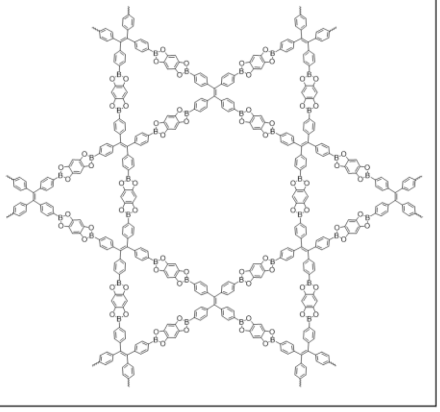

**S4**	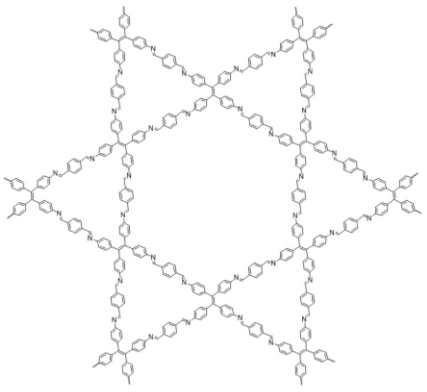	**S5**	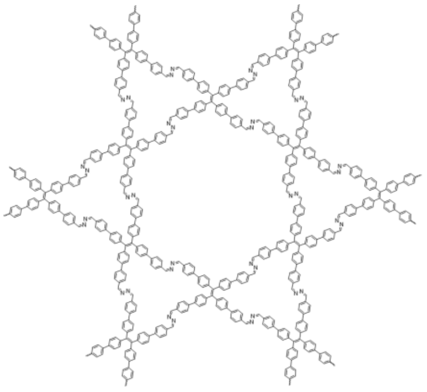

3. Lattice parameters

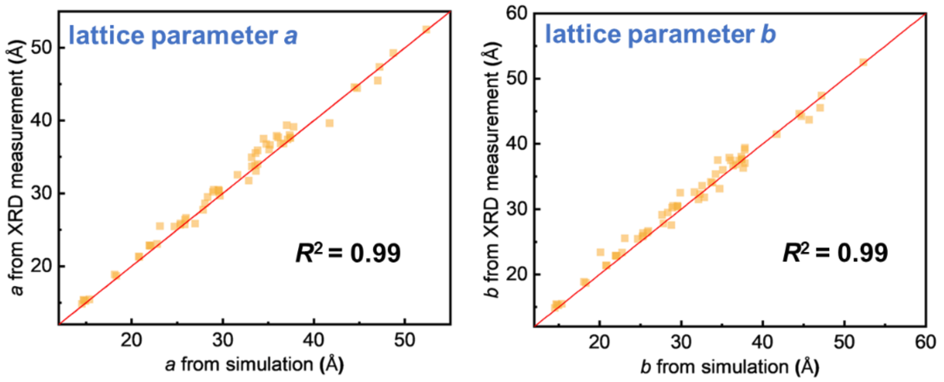

Figure S13. The lattice parameters (a) *a* and (b) *b* of the 2D COFs from simulations and experiments. The detailed data are shown in **Table S2**.

Table S2. Lattice parameters of 2D COFs.

label	in simulation			by experiment			Ref
	a (Å)	*b* (Å)	*c* (Å)	*a* (Å)	*b* (Å)	*c* (Å)	
H1	14.70	14.70	3.49	14.57	14.57	3.40	[5]
H2	15.09	15.09	3.48	14.97	14.97	3.40	[6]
H3	15.12	15.12	3.83	14.79	14.79	3.82	[7]
H4	15.29	15.29	3.52	15.42	15.42	6.66	[8]
H5	15.24	15.24	3.44	14.72	14.72	3.31	[9]
H6	18.71	18.71	3.44	18.16	18.16	3.50	[10]
H7	18.96	18.96	3.44				
H8	18.52	18.52	3.44	18.36	18.36	3.36	[11]
H9	22.66	22.66	3.48	22.16	22.16	3.42	[12]
H10	21.17	21.17	3.45	20.83	20.83	3.45	[13]
H11	21.17	21.17	4.43	20.92	20.92	3.92	[14]
H12	22.15	22.15	3.50				
H13	22.91	22.91	3.49				
H14	22.70	22.70	3.45	22.04	22.04	3.73	[15]
H15	22.72	22.72	3.47	22.01	22.01	3.63	[6]
H16	25.31	25.31	3.44	24.77	24.77	3.46	[16]
H17	25.38	25.38	3.44	23.16	23.16	2.92	[17]
H18	25.56	26.18	3.48	25.41	25.41	3.61	[18]
H19	26.26	26.26	3.47	25.94	25.92	3.54	[19]
H20	26.50	26.50	3.44	26.03	26.03	3.63	[20]
H21	27.64	27.64	3.44	27.90	27.90	3.76	[21]
H22	29.37	29.37	3.73	28.44	28.44	3.49	[22]
H23	29.61	29.61	3.51				
H24	30.30	30.30	3.45				
H25	30.43	30.43	3.43	29.3	29.3	3.5	[23]

H26	30.53	30.53	3.48				
H27	30.30	30.30	3.45	29.66	29.66	3.45	[24]
H28	30.19	30.19	3.48	29.70	29.70	3.46	[8]
H29	30.40	30.40	3.45	29.10	29.10	6.90	[25]
H30	31.66	31.66	4.02	32.93	32.93	3.43	[26]
H31	32.47	32.47	4.14	31.71	31.71	10.25	[27]
H32	32.97	34.02	3.50	33.73	33.73	3.50	[19]
H33	33.96	33.96	3.44	33.94	33.94	3.57	[16]
H34	37.49	37.49	3.47	37.54	37.54	3.38	[28]
H35	35.93	35.93	3.45	35.19	35.19	3.34	[13]
H36	36.44	36.44	3.45				
H37	36.76	36.76	3.44	36.81	36.81	3.48	[29]
H38	37.33	37.33	3.44	37.23	37.23	3.44	[26]
H39	37.63	37.40	3.58	36.20	36.20	3.40	[30]
H40	37.40	37.60	3.58				
H41	38.15	38.15	3.47				
H42	37.43	37.43	3.45	34.60	34.60	4.00	[31]
H43	37.81	37.81	3.47	36.03	36.03	3.53	[6]
H44	37.62	37.62	3.51	37.53	37.53	3.50	[32]
H45	39.59	41.39	3.65	41.86	41.86	3.39	[33]
H46	44.52	44.52	3.44	44.60	44.60	3.50	[34]
H47	44.39	44.19	3.58	44.88	44.88	3.55	[35]
H48	44.93	44.93	3.45				
H49	45.44	45.44	3.45	47.16	47.16	3.51	[36]
H50	45.42	45.42	3.47				
H51	52.46	52.46	3.44	52.51	52.51	3.44	[26]
H52	52.47	52.47	3.45				
H53	52.97	52.97	3.45				
H54	53.04	53.04	3.47				
H55	23.18	23.18	3.52	23.18	23.18	3.52	[37]
H56	27.69	27.69	3.53	25.10	25.10	3.34	[37]
H57	30.61	30.61	3.52	27.75	27.75	3.29	[37]
T1	22.92	23.20	3.48	22.85	22.85	3.34	[38]
T2	25.63	25.66	4.14	25.91	25.32	7.59	[39]
T3	25.75	25.76	4.14	25.42	25.42	5.64	[40]
T4	36.58	36.24	4.14	35.30	37.72	3.89	[41]
T5	36.78	36.55	4.16	36.48	36.48	4.20	[42]
T6	27.31	27.48	3.51				
T7	30.10	30.10	3.95	28.98	28.98	3.81	[40]
T8	30.05	30.02	4.07				
T9	31.61	31.79	3.51				
T10	35.85	36.30	3.54				
R1	22.60	20.62	5.41				
R2	26.29	23.25	4.05	36.74	20.16	3.69	[43]

R3	25.70	28.98	3.82	27.07	27.74	3.99	[44]
R4	25.91	29.20	5.48				
R5	28.52	27.46	3.92	28.14	28.89	3.91	[45]
R6	34.86	31.38	3.92	33.27	32.22	3.98	[44]
R7	33.75	32.40	3.79	33.65	29.97	3.51	[13]
R8	36.70	32.21	3.86	34.88	32.41	7.88	[46]
R9	35.46	33.44	3.87	33.72	32.67	3.82	[47]
R10	33.64	35.28	3.85	33.30	34.30	3.88	[48]
R11	35.83	33.02	3.95	33.93	34.82	3.77	[49]
R12	29.57	36.95	4.39	29.75	37.92	4.28	[50]
R13	42.47	38.58	3.79				
R14	49.25	43.63	3.78	48.89	45.81	3.68	[49]
S1	30.39	30.39	3.46	29.61	29.61	3.56	[51]
S2	39.30	39.30	3.44	37.14	37.95	3.62	[51]
S3	37.94	37.94	5.10	37.47	37.47	4.45	[52]
S4	39.04	39.04	5.46	37.90	37.90	4.80	[53]
S5	47.34	47.34	5.53	47.35	47.35	4.73	[54]

Note: Some 2D COFs do not have experimentally reported lattice parameters.

4. Young's moduli and Poisson's ratios of 86 2D COFs in this study

Table S3. Calculated Young's moduli and Poisson's ratios of 2D COFs under tensile stress (in the strain range of < 5%).

pore shape	label	name	pore width (Å)	thick ness (Å)	number of atoms	elastic modulus (N/m)	Young's modulus (GPa)	Poisson's ratio	Ref
					zigzag/ armchair	zigzag/ armchair	zigzag/ armchair	zigzag/ armchair	
hexagonal	H1	CTF-1	12.1	3.4	444/490	16.8/11.7	49.4/34.3	1/0.8	[5]
	H2	COF-6	12.2	3.4	534/628	36.6/29	107.8/85.4	0.7/0.8	[6]
	H3	BLP-2	12.4	3.8	543/600	21.8/19.8	57.2/51.7	0.7/0.7	[7]
	H4	COF-1	12.5	3.4	620/700	12.8/9	37.8/26.6	1/1	[8]
	H5	ACOF-1	15.1	3.3	592/644	11.8/8.9	35.8/26.9	0.8/0.8	[9]
	H6	N3-COF	16.1	3.5	744/840	9.6/6.5	27.5/18.7	0.9/1	[10,55]
	H7	NP@c-1	16.5	3.4	650/706	6.3/4.6	18.5/13.5	1/1	[56]
	H8	CTF-2	17.1	3.4	836/916	7.9/6	23.4/17.8	1/0.9	[11]
	H9	PPy-COF	17.4	3.4	813/897	4.2/3.2	12.4/9.3	1.1/1.1	[12]
	H10	LZU-190/B-BOP	18.2	3.5	873/963	6/4.3	17.4/12.4	1.1/1.1	[13,57]
	H11	TPA-COF	18.9	3.9	969/1047	3.3/2.3	8.3/5.8	1.1/1.1	[14]
	H12	CTF-1-2R	19.7	3.4	998/1078	4.7/3.6	13.8/10.6	1/1	
	H13	BP-	20.0	3.4	1060/12	3.4/2.8	9.9/8.4	1.1/1.1	[58]

	COF/COF-1-2R			02				
H14	LUZ1	20.0	3.7	952/1040	3.7/3	10.1/8.2	1.1/1	[15]
H15	COF-8	20.1	3.6	1148/1252	10.6/7.5	29.1/20.6	0.9/0.9	[6]
H16	COF-316 /JUC-505	22.1	3.5	1071/1170	16.5/13.4	47.2/38.2	0.9/0.8	[16,17]
H17	COF-318	22.3	2.9	1358/1474	18.1/14.5	62.1/49.8	0.9/0.8	[17]
H18	TTI-COF/TTA-TTB COF	22.3	3.6	1192/1304	6.6/2	18.2/5.6	1.4/0.7	[18,59]
H19	TATTA-FPBA COF	23.4	3.5	1308/1428	2.8/2.3	7.9/6.4	1.1/1	[19]
H20	TFPB-TAPB-COF	23.7	3.6	1308/1428	3.1/2.1	8.6/5.8	1.1/1.1	[20]
H21	IMDEA-COF-1	23.8	3.8	1190/1300	1.2/1.8	3.3/4.8	1.1/1	[21]
H22	FL-COF-1	27.0	3.5	1306/1424	2.9/1.7	8.3/4.8	1.4/1.2	[22]
H23	CTF-1-3R	27.2	3.4	1482/1654	2.4/1.8	7/5.2	1.1/1	
H24	DAAQ-BTA COF	27.3	3.4	1480/1610	1.7/1.3	5.1/3.7	1.1/1.1	[60]
H25	TpBpy	27.3	3.5	1532/1660	3.7/2.4	10.6/6.8	1/1	[23,61]
H26	COF-1-3R	27.4	3.4	1770/1920	1.4/1	4.1/3	1.1/1.1	
H27	N2-COF	27.5	3.4	1822/1970	1.7/1.3	5/3.8	1.2/1.1	[24,55]
H28	COF-5	27.7	3.4	444/490	7.5/4.9	22.1/14.5	1.1/1.1	[8]
H29	COF-701	27.9	6.9	534/628	2.5/1.9	3.7/2.8	1.2/1.2	[25]
H30	PI-COF-1	28.5	3.43	543/600	1.1/0.7	3.3/2.1	1.2/1.1	[26]
H31	IISERP-COF2	29.6	10.25	620/700	0.9/0.7	0.9/0.7	1.2/1.1	[27]
H32	HHTP-FPBA-TATTA COF	30.0	3.5	592/644	5.7/1.4	16.3/4	1.5/0.8	[19]
H33	JUC-506	31.0	3.6	744/840	8.3/6.4	23.3/17.9	1/1	[16]

H34	TP-COF	32.5	3.4	650/706	2.7/1.8	8/5.3	1.2/1.1	28
H35	LZU-191/BBO-COF 3	32.8	3.3	836/916	1.5/1.1	4.6/3.4	1.2/1.1	13,62
H36	TPB-BOP/BBO-COF-2	33.2	3.4	813/897	2.6/1.8	7.8/5.4	1.1/1.1	57,62
H37	TS-COF-1	33.5	3.5	873/963	0.2/0.2	0.6/0.7	1.2/1.1	29
H38	COF-PI2	33.6	3.4	969/1047	1/0.8	3.0/2.3	1.2/1.1	26
H39	COF-DhaTab	34.8	3.4	998/1078	0.8/0.6	2.5/1.7	1.2/1.1	30
H40	TAPB-DHTA COF	34.8	3.4	1060/1202	0.8/0.6	2.4/1.7	1.2/1.1	63,64
H41	COF-1-4R	34.9	3.4	952/1040	0.8/0.6	2.2/1.6	1.2/1.1	
H42	TRITER-1	35.2	4	1148/1252	1.5/1.1	3.9/2.9	1.2/1.2	31
H43	COF-10	35.2	3.5	1071/1170	3.5/2.2	9.9/6.2	1.2/1.2	6
H44	TP-COF	35.3	3.5	1358/1474	1.3/0.9	3.7/2.7	1.1/1.1	32
H45	BZL-COF	38.4	3.4	1192/1304	0.8/0.4	2.5/1.1	0.9/0.7	33
H46	TfpBDH	41.0	3.5	1308/1428	1/0.7	2.8/2.0	1.2/1.1	34
H47	LUZ-20	41.9	3.6	1308/1428	0.9/0.4	2.5/1.2	1.2/1.1	35
H48	TTI-COF-2R	42.0	3.6	1190/1300	0.8/0.5	2.3/1.5	1.3/1.2	
H49	TAPB-BPDA	42.5	3.5	1306/1424	0.8/0.6	2.3/1.6	1.3/1.2	36
H50	COF-5-3R	42.7	3.4	1482/1654	1.5/1	4.6/2.9	1.2/1.2	
H51	PI-COF-3	49.1	3.4	1480/1610	0.7/0.5	1.9/1.4	1.4/1.2	26
H52	TTI-COF-3R	49.6	3.6	1532/1660	0.5/0.3	1.5/0.9	1.3/1.2	
H53	LUZ-1-3R	49.8	3.7	1770/1920	0.3/0.2	0.8/0.6	1.2/1.1	
H54	COF-5-4R	50.1	3.4	1822/1970	1/0.6	2.9/1.6	1.1/1.2	
H55	S1-TP COF	20.8	3.52	663/732	3.9/2.6	11.0/7.4	1/0.7	37
H56	S2-TP	25.3	3.34	723/798	1.9/1.2	5.6/3.5	1.1/0.8	37

	H57	COF S3-TP COF	28.2	3.29	963/1062	2.3/1.4	7.0/4.2	1.1/0.7	[37]
					along edge/ diagona	along edge/ diagonal	along edge/ diagonal	along edge/ diagonal	
	T1	Pc-PBBA COF	20.7	3.3	960/878	46.9/2.6	140.4/7.7	0.3/1	[38]
	T2	Por-COF	23.0	7.6	1252/1158	15/0.6	19.7/0.8	0.3/1.1	[39]
	T3	COF-366	23.1	5.6	1252/1146	16.3/0.7	29/1.3	0.3/1.1	[40]
	T4	$[HO]_{50\%}$-TAPH-COF	23.1	3.9	1272/1166	16.4/0.5	42.1/1.4	0.3/1.1	[41]
	T5	Por-sp^{2}c-COF	23.6	4.2	1340/1226	17.6/0.6	41.9/1.4	0.4/1.2	[42]
tetragonal	T6	Pc-PBBA-2R COF	25.0	3.3	1188/1486	38.6/1.5	115.7/4.4	0.2/1.1	
	T7	COF-66	27.2	3.8	1480/1354	22.2/0.2	58.4/0.6	0.4/1.1	[40]
	T8	H_2P-Bph-COF	27.5	3.6	1472/1346	15.7/0.4	43.6/1	0.3/1.2	[65]
	T9	Pc-PBBA-3R COF	29.3	3.3	1408/1286	33.1/0.6	99/1.8	0.2/1.1	
	T10	Pc-PBBA-4R COF	33.6	3.3	1628/1086	28.9/0.4	86.4/1.1	0.2/1.1	
					along edge/ short diagonal /long diagonal	along edge/ short diagonal/long diagonal	along edge/short diagonal/long diagonal	along edge/short diagonal /long diagonal	
	R1	Py-TPE-COF	14.6	4.6	702/654/634	26.3/1.8/3.3	57.6/3.8/7.3	0.4/0.7/1.8	[66]
	R2	TTF-Py-COF	15.2	3.7	750/682/694	13.3/1.6/3.3	36/4.3/8.9	0.5/0.9/1.2	[43]
rhombic	R3	Cz-COF1	16.9	4.0	820/750/750	22.1/1.3/3.6	55.5/3.3/9.1	0.3/0.5/1.8	[44]
	R4	COF-ETBA-DAB	17.0	4.6	868/794/794	30.5/0.5/0.7	66.4/1/1.5	0.1/0.7/1.6	[67]
	R5	Py-	18.5	3.9	741/814/	11.1/0.7/1.7	28.4/1.8/4.	0.4/0.7/1	[45]

		Azine COF			814		4	.7	
	R6	Cz-COF2	20.7	4.0	1040/950/950	10.3/0.7/0.6	25.8/1.6/1.6	0/1/1.2	44
	R7	LZU-192/TPPy-BOP	21.6	3.5	1064/974/974	24.7/0.7/1.1	70.3/1.9/3	0.7/0.7/2	13,57
	R8	ILCOF-1	21.8	7.9	1108/1014/1014	21.6/0.7/0.9	27.4/0.9/1.1	0.3/1.5/0.9	46
	R9	[HO]$_{50\%}$-Py-COF	22.0	3.8	1132/1034/1034	24.8/0.6/0.7	64.9/1.6/1.9	0.2/1.2/1	47
	R10	Py-COF	22.0	3.9	1108/1014/1014	16.9/0.3/0.5	43.5/0.9/1.4	0.2/1.3/0.9	48
	R11	sp^{2}c-COF	22.2	3.8	1196/1094/1094	9/0.7/1.5	23.9/1.9/4	0.4/0.7/2.1	49
	R12	TTF-COF	22.2	4.3	964/882/882	12.2/0.6/0.6	28.4/1.4/1.5	0.7/0.7/1.7	50
	R13	sp^{2}c-COF-2	26.4	3.7	1416/1294/1294	17.2/0.5/0.6	46.2/1.3/1.5	0.5/0.7/2.1	49
	R14	sp^{2}c-COF-3	31.0	3.7	1635/1494/1494	17.1/0.4/0.4	46.5/1/1.1	0.5/0.7/2.2	49
					along edge/ diagonal	along edge/ diagonal	along edge/ diagonal	along edge/ diagonal	
	S1	COF-BTA-DAB	20.0	3.6	930/1110	12.3/10.3	34.5/28.8	0.8/0.6	51
	S2	COF-BTA-BZ	27.5	3.6	1290/1530	10.1/7.7	27.9/21.3	0.8/0.7	51
star-pore	S3	TPE-Ph COF	28.1	4.5	1322/1594	19.8/8.7	44/19.3	0.7/0.7	52
	S4	ETTA-TPA COF/Dual-Pore COF	29.4	4.8	1386/1662	13/7.1	27.2/14.8	0.6/0.6	53
	S5	NUS-30	37.9	4.7	1786/2162	8.1/4.8	17.3/10.3	0.6/0.9	54

Overall, the values obtained by DFTB agree well with previous calculations: the Young's moduli of COF-5 (H28, pore width 27.7 Å) calculated by DFTB are 22.1 GPa and 14.5 GPa in the zigzag and armchair directions, respectively; these are comparable to those obtained using the OPLS-AA force field (24.2 GPa and 15.1 GPa, respectively)[68] and DFT calculations with the Perdew–Burke–Ernzerh functional (15.5 GPa and 15.3 GPa, respectively).[69] The Young's moduli of TP-COF (H34, pore width 32.5 Å) and COF-1 (H4, pore width 12.5 Å) in the armchair direction determined by DFTB are 5.3 GPa and 26.6 GPa, respectively; these results are close to those calculated using the AIREBO classical force field (5 GPa and 30 GPa, respectively);[70] ReaxFF

leads to a Young's modulus of 20.7 GPa in the armchair direction for COF-1.[71]

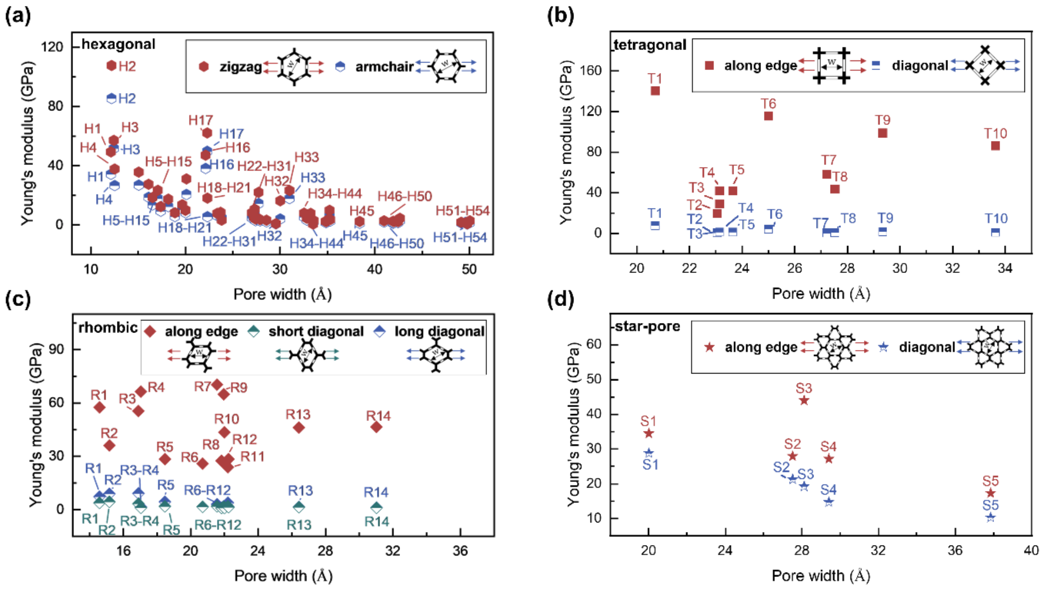

Figure S14. Young's moduli as a function of the pore width for 2D COFs with (a) hexagonal, (b) tetragonal, (c) rhombic, and (d) star-pore topologies, assessed within a strain range of <5%. Insets depict the stretching directions and the definition of pore width (*W*).

For hexagonal 2D COFs (H1–H57), our calculations reveal that Young's moduli span from 0.5 GPa to 107.8 GPa and decrease with an increase in pore width (**Figure S14a**). This decrease can also be understood by the relationship between the elastic modulus and the pore edge length $E \propto (1/l)^3$, as the in-plane Young's modulus is proportional to the elastic modulus for 2D COFs. Notably, Young's moduli are marginally higher in the zigzag direction compared to the armchair direction. This feature reflects the detailed impact of the irregular molecular structures at the atomic level, leading to slight deviations from the relationships governing macroscopic objects. Among the hexagonal 2D COFs analyzed, COF-6 (H2; pore width = 12.2 Å and density = 1.1 g/cm^3)[72] exhibits the highest Young's modulus of 107.8 GPa in the zigzag direction. By contrast, LUZ-1-3R (H53; pore width = 49.8 Å) exhibits the lowest Young's modulus among the studied hexagonal 2D COFs, calculated to be 0.8 GPa in the zigzag direction and 0.6 GPa in the armchair direction.

Young's moduli of tetragonal and rhombic 2D COFs decrease with an increase in pore width and exhibit substantial anisotropy (**Figures S14b** and **14c**). Specifically, Young's moduli are substantially higher along the edges of the pores compared to the diagonal direction. For example, Young's modulus of the Pc-PBBA COF (T1, pore width = 20.1 Å) is 140.4 GPa along the edge, whereas it is 1.5 GPa in the diagonal direction. In the case of rhombic 2D COFs, the maximum Young's modulus along the pore edge is 70.3 GPa (LZU-192, also referred to as TPPy-BOP, R7; pore width = 21.6 Å). Additionally, Young's moduli are slightly higher when the material is stretched along the long diagonal direction compared to the short diagonal direction.

Young's moduli of star-pore 2D COFs (S1–S5) exhibit varying degrees of anisotropy (**Figure S14d**). Specifically, Young's moduli when stretched along the edge are consistently higher than those along the diagonal direction. The most pronounced difference is observed in the TPE-Ph COF (S3, pore width = 28.1 Å), where Young's moduli along the edge and diagonal direction are 44.0 GPa and 19.3 GPa, respectively.

The further calculations indicate that the trend of decreasing modulus with increasing pore size is also applicable to 2D COFs with other chemical linkages, such as those containing alkyne groups, as shown in **Tables S1**, **S2**, and **S3**.

Further analysis reveals that the anisotropic behavior of Young's moduli in the 2D COFs is associated with the changes in pore shapes during mechanical stretching. A direct correlation exists between minimal anisotropy observed in Young's moduli and the disparity in pore shape alterations along various directions during mechanical stretching (**Figure S15**).

5. Pore shapes during stretching

For hexagonal 2D COFs, the degrees of angle changes within the pores are similar when stretched in different directions. For example, COF-5 (H28) shows an internal angle change of 8.1% and 7.8% at 5% strain in the zigzag and armchair directions, respectively (**Figure S15a**). This aligns with the small anisotropy in their Young's moduli.

For tetragonal and rhombic 2D COFs, the pore angles remain unchanged when stretched along the edge (**Figures S15b** and **15c**), indicating that the strain primarily originates from the elongation of chemical bonds. This explains the high Young's moduli in this direction. Conversely, in the diagonal directions, the internal angles change by 5.8%-7.2% at 5% strain, corresponding to the more flexible torsional angles.

For star-pore 2D COFs, the degrees of internal angle changes are comparable in different stretching directions. For example, in ETTA-TPA COF (S4), the angle changes by 3.3% at 5% strain along the edge, compared to that of 4.1% in the diagonal direction, as depicted in **Figure S15d**. This corresponds to their moderate degree of anisotropy in Young's moduli.

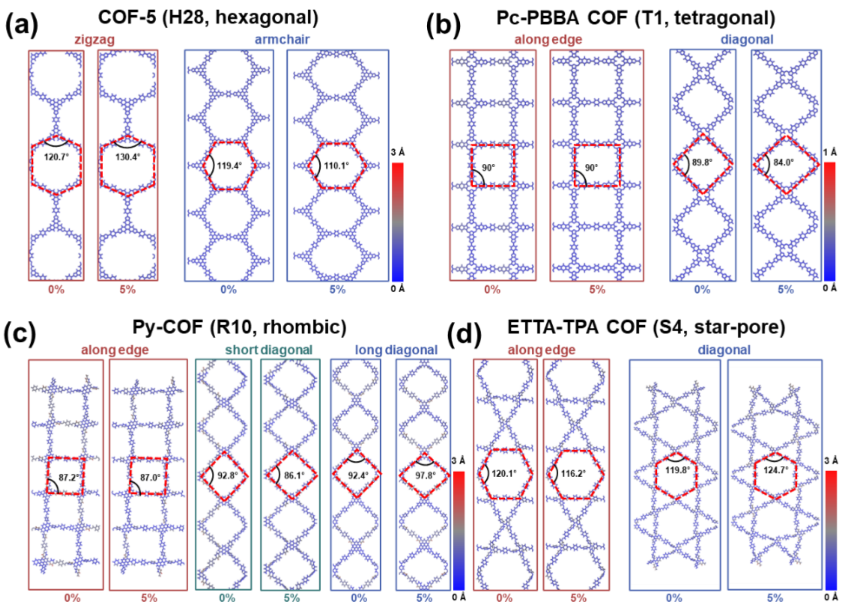

Figure S15. The structure of 2D COF at 0% and 5% strains: (a) COF-5 (H28, hexagonal), (b) Pc-PBBA COF (T1, tetragonal), (c) Py-COF (R10, rhombic), (d) ETTA-TPA COF (S4, star-pore).

6. The internal angles of rhombic 2D COFs

Table S4. The internal angles *θ* during stretching rhombic 2D COFs at 0% strain.

linkage type	label	name	short diagonal	long diagonal	short diagonal average	long diagonal average
imine	R1	Py-TPE-COF	93.4°	85.1°	96.7°	82.2°
	R2	TTF-Py-COF	104.7°	73.2°		
	R3	Cz-COF1	99.4°	80.6°		
	R4	COF-ETBA-DAB	99.4°	80.5°		
	R5	Py-Azine COF	92.4°	87.2°		
	R6	Cz-COF2	93.8°	86.4°		
	R8	IL-COF-1	89.5°	86.2°		
	R9	$[HO]_{50\%}$-Py-COFs	92.3°	85.7°		
	R10	Py-COF	92.8°	87.6°		
	R12	TTF-Ph-COF	109.1°	69.7°		
cyanoethylene	R11	sp^2c-COF	92.8°	87.6°	95.0°	81.6°
	R13	sp^2c-COF-2	95.6°	79.6°		
	R14	sp^2c-COF-3	96.6°	77.6°		
oxazole bond	R7	LZU-192/TPPy-BOP	94.9°	83.1°		

7. The derivation of the elastic modulus of a rhombic network

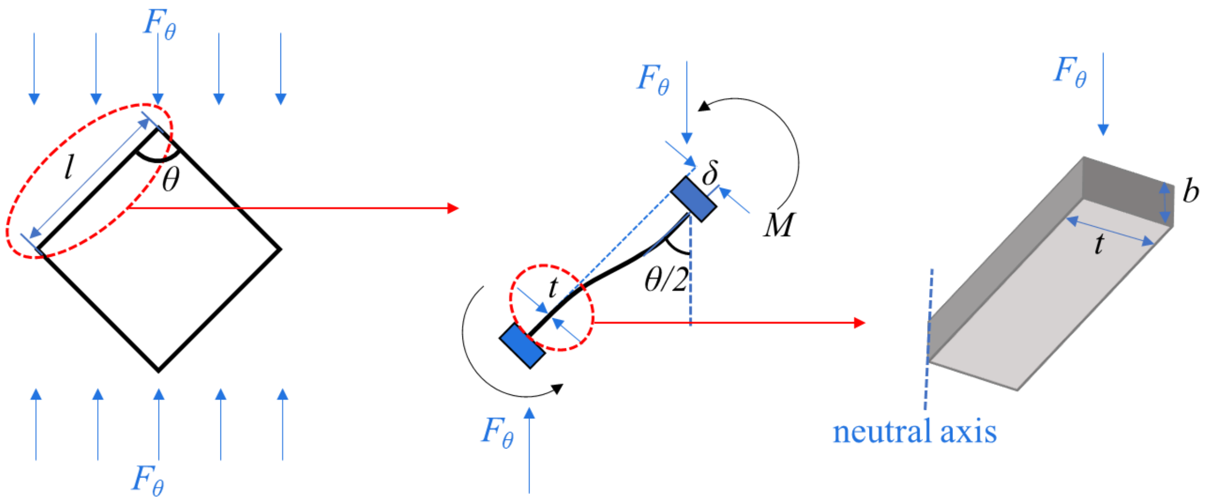

Figure S16. Stress analysis of a rhombic network under compression.

The force (F_θ) is expressed as:

$$F_\theta = \sin(\theta/2)\sigma_\theta bl \tag{S1}$$

where b represents the thickness of network edge, l represents the length of the network edge.

The bending moment (M) is calculated to be:

$$M = \frac{1}{2} F_\theta l \sin(\theta/2) \deg \tag{S2}$$

According to the standard beam theory, the deformation quantity (δ) in the vertical beam direction is：

$$\delta = \frac{Ml^2}{6E_s I} \tag{S3}$$

where Es represents possessing fully dense properties of Young's modulus, the moment of inertia (I) is (the classic moment of inertia expression for circular and tetragonal tubes):

$$I = \frac{bt^3}{12} \tag{S4}$$

where t represents the width of the network edge.

Under compression, the strain in the direction of stress can be expressed as:

$$\varepsilon = \frac{\delta \sin(\theta/2)\deg}{l\cos(\theta/2)\deg} = \frac{\sin(\theta/2)^3 \sigma_\theta b l^3}{12\cos(\theta/2) E_s I} = \frac{\sin(\theta/2)^3 \sigma_\theta l^3}{\cos(\theta/2) E_s t^3} \quad \text{(S5)}$$

The elastic modulus of a rhombic network writes:

$$E = \frac{\sigma_\theta}{\varepsilon} = \frac{\cos(\theta/2) E_s t^3}{\sin(\theta/2)^3 l^3} \quad \text{(S6)}$$

The proportionality coefficient is expressed as:

$$\frac{E}{E_s} = \frac{\cos(\theta/2) t^3}{\sin(\theta/2)^3 l^3} \quad \text{(S7)}$$

8. The elastic moduli and Poisson's ratios of 2D COFs of simulation/measurement from literature

Table S5. The elastic modulus of 2D COFs by simulation/measurement from literature.

2D COFs	Young's modulus/experiment (GPa)	thickness (Å)	number of layers	elastic modulus /experiment (N/m)	predicted elastic moduli (N/m)	Ref
$COF_{TTA-DHTA}$	25.90	3.4	14	8.81	1.03	[73]
TAPB-BTCA-MCOF	0.80	3.4	-	0.27	25.14	[74]
PPDA-BTCA-MCOF	0.50	3.4	-	0.17	8.11	[74]
TAPB-PDA COF	0.40	3.5	-	0.14	1.01	[74]
TAPB-PDA COF	1.45	3.5	257	0.51	1.01	[75]
TAPB-PDA COF	6.80	3.5	29	2.38	1.01	[76]
TAPB-PDA COF	44.60	3.6	1	16.19	1.01	[77]
TAPB-DMTP COF	46.30	3.4	1	15.93	1.01	[77]
TAPB-DHTA COF	10.38	3.4	12-442	3.53	1.00	[63]

2DCOF-1	56.70	4.0	110	22.40	13.69	78
2DCOF-2	73.40	3.1	142	22.68	11.63	78

2D COFs	Young's modulus /simulation (GPa)	thick ness (Å)	number of layers	elastic modulus /simulation (N/m)	predicted elastic moduli (N/m)	Ref
COF-1 (armchair)	20.70	3.4	1	7.00	11.01	71
COF-1 (zigzag)	33.98	3.4	1	11.49	11.01	71
COF-1 (armchair)	30.00	3.4	10	10.14	11.01	70
COF-1 (zigzag)	61.00	3.4	10	20.62	11.01	70
COF-1	28.50	3.4	1	9.63	11.01	69
BP-COF (armchair)	9.00	3.4	10	3.06	3.27	70
COF-5 (zigzag)	24.21	3.4	1	8.23	4.91	68
COF-5 (armchair)	15.13	3.4	1	5.14	4.91	68
COF-5 (armchair)	11.00	3.4	10	3.74	4.91	70
COF-5	15.40	3.4	1	5.24	4.91	69
TP-COF (armchair)	5.00	3.4	10	1.69	2.55	70
TAPB-PDA COF (zigzag)	7.63	3.5	1	2.67	1.01	68
TAPB-PDA COF (armchair)	4.00	3.5	1	1.40	1.01	68
TAPB-DHTA COF (zigzag)	14.00	3.4	4	4.76	1.00	63
TAPB-DHTA COF (armchair)	9.90	3.4	4	3.37	1.00	63

Table S6. The Poisson's ratios of 2D COFs by simulation/measurement from literature.

2D COFs	Poisson's ratio/experiment	Poisson's ratio /simulation	predicted Poisson's ratio	Ref
$COF_{TTA-DHTA}$	0.3	-	1	73
TAPB-DMTP COF	0.3	-	1	77
TAPB-PDA COF	0.3	-	1	77
COF-1 (armchair)	-	1	1	70
COF-1 (armchair)	-	0.836	1	71
COF-1 (zigzag)	-	0.38	1	71

COF-1-2R (armchair)	-	1.2	1	70
TP-COF (armchair)	-	1.6	1	70
COF-5 (armchair)	-	1.15	1	70
COF-5 (armchair)	-	0.9	1	68
COF-5 (zigzag)	-	0.9	1	68
COF-5 (zigzag)	-	1.2	1	70
DTPA	-	0.34	1	79
TAPB-PDA COF (armchair)	-	1	1	68
TAPB-PDA COF (zigzag)	-	1.1	1	68

9. Calculated E_st^x for hexagonal, tetragonal, rhombic, and star-pore 2D COFs

Table S7. Calculated E_st^x for hexagonal, tetragonal, rhombic, and star-pore 2D COFs with various types of chemical linkages[a].

topology	direction	coefficient	ether	boronate ester	oxazole	triazine
hexagonal	zigzag	E_st^3 (10^{-30} N·m^2)	23173	10498	4993	4547
	armchair	E_st^3 (10^{-30} N·m^2)	18581	8126	3551	3119
tetragonal	along edge	E_st (10^{-10} N)	-	1063	-	-
	diagonal	E_st^3(10^{-30} N·m^2)	-	14762	-	-
rhombic[b]	along edge	E_st (10^{-10} N)	-	-	-	-
	short diagonal	E_st^3 (10^{-30} N·m^2)	-	-	-	-
	long diagonal	E_st^3 (10^{-30} N·m^2)	-	-	-	-
star-pore	along edge	E_st (10^{-10} N)	-	-	-	-
	diagonal	E_st (10^{-10} N)	-	-	-	-
			imine	boroxine	imide	cyanoethylene
hexagonal	zigzag	E_st^3 (10^{-30} N·m^2)	4026	3545	3085	-
	armchair	E_st^3 (10^{-30} N·m^2)	2742	2557	2260	-
tetragonal	along edge	E_st (10^{-10} N)	418	-	-	-
	diagonal	E_st^3 (10^{-30} N·m^2)	5178	-	-	-
rhombic[b]	along edge	E_st (10^{-10} N)	387	-	-	390
	short diagonal	E_st^3 (10^{-30} N·m^2)	4327	-	-	6611

	long diagonal	E_st^3 (10^{-30} N·m^2)	5094	-	-	6972
star-pore	along edge	E_st (10^{-10} N)	357	-	-	-
	diagonal	E_st (10^{-10} N)	256	-	-	-

[a]Azine and hydrazone chemical linkages have too few instances for fitting functions and thus are not included.

[b]The average angles θ at 0% strain (used to calculate the coefficients) when stretched in the short diagonal direction with imine and cyanoethylene chemical linkages are 96.7° and 95.0°, respectively. When stretched in the long diagonal direction, these values are 82.2° and 81.6°, respectively. Details are shown in **Table S4**.

10. The changes in bond lengths, angles, and dihedral angles for hexagonal 2D COFs in the armchair and zigzag directions

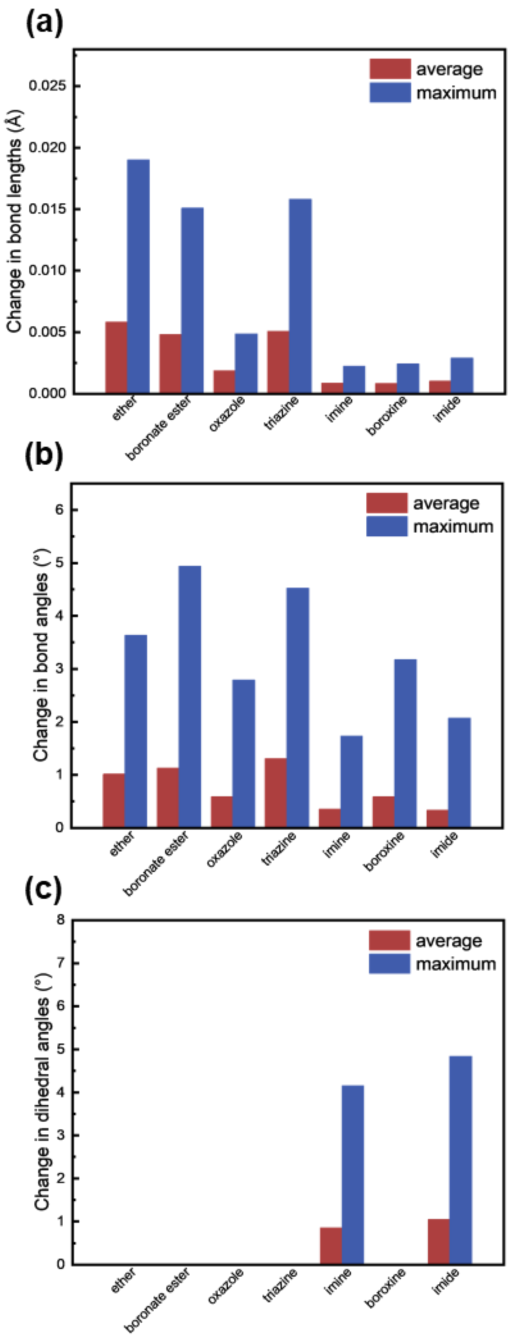

Figure S17. The changes in bond lengths, bond angles, and dihedral angles for hexagonal 2D COFs with different chemical linkages when stretched in the armchair direction at 5% strain: ether (H33, JUC-506), boronate ester (H28, COF-5), oxazole (H36, TPB-BOP), triazine (H1, CTF-1), imine (H40, TAPB-DHTA COF), boroxine (H26, COF-1-3R), and imide (H38, COF-PI2).

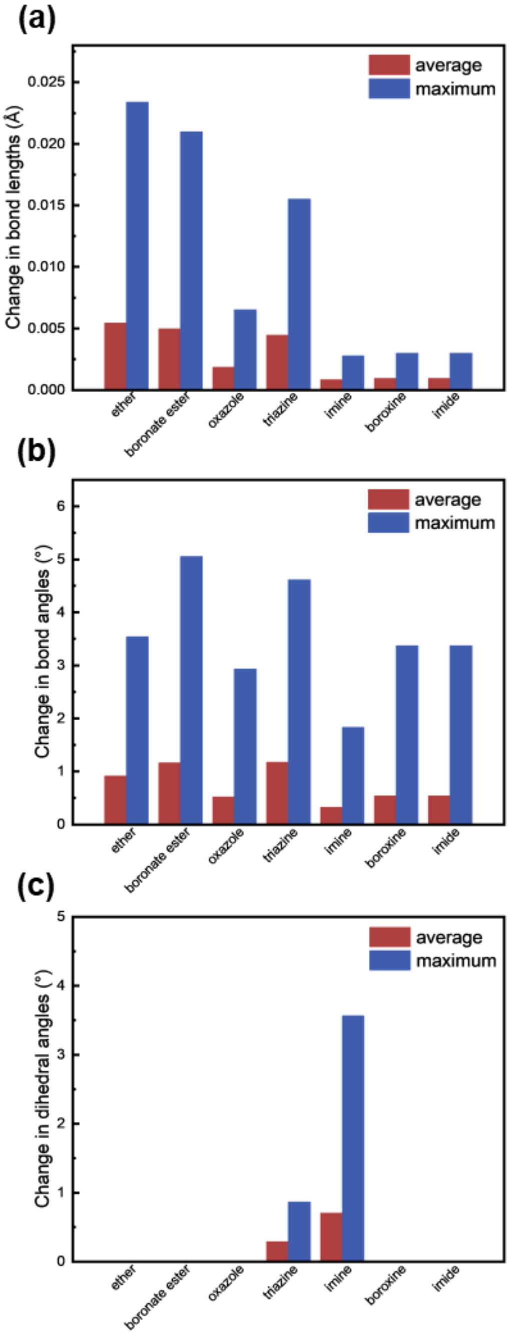

Figure S18. The changes in bond lengths, bond angles, and dihedral angles for hexagonal 2D COFs with different chemical linkages when stretched in the zigzag direction at 5% strain: ether (H33, JUC-506), boronate ester (H28, COF-5), oxazole (H36, TPB-BOP), triazine (H1, CTF-1), imine (H40, TAPB-DHTA COF), boroxine (H26, COF-1-3R), and imide (H38, COF-PI2).

11. Poisson's ratios of 2D COFs retain the characteristics of their corresponding macroscopic networks

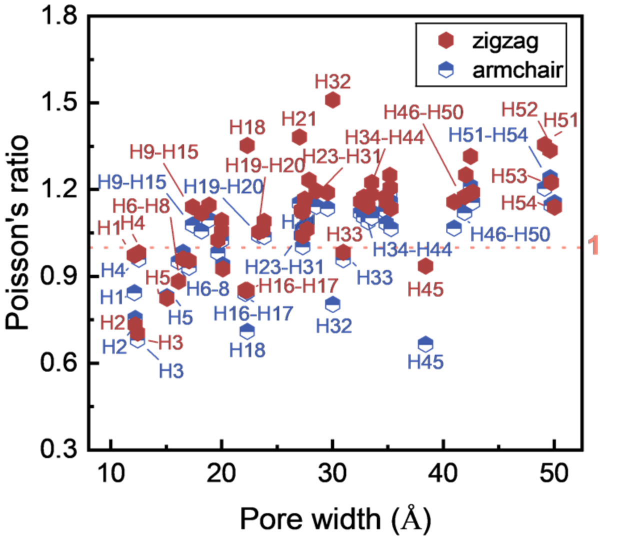

Figure S19. Calculated Poisson's ratios of hexagonal 2D COFs. The dashed line represents Poisson's ratios of the macroscopic hexagonal network.

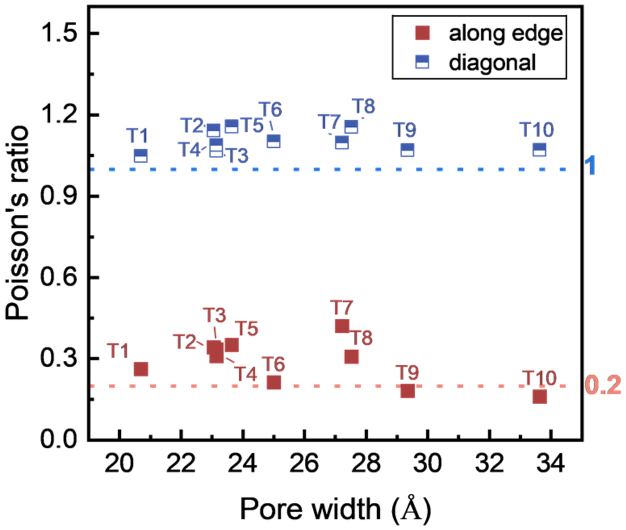

Figure S20. Calculated Poisson's ratios of rhombic 2D COFs. The dashed lines represent Poisson's ratios of the macroscopic tetragonal network.

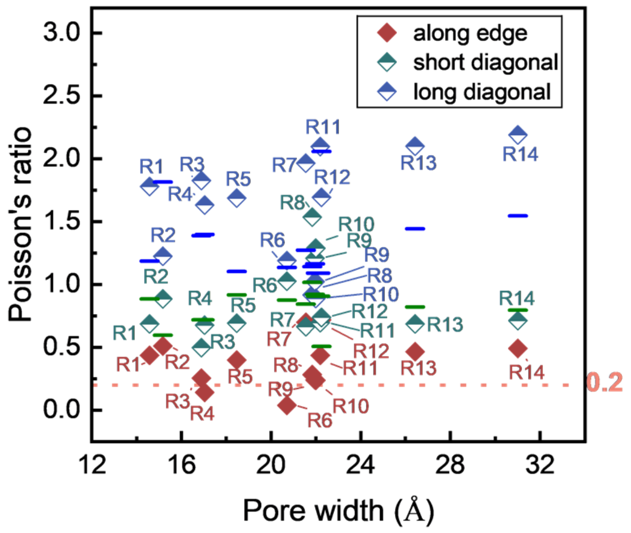

Figure S21. Calculated Poisson's ratios of tetragonal 2D COFs. The dashed lines represent Poisson's ratios of the macroscopic rhombic network.

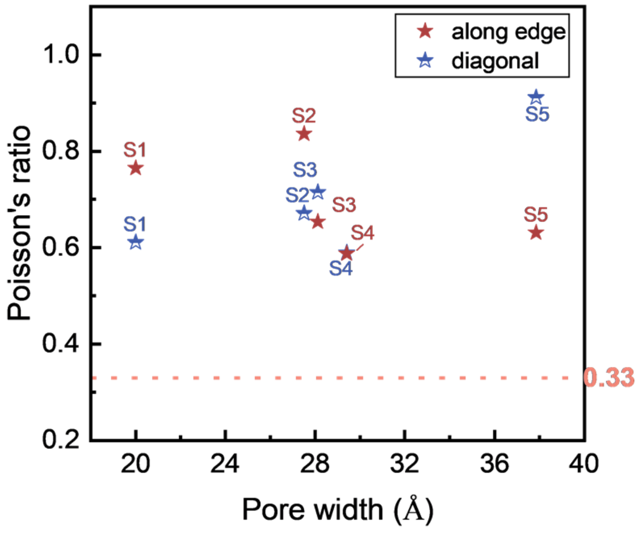

Figure S22. Calculated Poisson's ratios of star-pore 2D COFs. The dashed line represents Poisson's ratios of the macroscopic star-pore network.

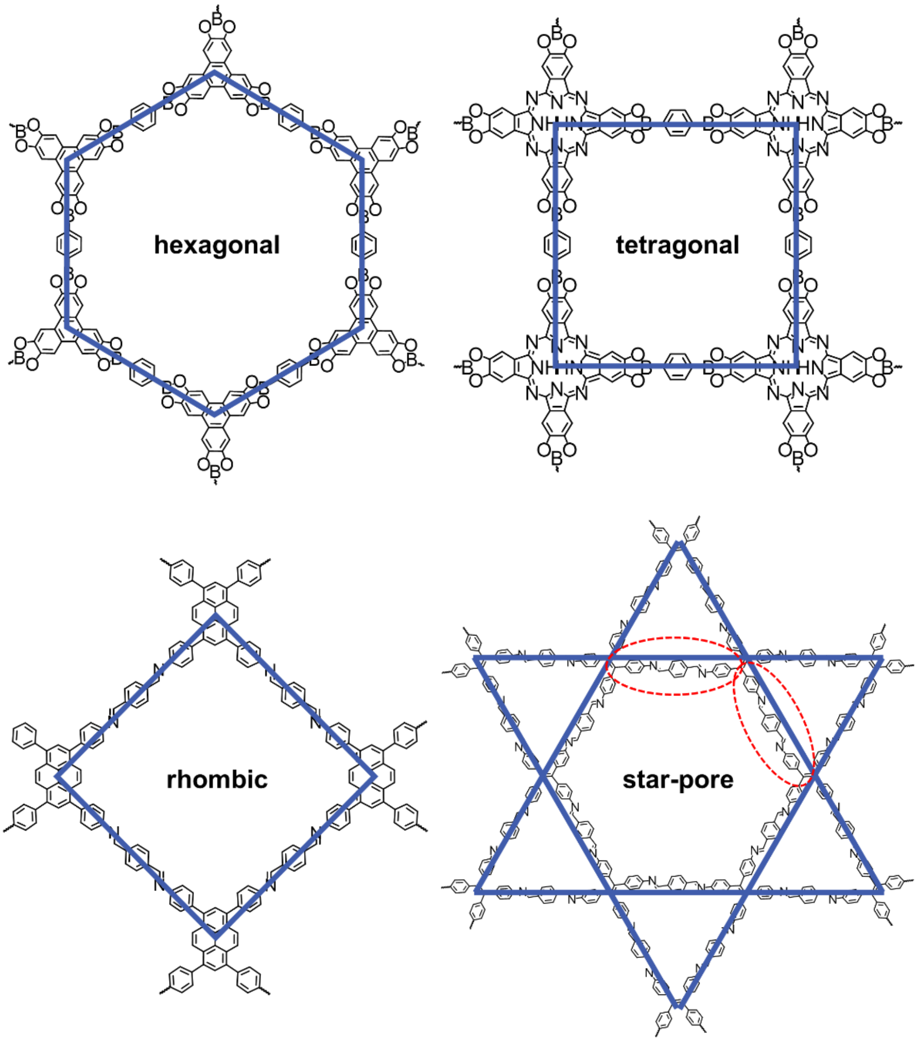

Figure S23. Comparison between the chemical structures of selected 2D COFs and their corresponding perfect macroscopic topologies (blue lines). Red dashed circles highlight the deviation from the star-pore topology.

12. Stresss–train curves of 2D COFs with hexagonal, tetragonal, rhombic, and star-pore topologies.

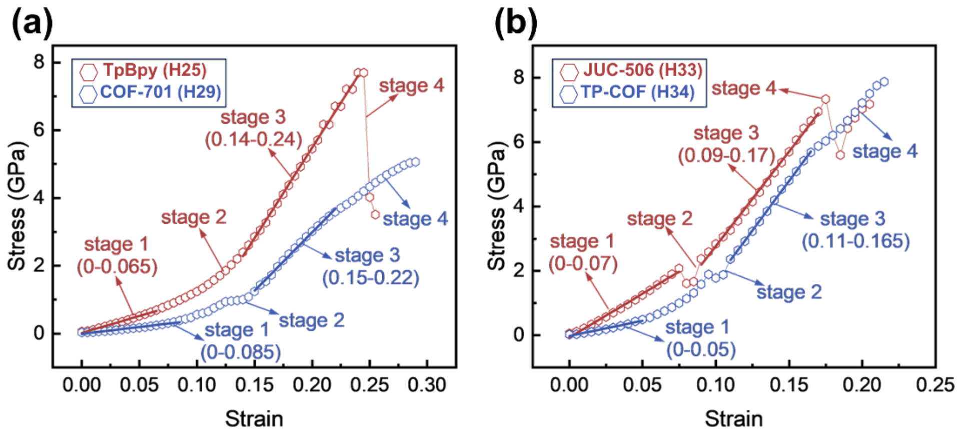

Figure S24. Stress–strain curves of hexagonal in the zigzag direction: (a) TpBpy (H25) and COF-701 (H29); (b) JUC-506 (H33) and TP-COF (H34).

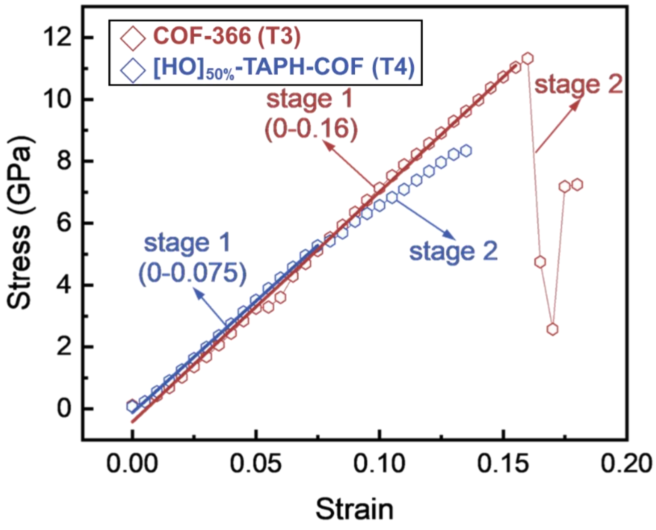

Figure S25. Stress–strain curves of tetragonal COF-366 (T3) and $[HO]_{50\%}$-TAPH-COF (T4) along the edge direction.

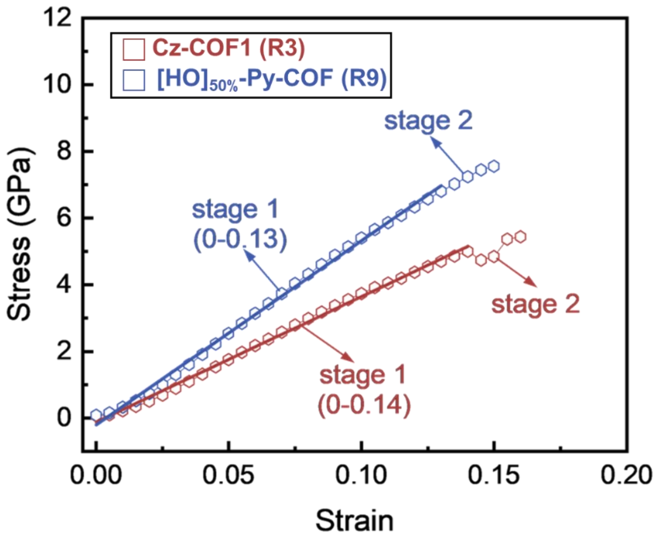

Figure S26. Stress–strain curves of rhombic Cz-COF1 (R3) and $[HO]_{50\%}$-Py-COF (R9) along the edge direction.

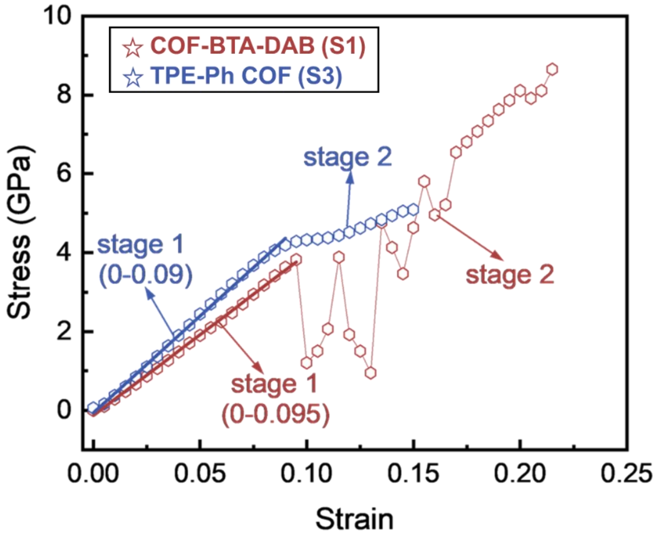

Figure S27. Stress–strain curves of star-pore COF-BTA-DAB (S1) and TPE-Ph COF (S3) along the edge direction.

13. Structures change under different strains for COF-5, Pc-PBBA COF, Py-COF, and ETTA-TPA COF

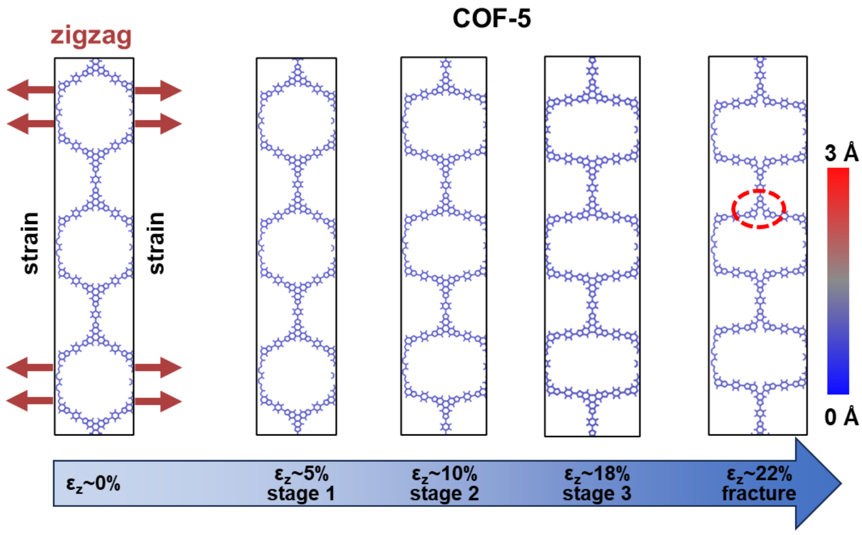

Figure S28. Structures change under strain in the zigzag direction for COF-5.

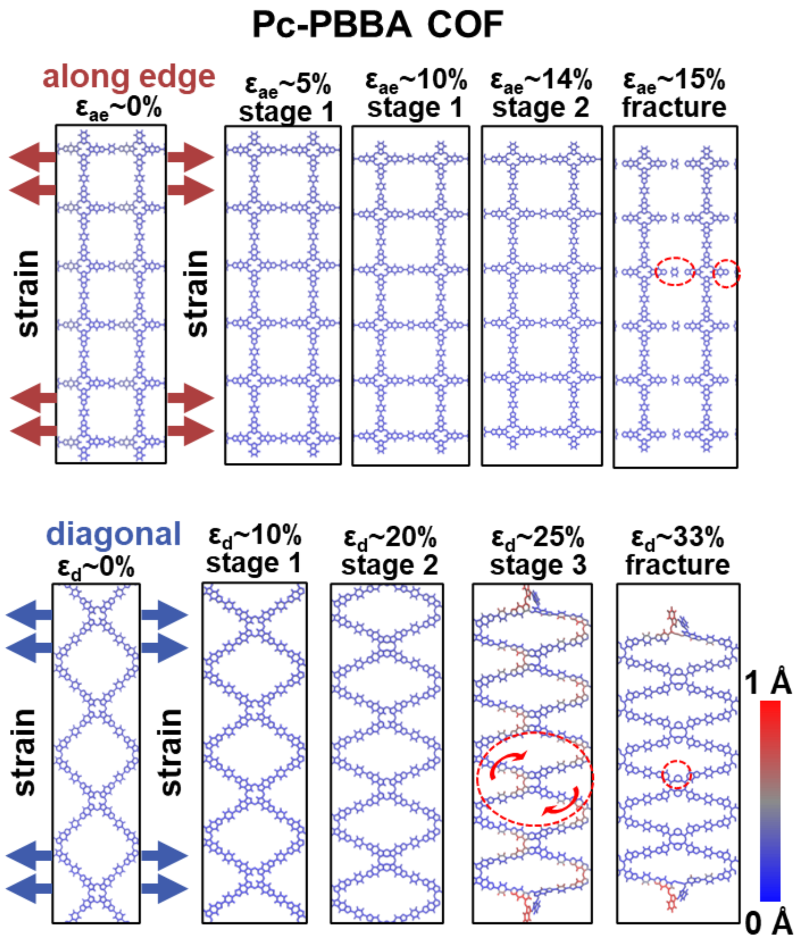

Figure S29. Structures change under different strains for Pc-PBBA COF.

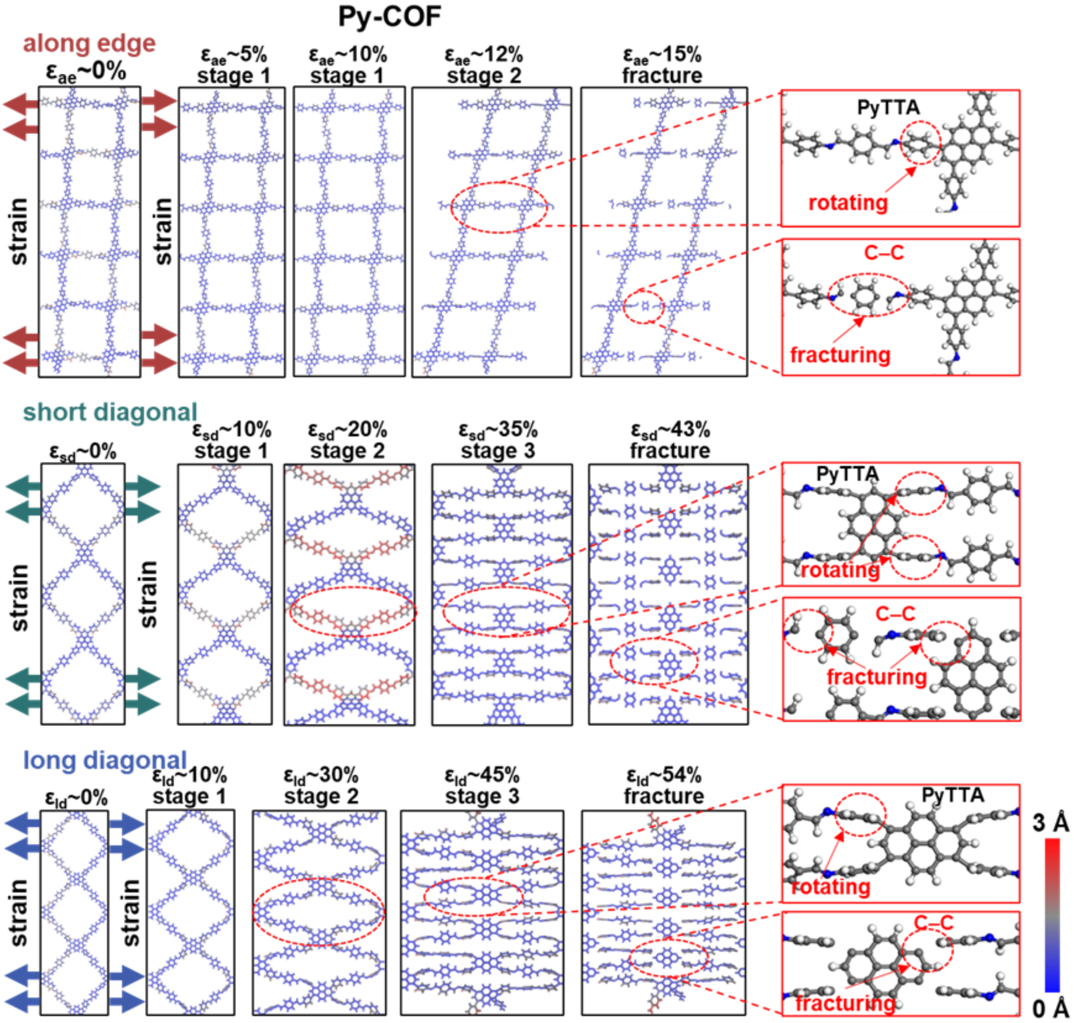

Figure S30. Structures change under different strains for Py-COF.

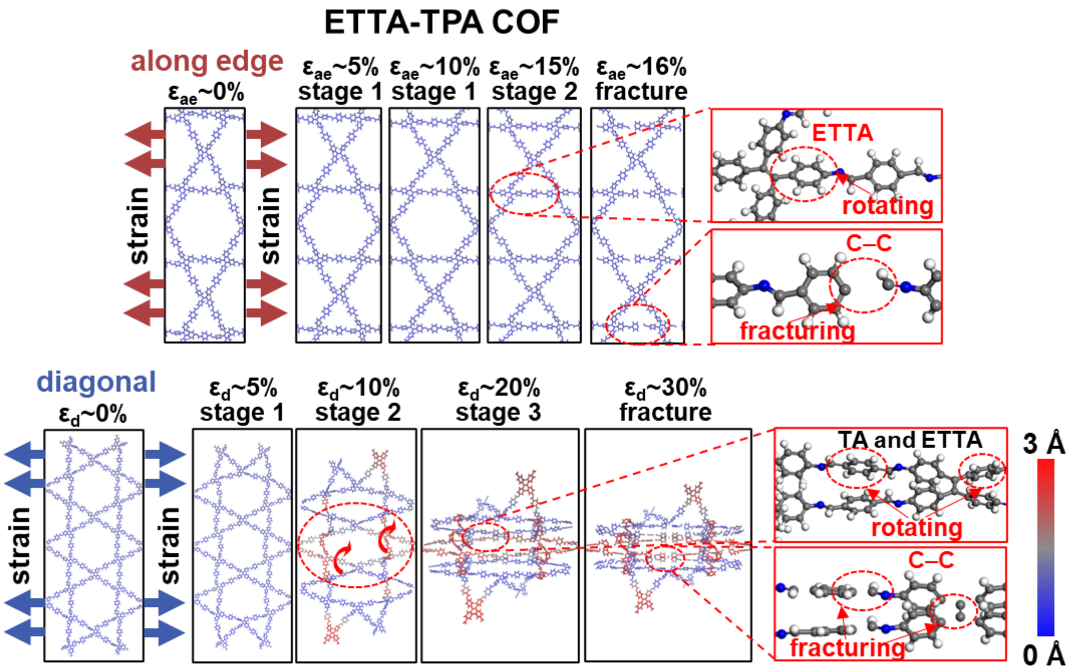

Figure S31. Structures change under different strains for ETTA-TPA COF.

14. The evolution of Poisson's ratios and pore sizes of 2D COFs during stretching

As shown in **Figure S32**, when stretched along the edge for Pc-PBBA COF, Py-COF, and ETTA-TPA COF, Poisson's ratios slightly decrease. In the other cases, Poisson's ratio initially increases with strain and then saturates or decreases. These features can also be related to the structural characteristics under tensile stress discussed above. Typically, angle changes lead to increased Poisson's ratios, while bond stretching slightly decreases their values. This point is illustrated by the increase in the Poisson's ratio of COF-5 at a strain value of approximately 10% strain (when benzene rings start to twist) in the armchair direction, and that for ETTA-TPA COF at 5%–15% strains (when molecular groups deform in the out-of-plane direction) in the diagonal direction. Further analysis shows that the dimension of the pores changes linearly with stress under low-to-medium strains (see **Figure S33**), suggesting that their molecular separation ability can be tuned through mechanical stress.

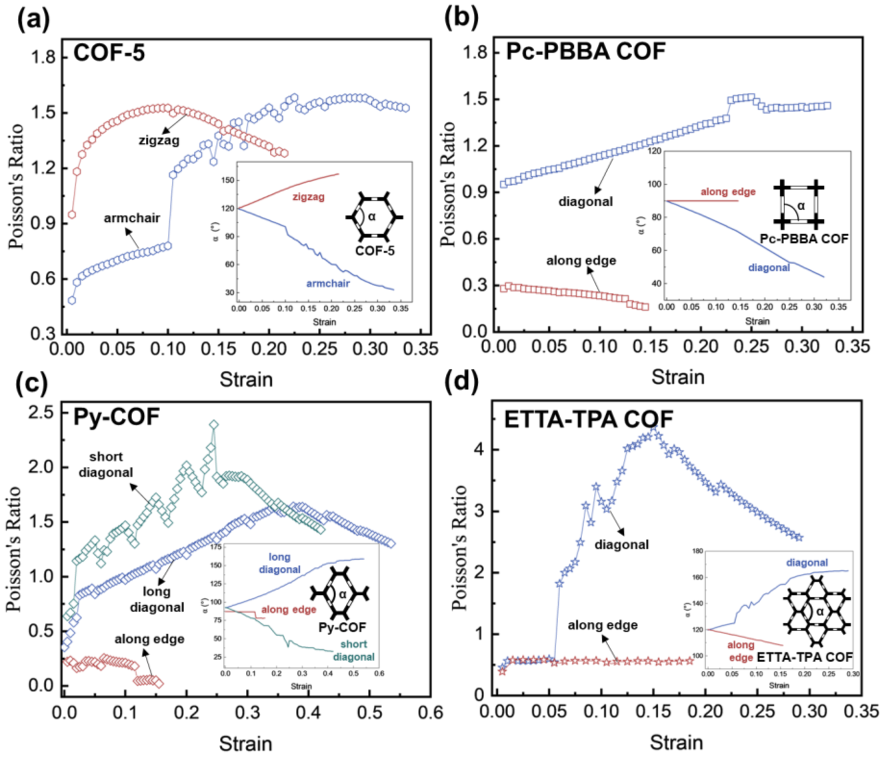

Figure S32. Poisson's ratios of (a) COF-5, (b) Pc-PBBA COF, (c) Py-COF, and (d) ETTA-TPA COF during stretching.

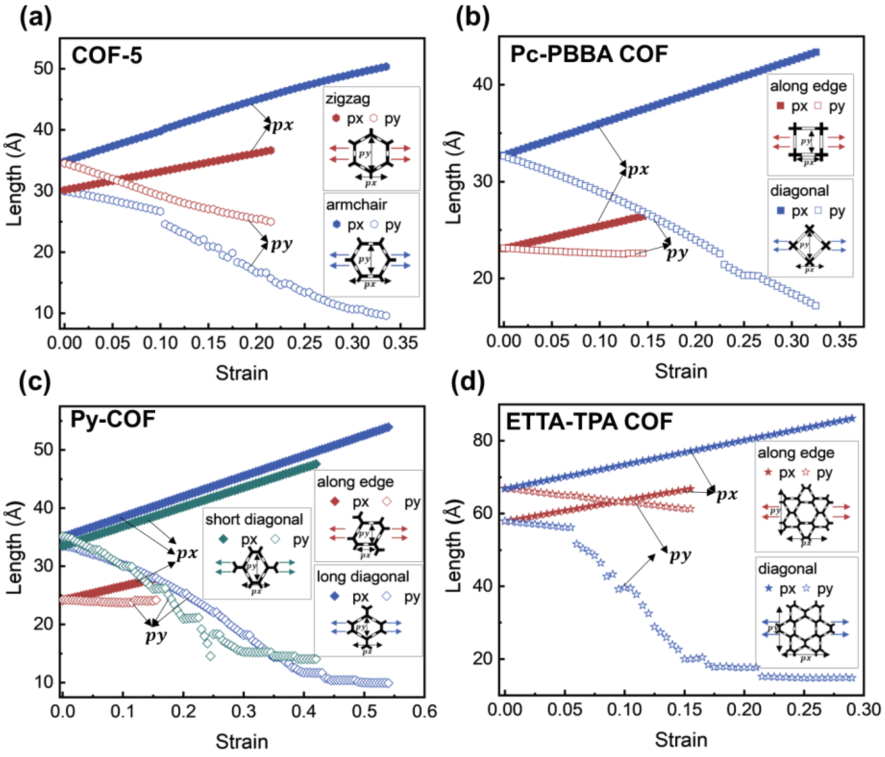

Figure S33. The pore dimensions of (a) COF-5, (b) Pc-PBBA COF, (c) Py-COF, and (d) ETTA-TPA COF during stretching.

15. Fracture strain, ultimate strength, fracture strength, and location of fracture for selected 2D COFs

Table S8. Fracture strain, ultimate strength, fracture strength, broken bonds, and location of fracture for selected 2D COFs.

material	direction	fracture strain	ultimate strength (GPa)	fracture strength (GPa)	broken bonds	location of fracture
COF-5	Zigzag	21.5%	9.8	9.8	C=C	HHTP (core)
	Armchair	33.5%	10.3	10.3	C–B	BDBA (linker)
Pc-PBBA COF	Along edge	14.5%	15.6	13.7	C–B	PBBA (linker)
	Diagonal	32.5%	8.2	8.2	C=C	Pc (core)
Py-COF	Along edge	14.5%	8.3	8.3	C–C	TA (linker)
	Short Diagonal	42%	10.3	10.3	C–C	TA (linker)
	Long diagonal	53%	9.9	9.9	C–C	PyTTA (core)
ETTA-TPA COF	Along edge	15.5%	4.5	4.5	C–C	TA (linker)
	Diagonal	29%	6.0	6.0	C–C	TA (linker)

16. The bond energies of C–B and C=C in COF-5

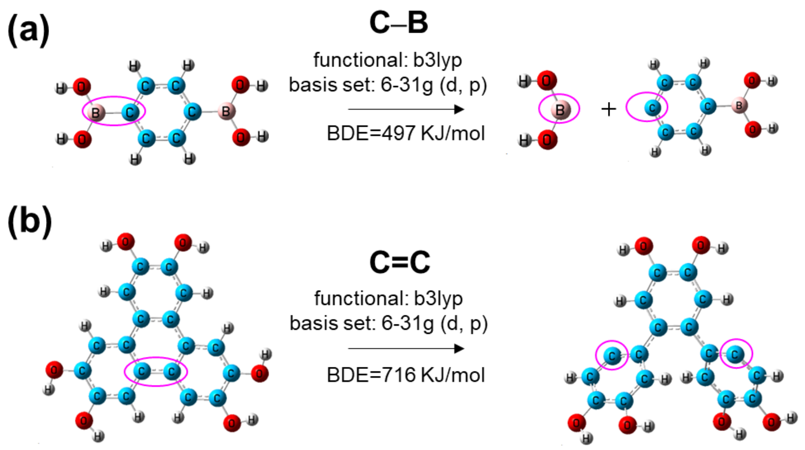

Figure S34. The bond energies of (a) C–B and (b) C=C in COF-5 determined by density functional energy calculations. Pink circles highlight the locations of bond breakage.

17. The locations of bond breakage for additional 2D COFs

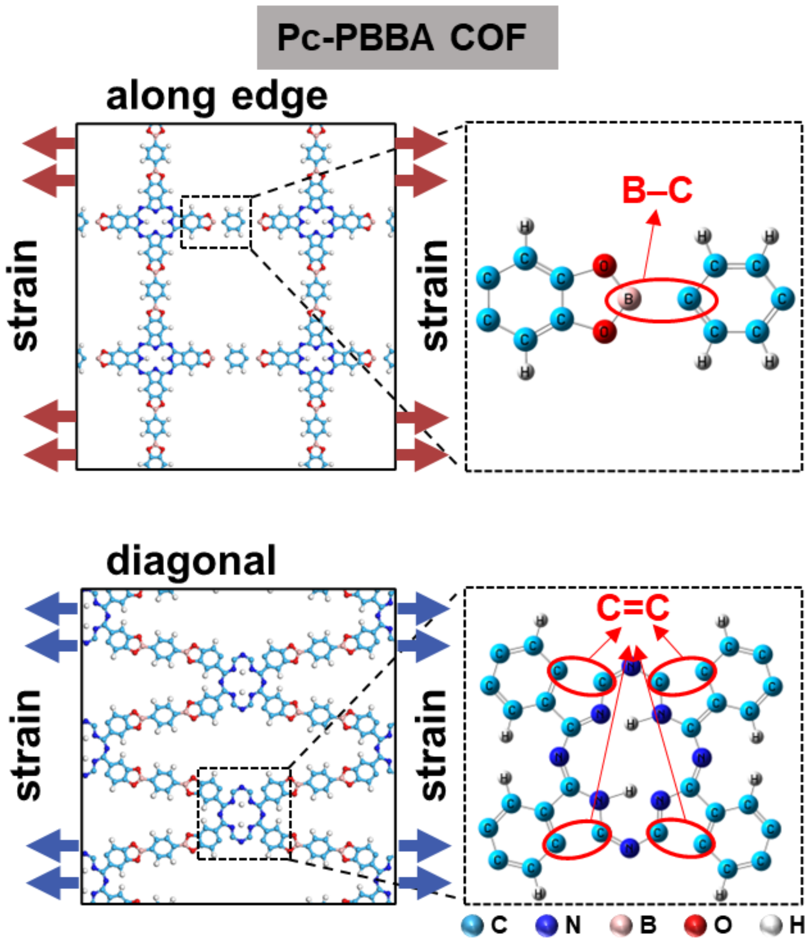

Figure S35. Illustrations of the locations of bond breakage for Pc-PBBA COF. Filled arrows show the stretching directions. Red circles show the locations of bond breakage.

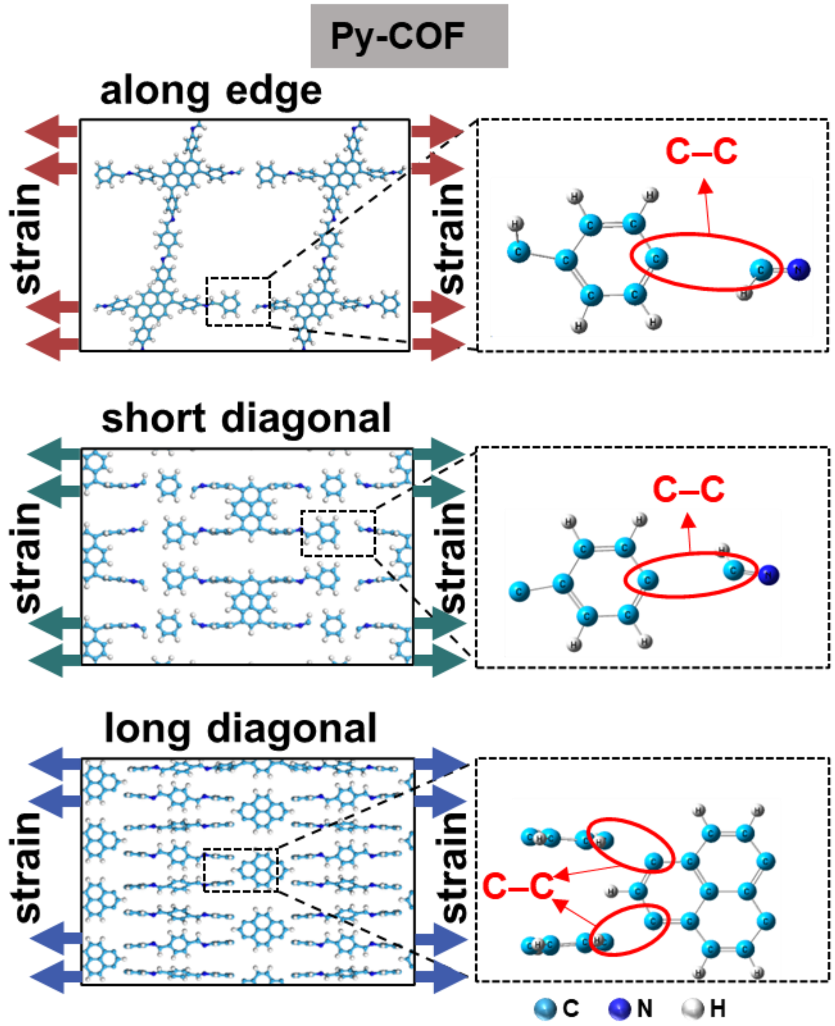

Figure S36. Illustrations of the locations of bond breakage for Py-COF. Filled arrows show the stretching directions. Red circles show the locations of bond breakage.

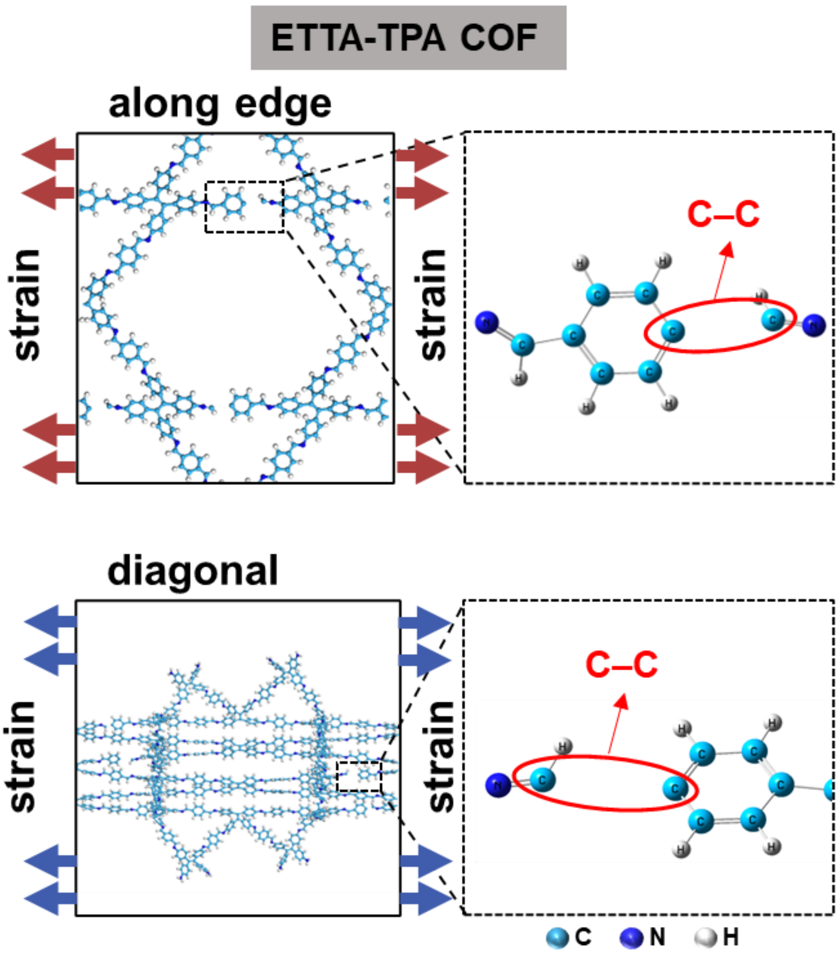

Figure S37. Illustrations of the locations of bond breakage for ETTA-TPA COF. Filled arrows show the stretching directions. Red circles show the locations of bond breakage.

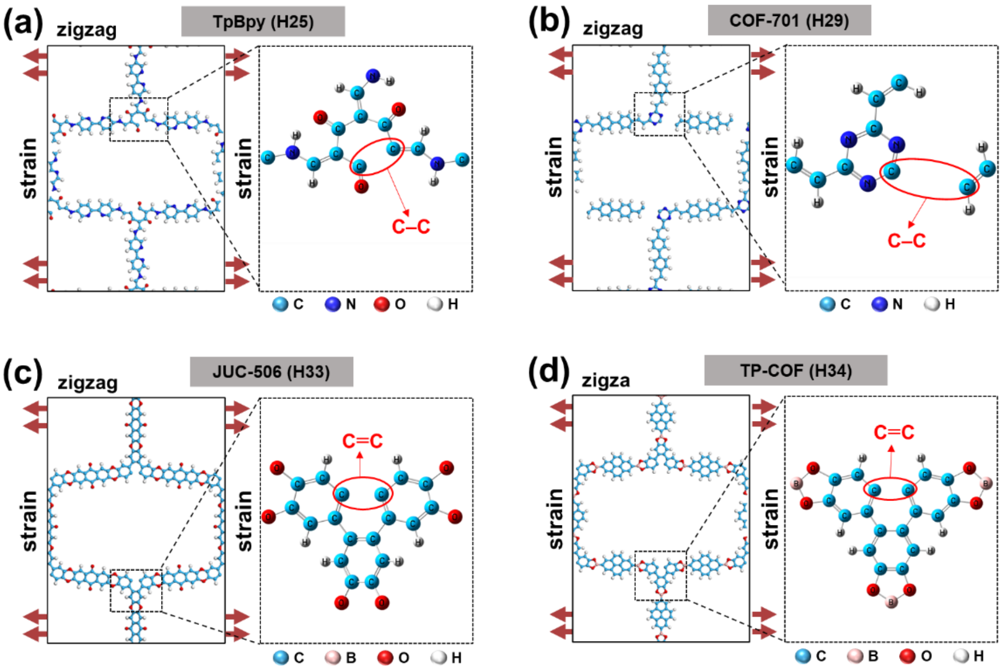

Figure S38. Illustrations of the locations of bond breakage for (a) TpBpy (H25), (b) COF-701 (H29), (c) JUC-506 (H33) and (d) TP-COF (H34) with hexagonal topology stretched in the zigzag direction. Filled arrows show the stretching directions. Red circles show the locations of bond breakage.

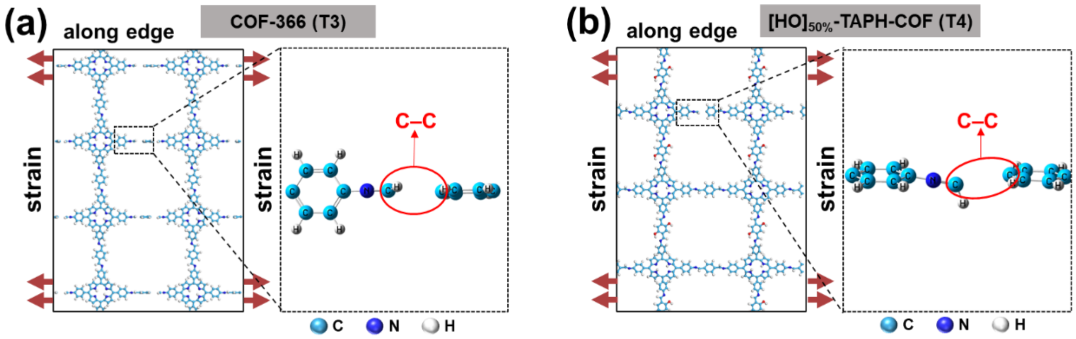

Figure S39. Illustrations of the locations of bond breakage for (a) COF-366 (T3) and (b) $[HO]_{50\%}$-TAPH-COF with tetragonal topology stretched along the edge direction. Filled arrows show the stretching directions. Red circles show the locations of bond breakage.

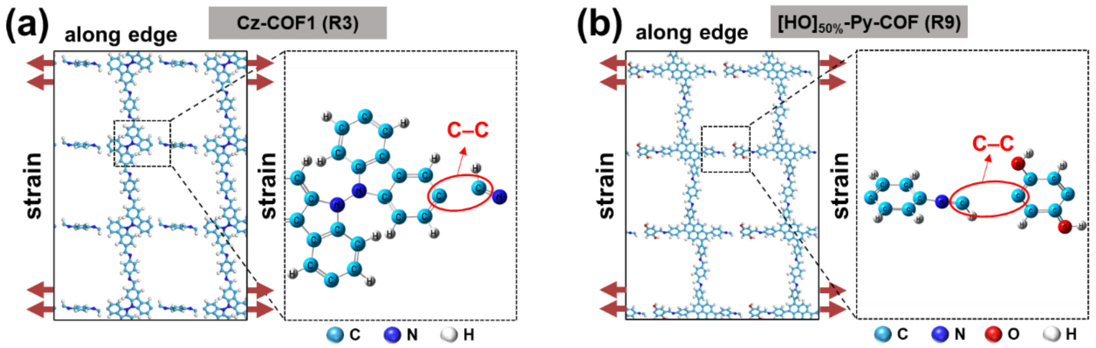

Figure S40. Illustrations of the locations of bond breakage for (a) Cz-COF1 (R3) and (b) [HO]50%-Py-COF (R9) with rhombic topology stretched along the edge direction. Filled arrows show the stretching directions. Red circles show the locations of bond breakage.

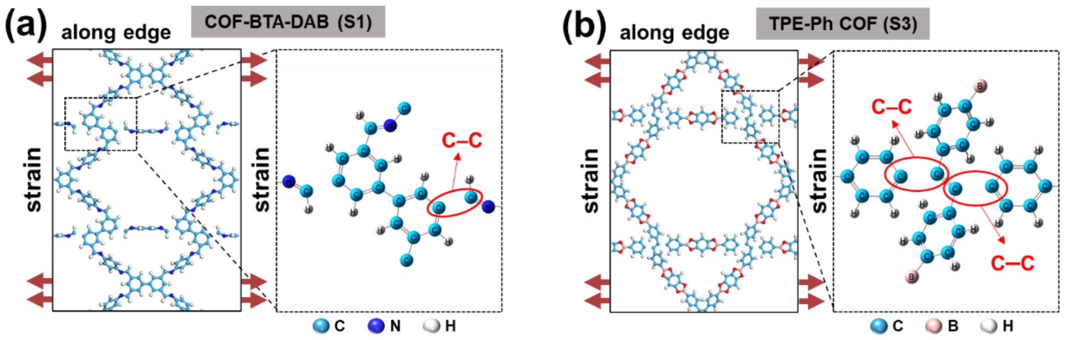

Figure S41. Illustrations of the locations of bond breakage for (a) COF-BTA-DAB (S1) and (b) TPE-Ph COF (S3) with star-pore topology stretched along the edge direction. Filled arrows show the stretching directions. Red circles show the locations of bond breakage.

18. Screening the mechanical properties of 2D COFs

The calculation protocol is as follows: We first select cores and linkers from a monomer database; the 2D COF structure is then constructed with pyCOFBuilder[80]; the topology, pore edge length, and linkage type are then used to predict the elastic moduli and Poisson's ratio by the relationships discovered in this study. Each 2D COF is encoded by a string like T3_BENZ_CHO_OH-L2_BENZ_NH2_H_H-HCB_A-AA. In this example, T3_BENZ_CHO_OH and L2_BENZ_NH2_H_H represent the core and linker units, respectively, each having the format of symmetry_core unit/linker unit functional group 1_functional group 2. HCB_A and AA represent the topology and stacking mode, respectively.

Table S9. Chemical structures of 100 randomly generated 2D COFs for illustrating the material screening protocol.

label	chemical structure	label	chemical structure
**1**	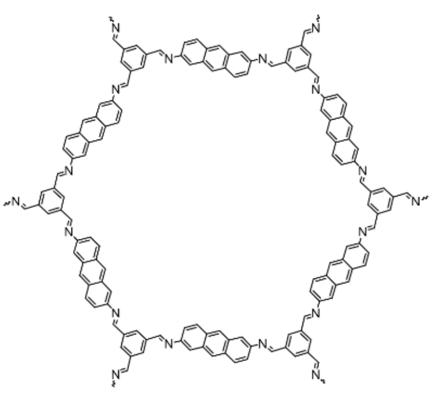	**2**	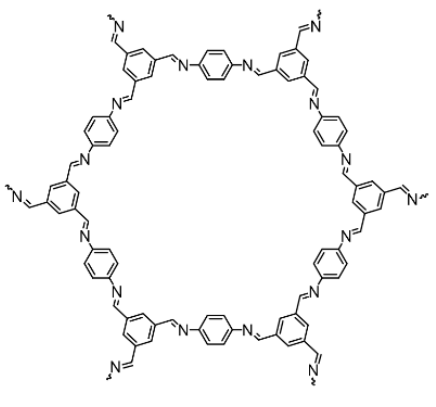
**3**	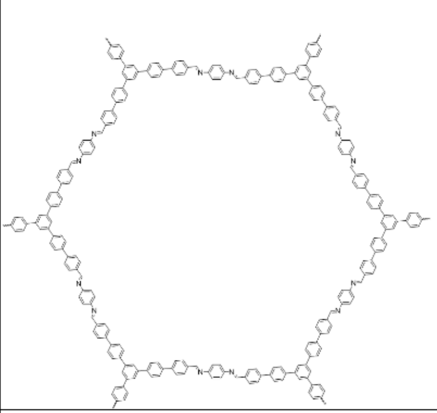	**4**	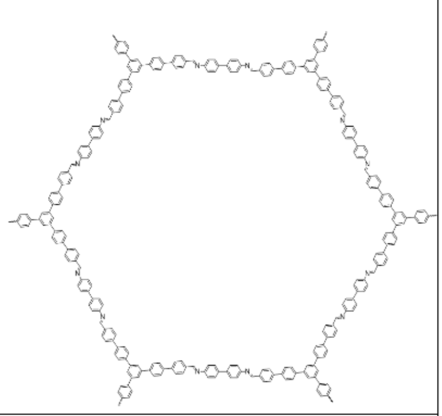
**5**	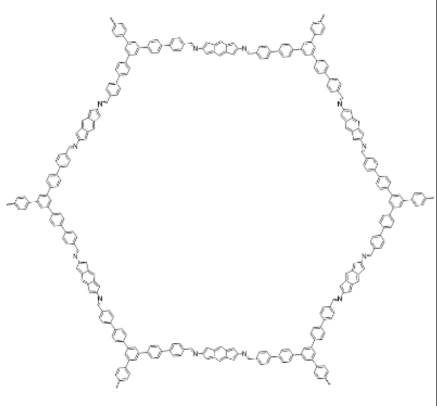	**6**	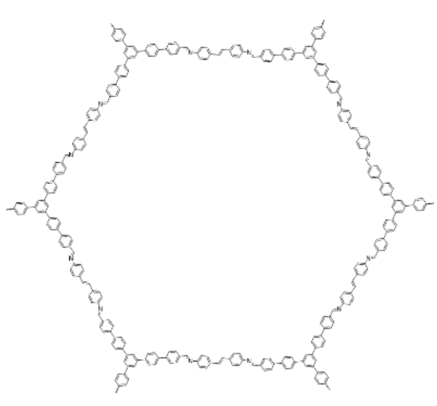

**7**	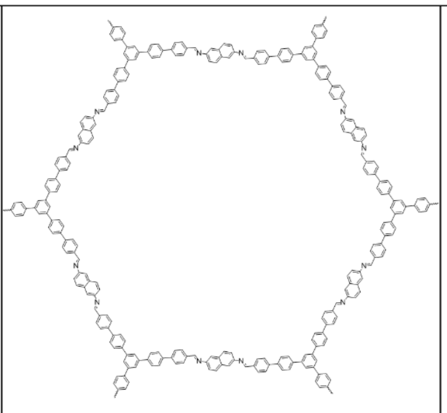	**8**	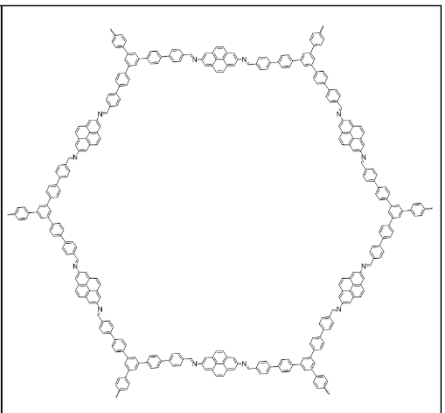
**9**	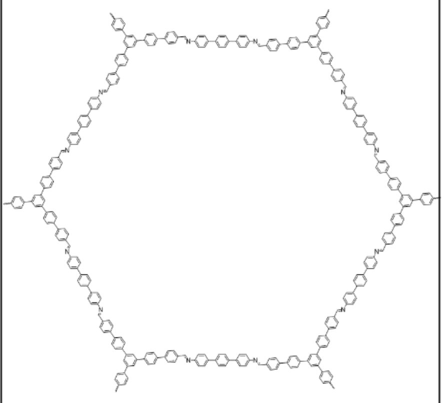	**10**	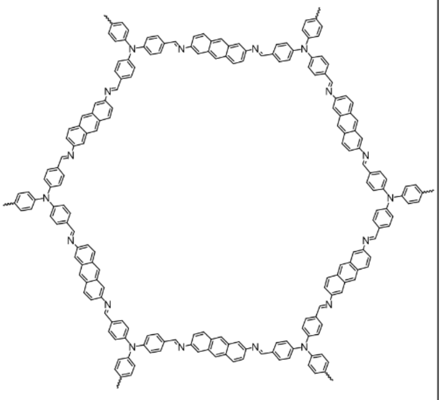
**11**	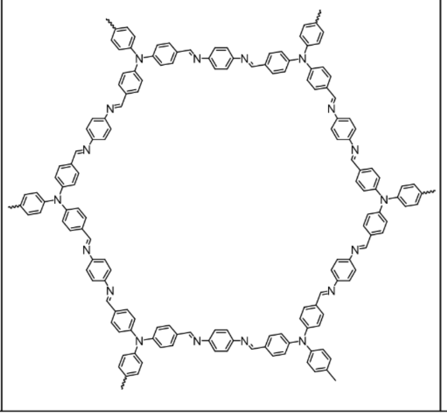	**12**	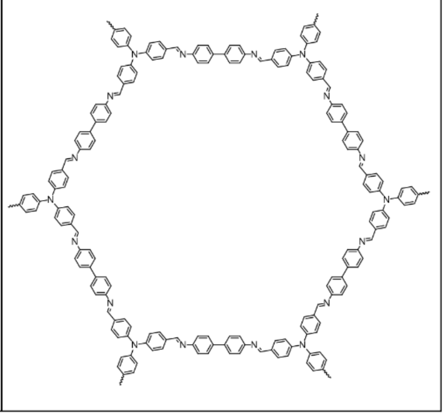

**13**	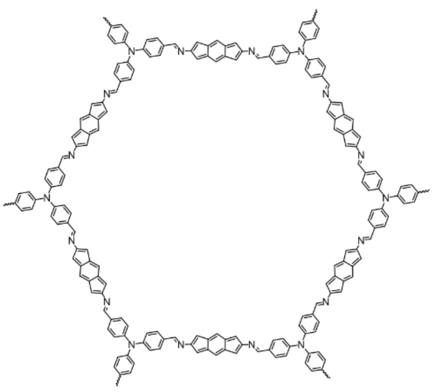	**14**	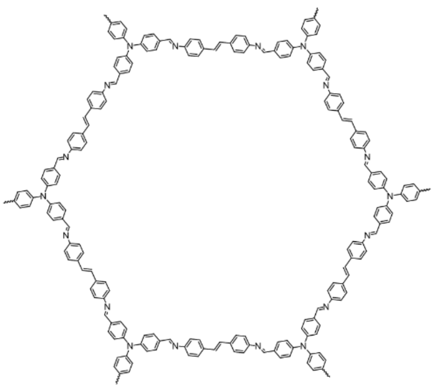
**15**	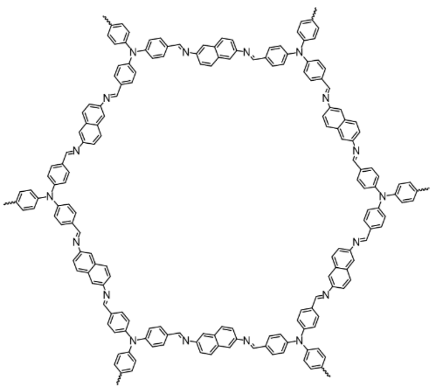	**16**	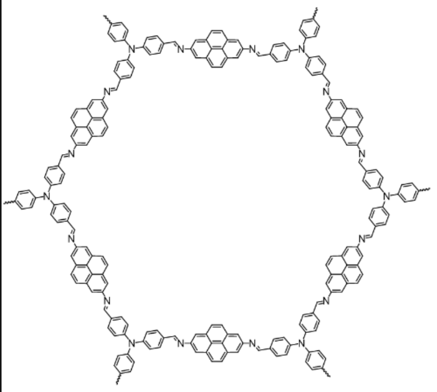
**17**	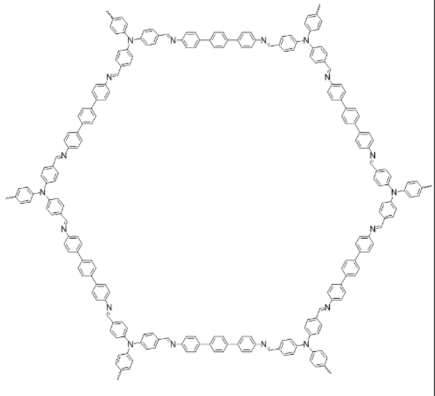	**18**	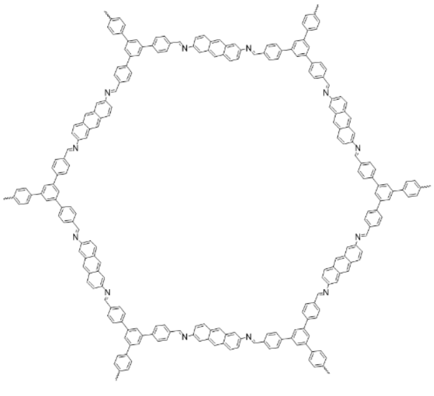

**19**	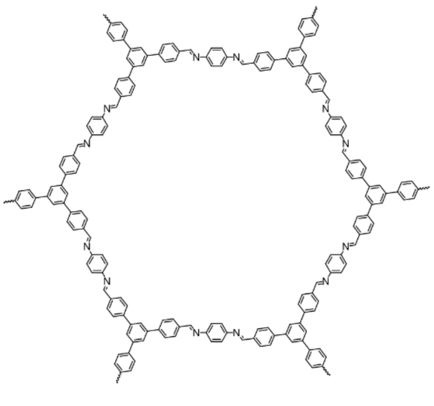	**20**	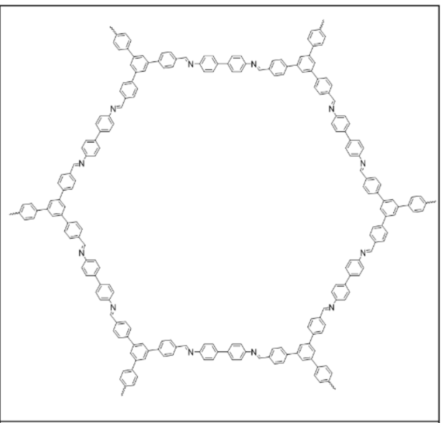
**21**	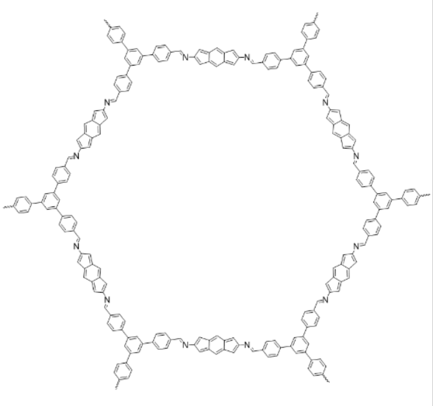	**22**	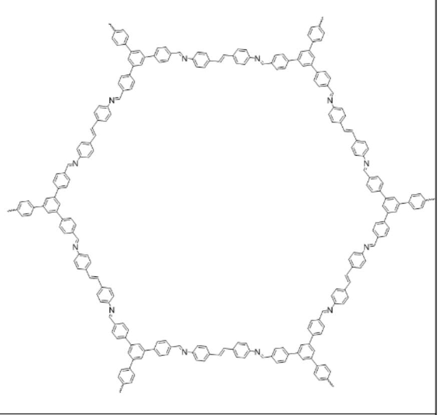
**23**	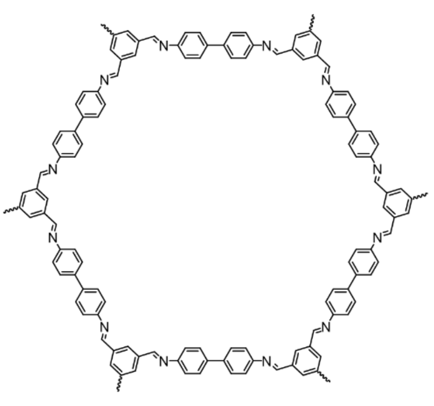	**24**	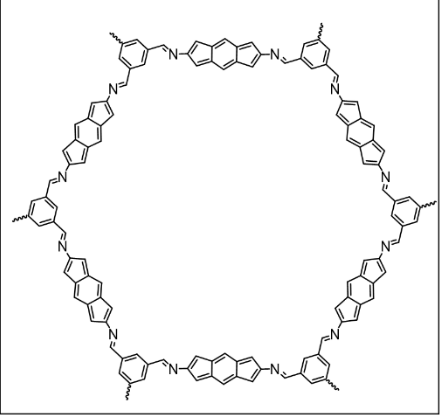

**25**	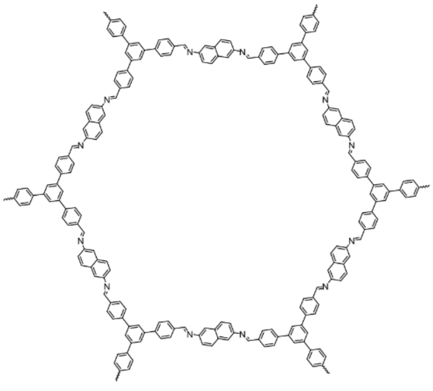	**26**	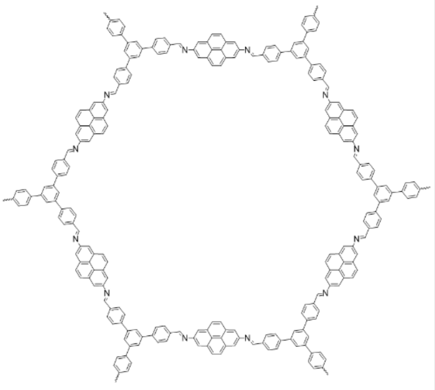
**27**	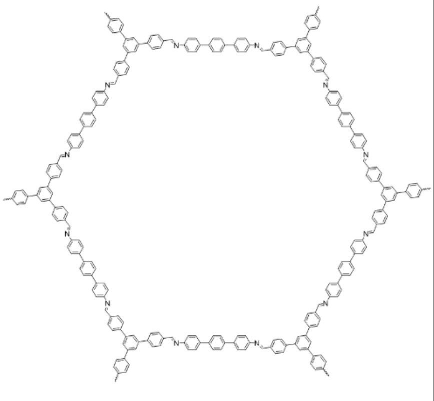	**28**	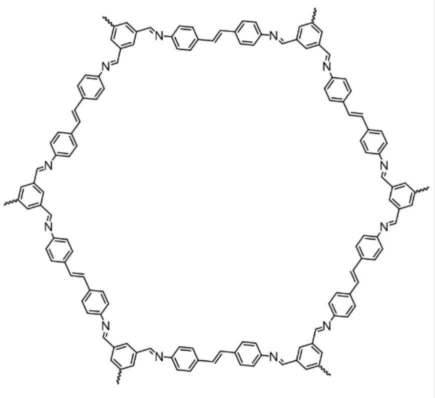
**29**	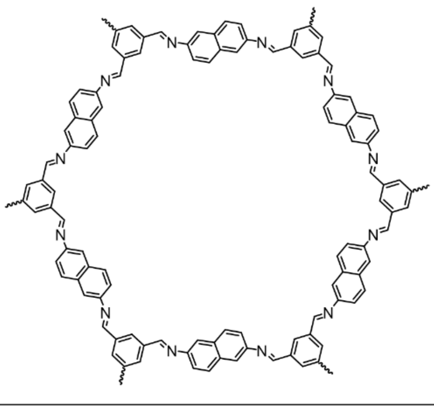	**30**	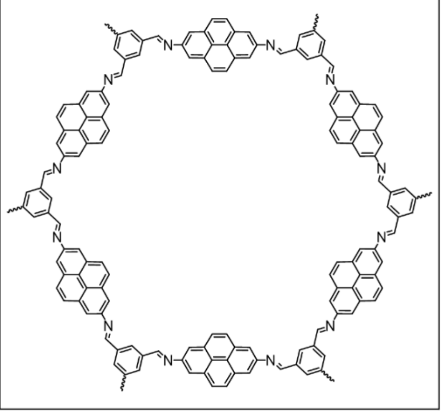

**31**	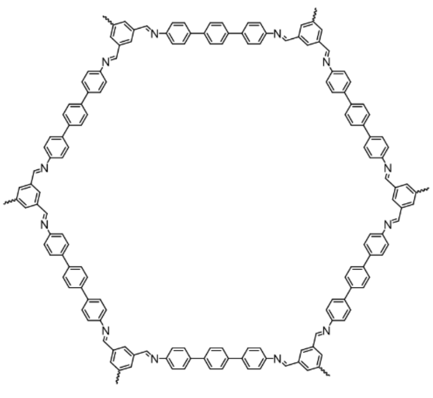	**32**	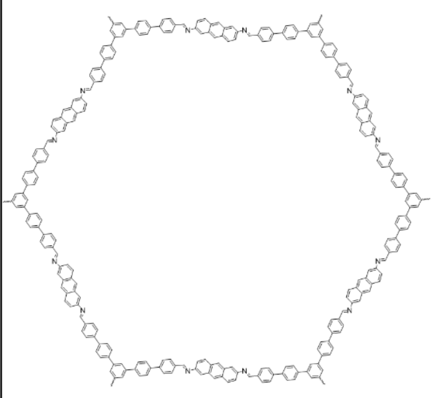
**33**	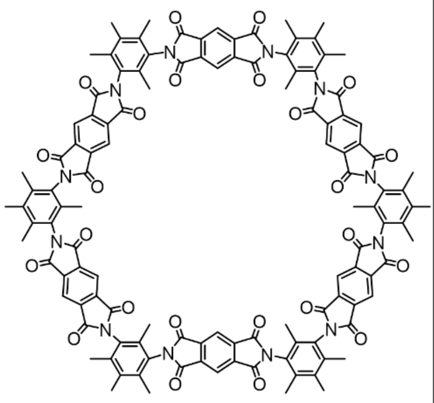	**34**	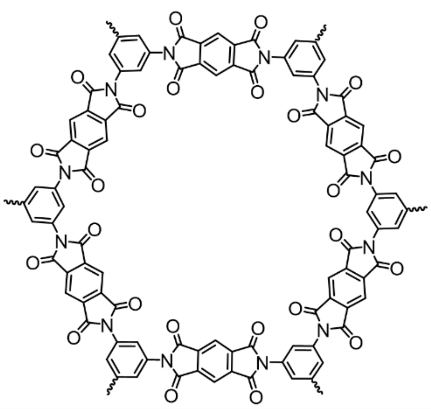
**35**	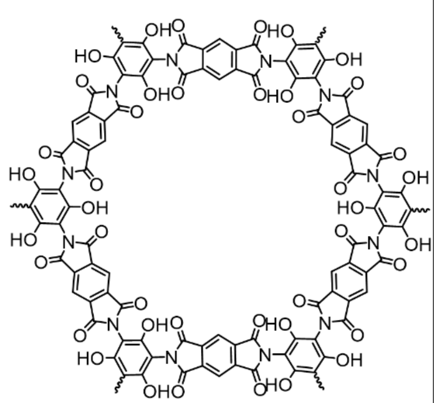	**36**	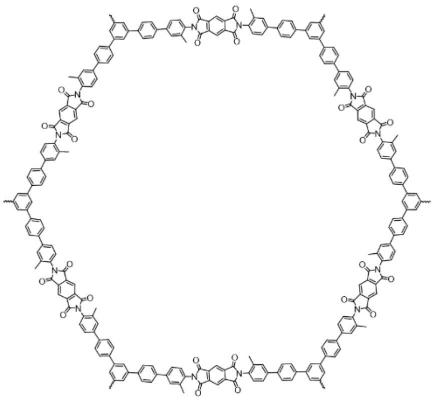

**37**	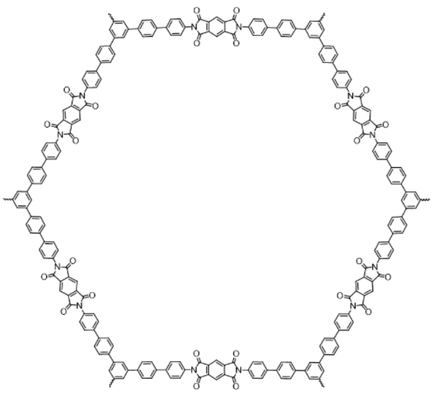	**38**	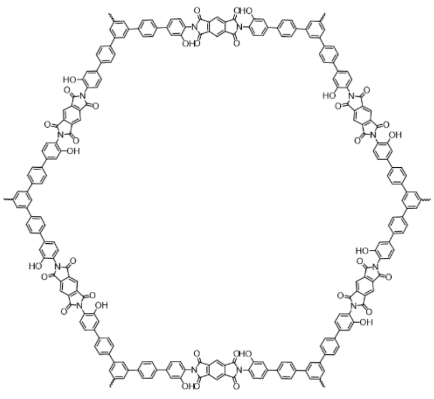
**39**	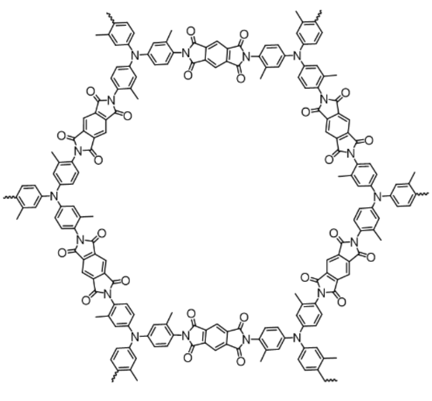	**40**	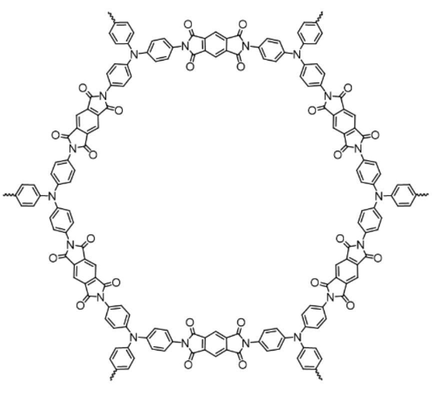
**41**	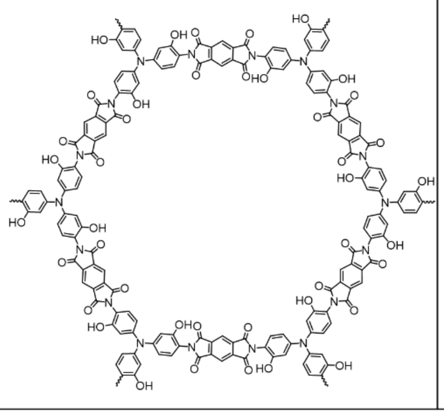	**42**	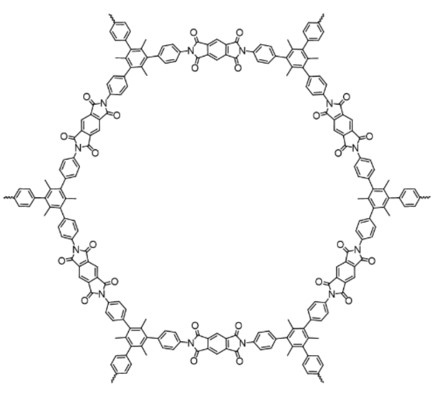

**43**	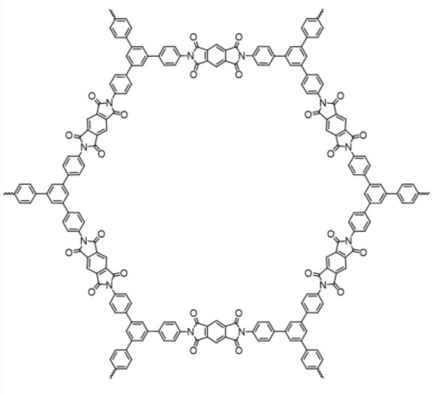	**44**	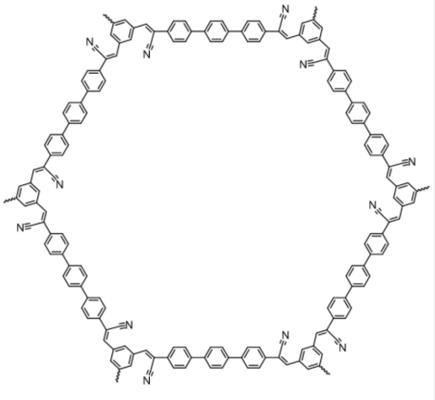
**45**	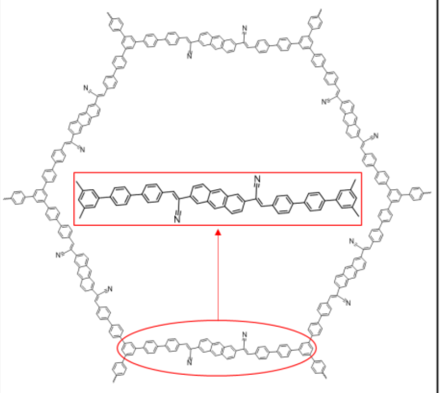	**46**	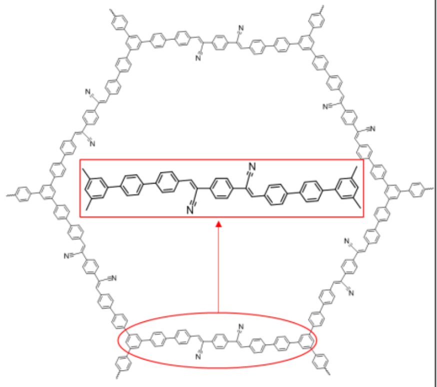
**47**	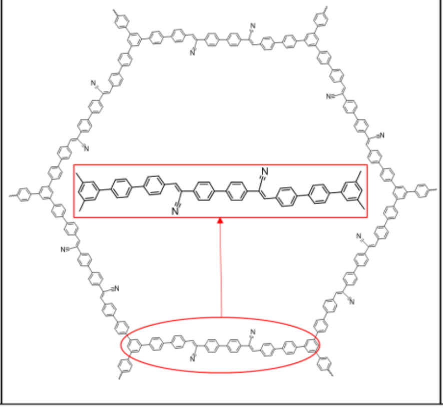	**48**	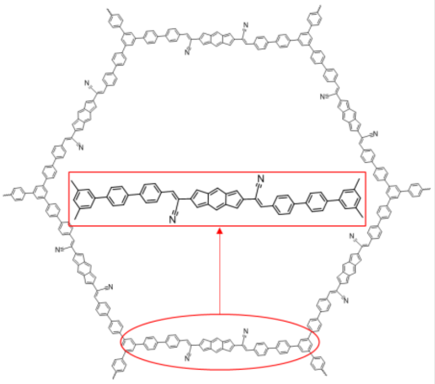

**49**	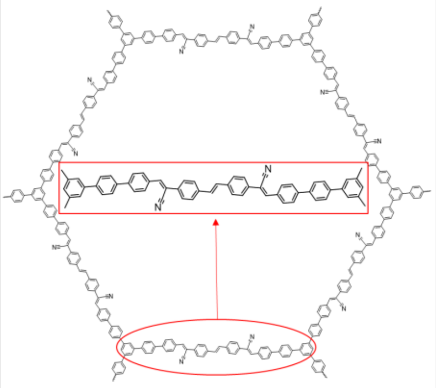	**50**	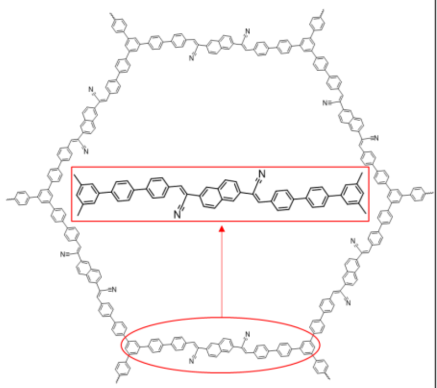
**51**	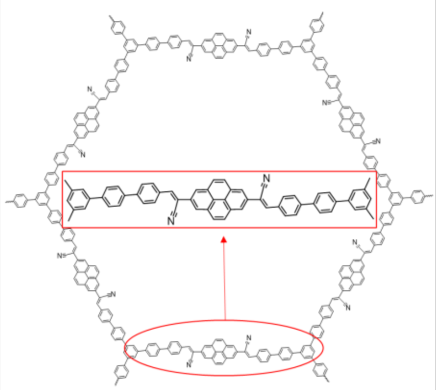	**52**	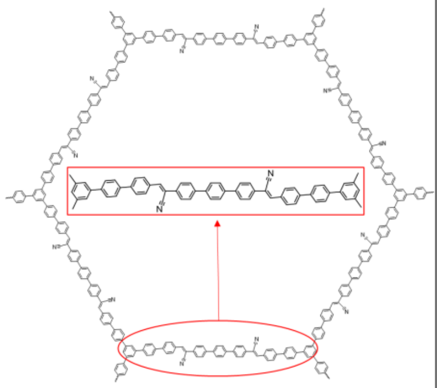
**53**	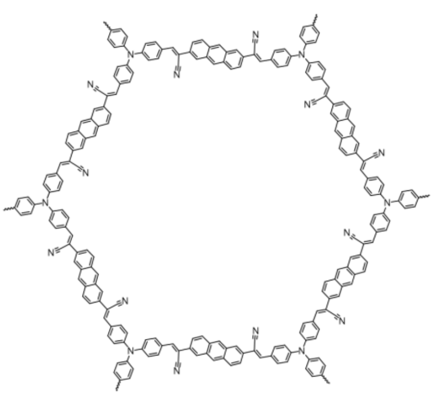	**54**	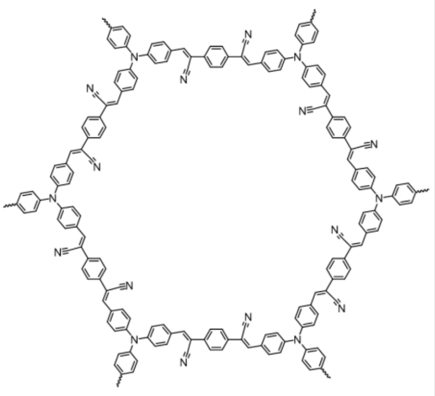

**55**	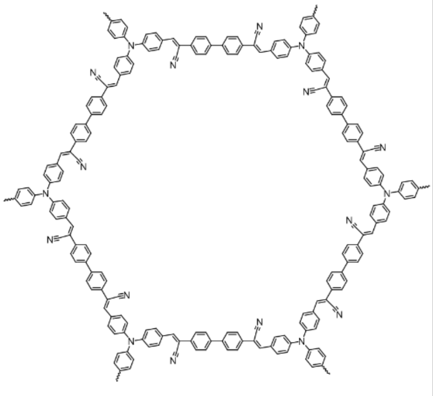	**56**	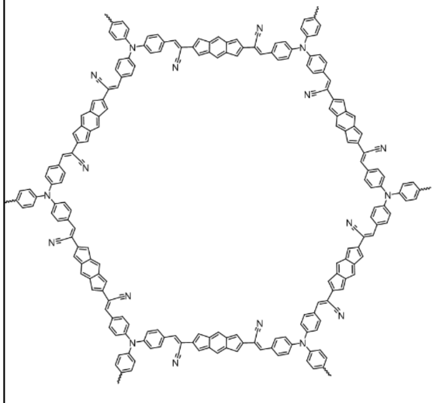
**57**	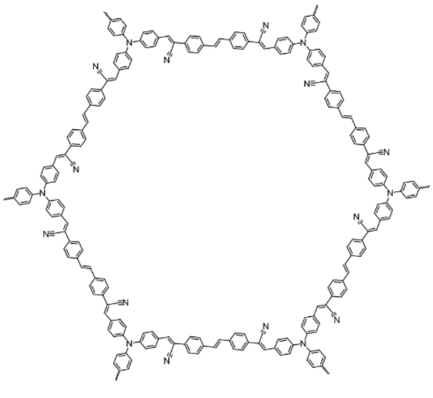	**58**	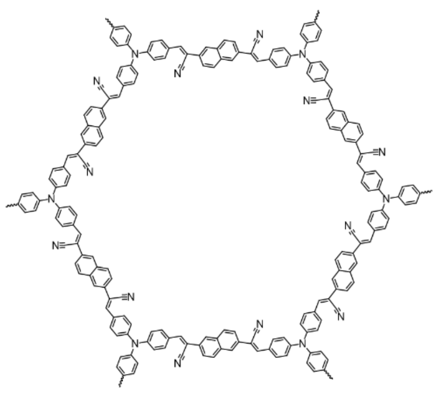
**59**	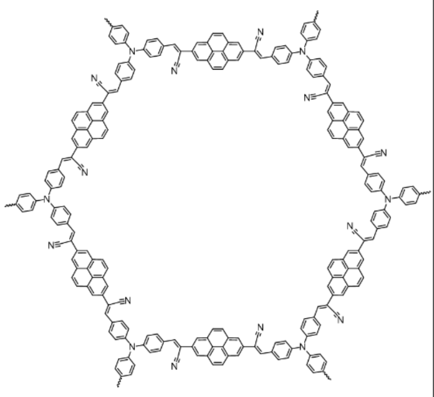	**60**	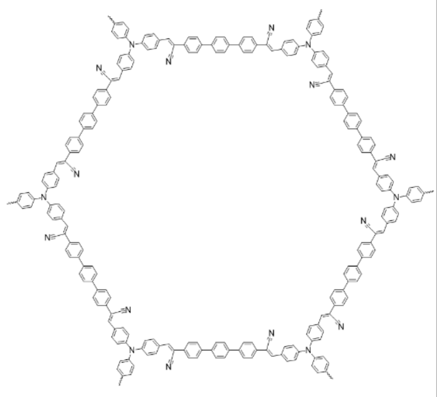

**61**	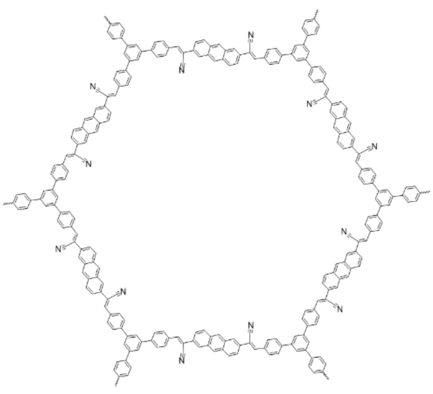	**62**	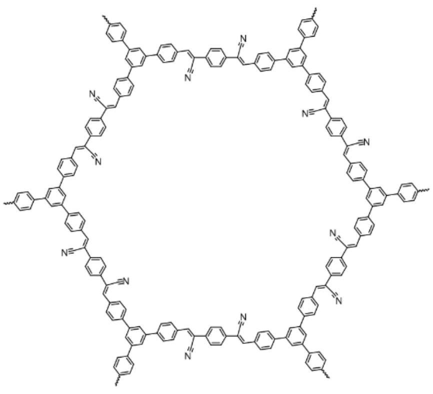
**63**	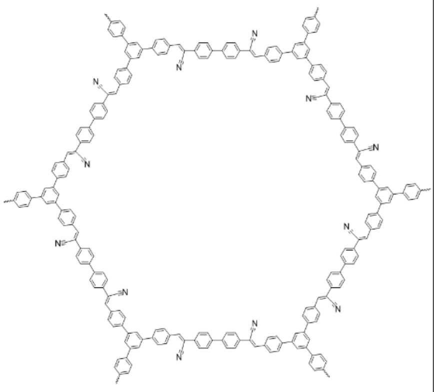	**64**	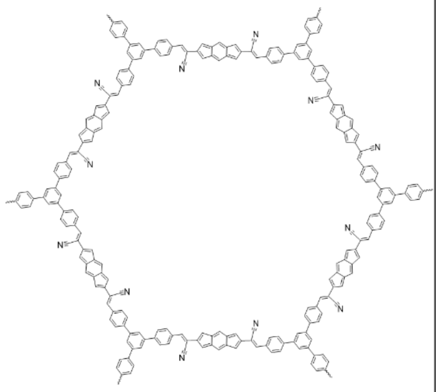
**65**	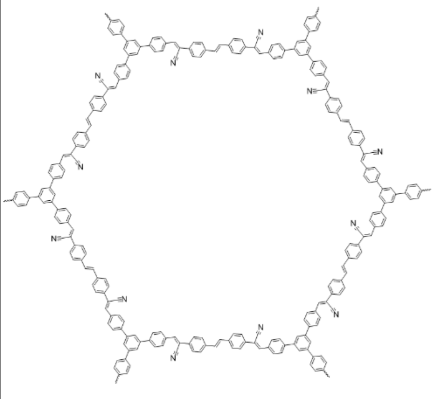	**66**	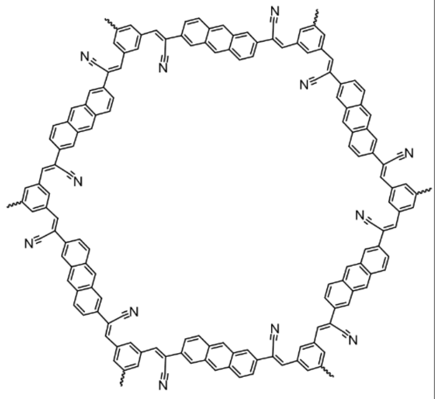

**67**	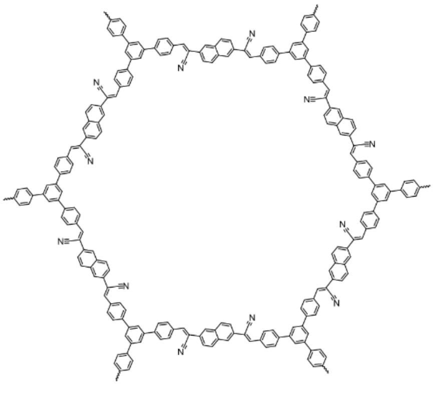	**68**	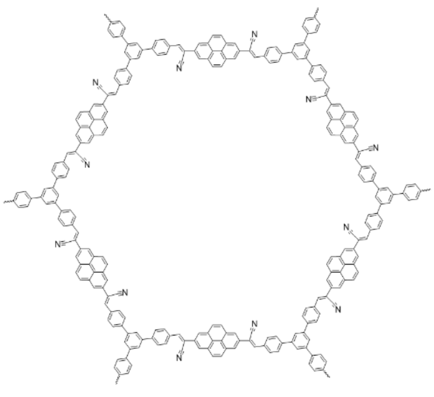
**69**	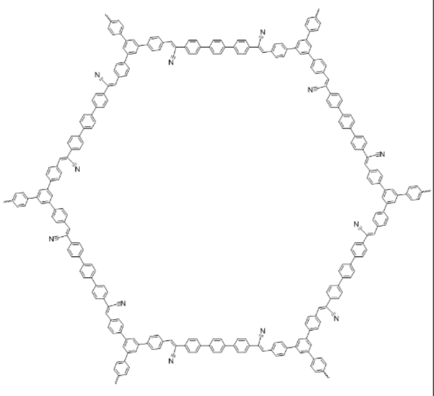	**70**	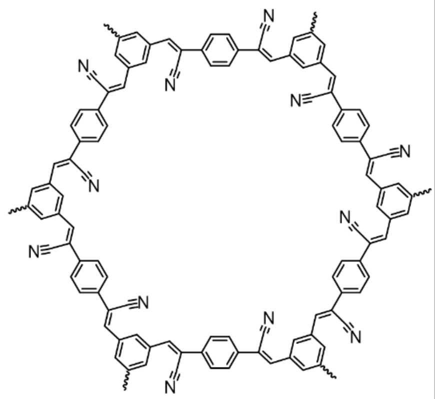
**71**	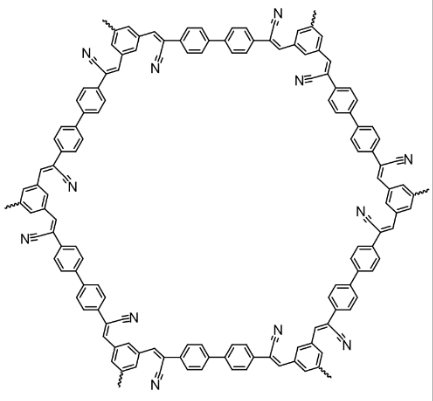	**72**	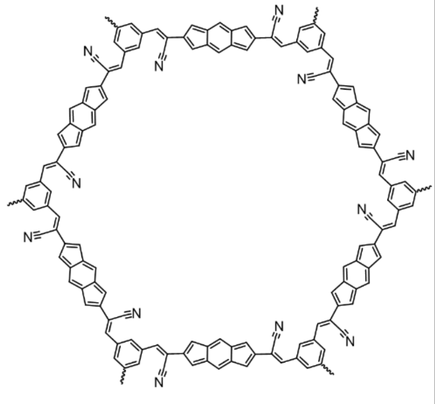

**73**	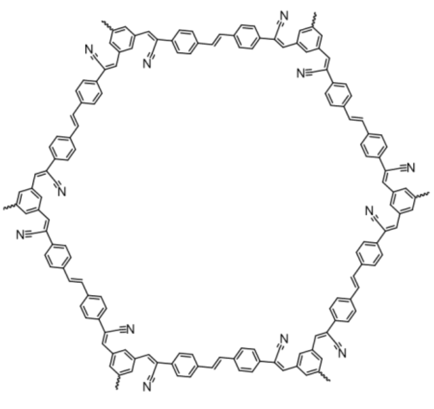	**74**	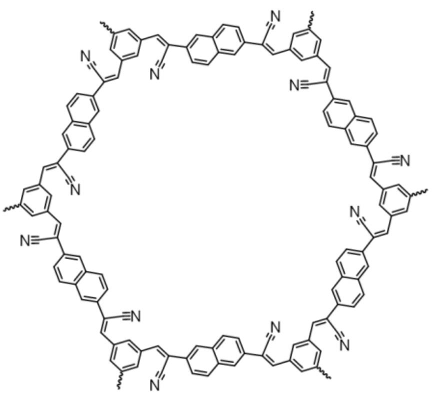
**75**	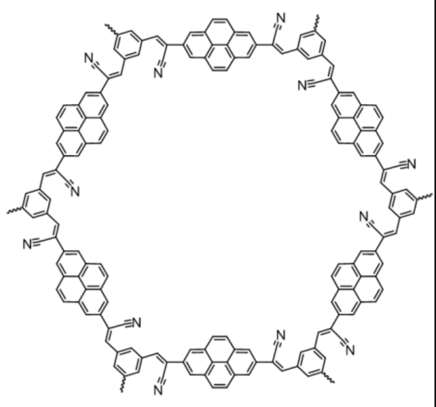	**76**	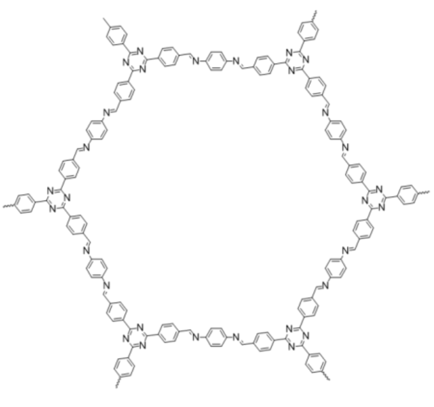
**77**	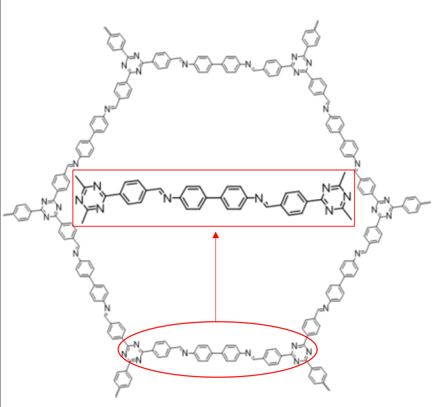	**78**	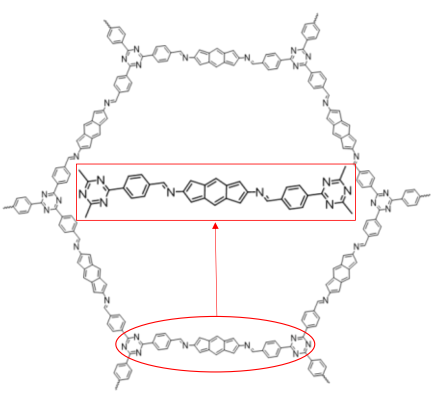

**79**	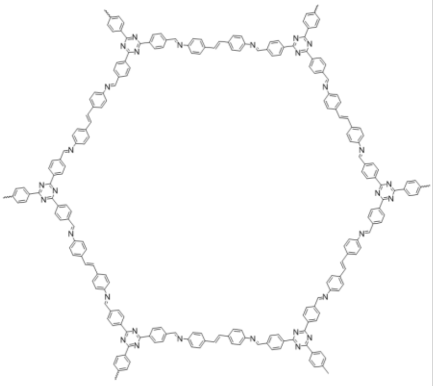	**80**	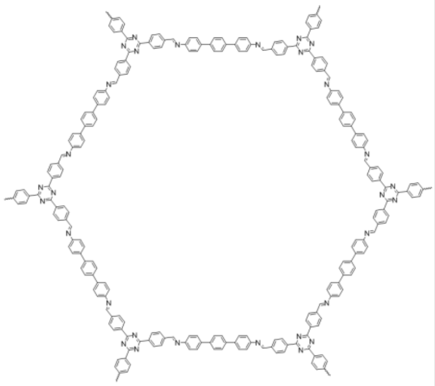
**81**	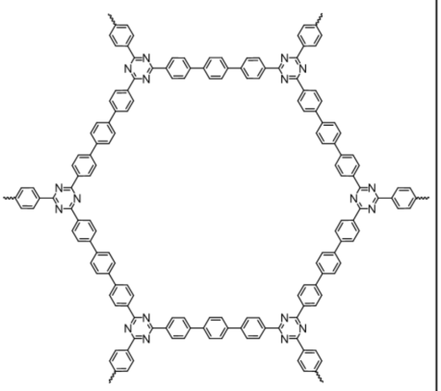	**82**	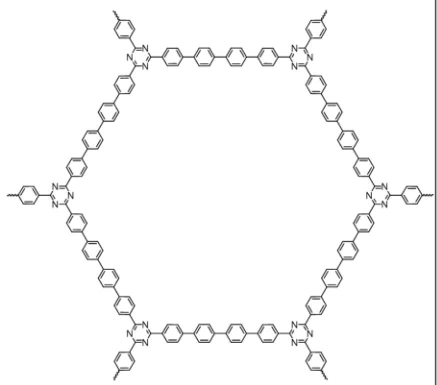
**83**	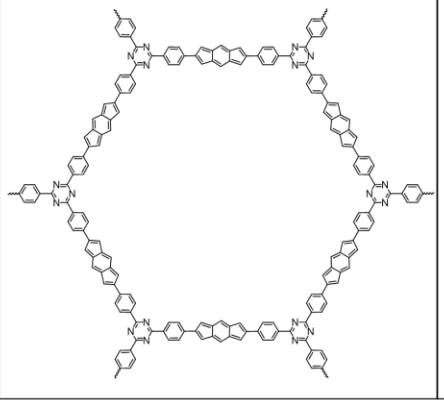	**84**	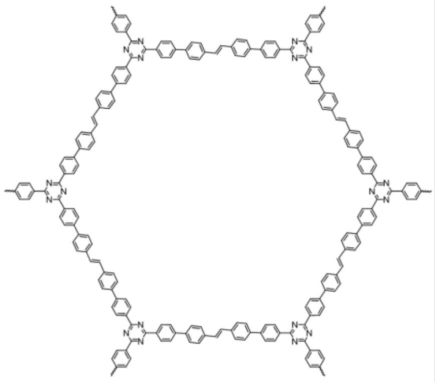

**85**	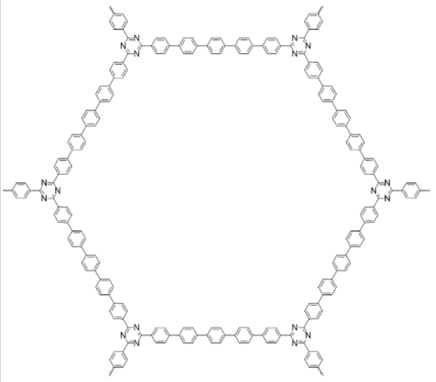	**86**	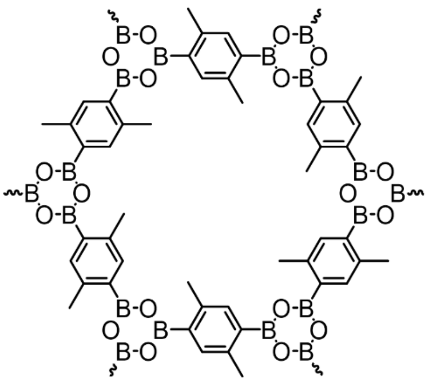
**87**	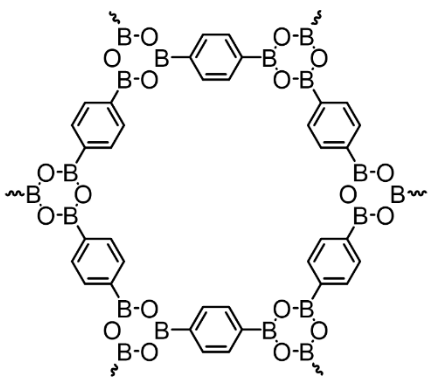	**88**	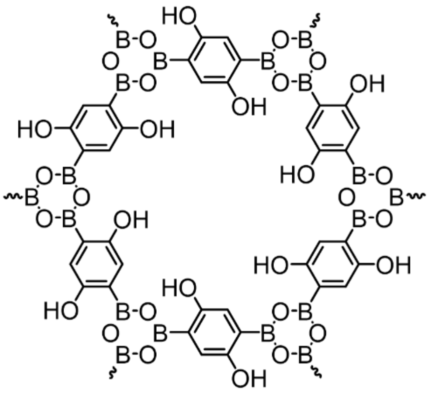
**89**	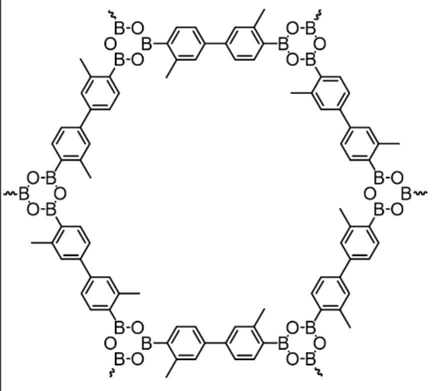	**90**	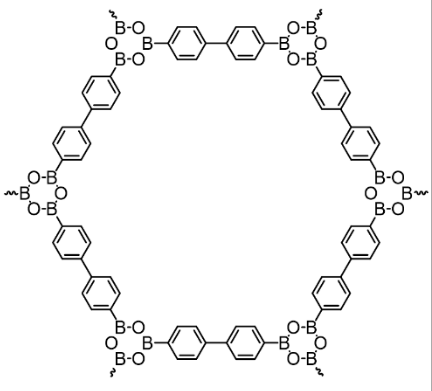

**91**	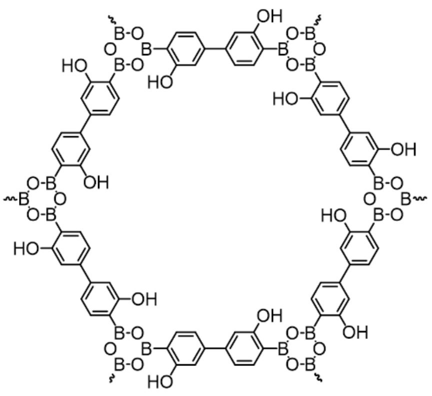	**92**	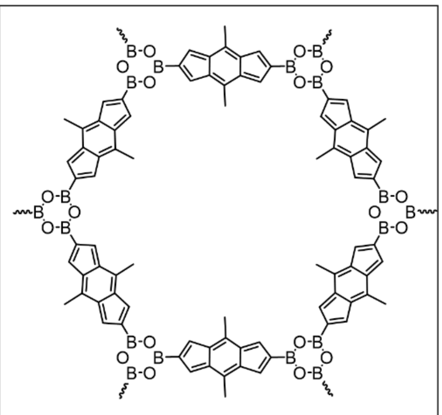
**93**	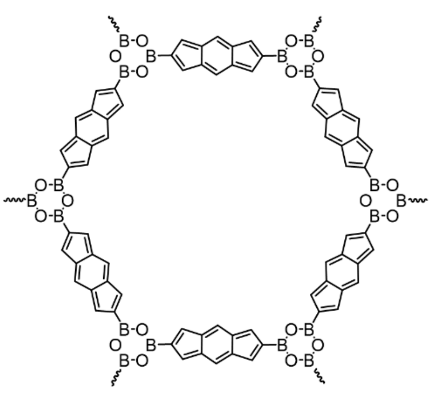	**94**	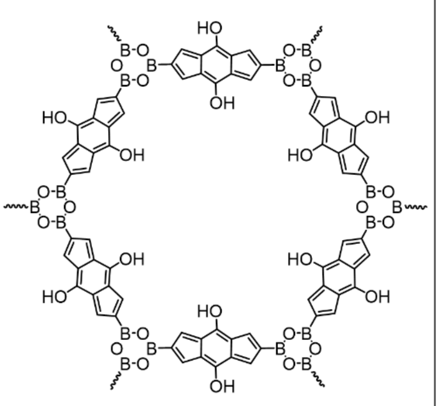
**95**	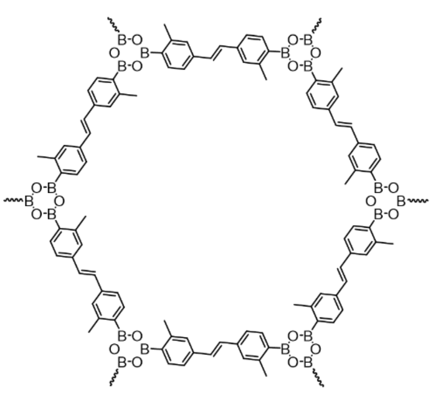	**96**	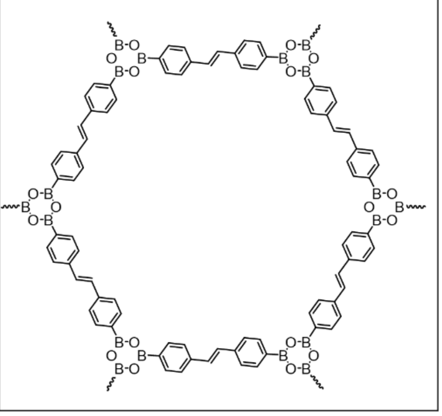

**97**	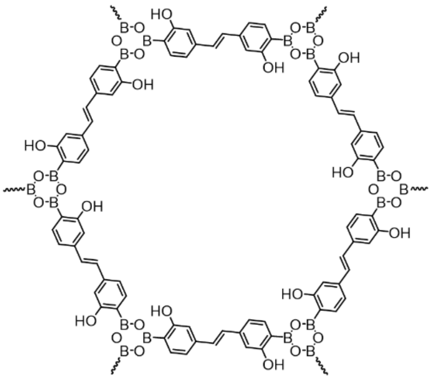	**98**	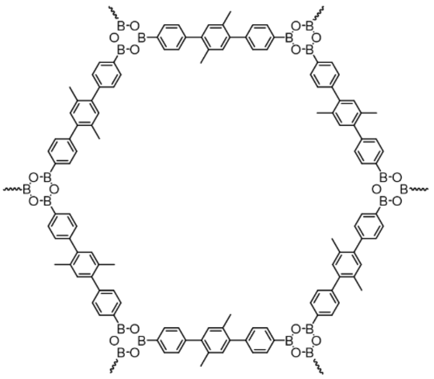
**99**	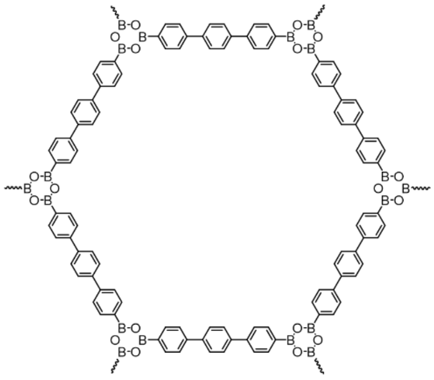	**100**	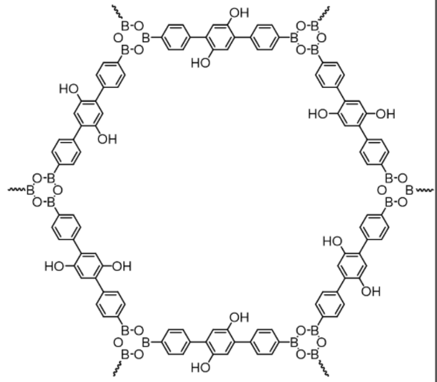

Table S10. Predicted elastic moduli of 100 randomly generated 2D COFs.

index	chemical linkage	pore edge length (Å)	predicted elastic moduli (N/m)
1	imine	17.42	1.90
2	imine	12.88	4.69
3	imine	29.96	0.37
4	imine	34.28	0.25
5	imine	34.01	0.25
6	imine	36.58	0.20
7	imine	32.17	0.30
8	imine	34.19	0.25
9	imine	38.61	0.17
10	imine	23.16	0.81
11	imine	18.62	1.55
12	imine	22.94	0.83
13	imine	22.67	0.86
14	imine	25.24	0.62
15	imine	20.83	1.11
16	imine	22.85	0.84
17	imine	27.27	0.49
18	imine	25.94	0.57
19	imine	21.39	1.02

20	imine	25.71	0.59
21	imine	25.45	0.61
22	imine	28.01	0.46
23	imine	17.19	1.97
24	imine	16.93	2.06
25	imine	23.60	0.76
26	imine	25.62	0.60
27	imine	30.04	0.37
28	imine	19.49	1.35
29	imine	15.09	2.92
30	imine	17.11	2.00
31	imine	21.52	1.00
32	imine	16.93	2.06
33	imide	13.10	2.75
34	imide	12.97	2.83
35	imide	12.97	2.83
36	imide	30.28	0.22
37	imide	30.28	0.22
38	imide	30.28	0.22
39	imide	18.85	0.92
40	imide	18.85	0.92
41	imide	18.85	0.92
42	imide	21.66	0.61
43	imide	21.66	0.61
44	cyanoethylene	22.02	1.42
45	cyanoethylene	35.02	0.35
46	cyanoethylene	30.49	0.53
47	cyanoethylene	34.80	0.36
48	cyanoethylene	34.54	0.37
49	cyanoethylene	37.10	0.30
50	cyanoethylene	32.69	0.43
51	cyanoethylene	34.72	0.36
52	cyanoethylene	39.12	0.25
53	cyanoethylene	23.68	1.14
54	cyanoethylene	19.15	2.16
55	cyanoethylene	23.46	1.17
56	cyanoethylene	23.20	1.21
57	cyanoethylene	25.76	0.89
58	cyanoethylene	21.35	1.56
59	cyanoethylene	23.38	1.19
60	cyanoethylene	27.79	0.71
61	cyanoethylene	26.45	0.82
62	cyanoethylene	21.92	1.44
63	cyanoethylene	26.24	0.84

64	cyanoethylene	25.97	0.86
65	cyanoethylene	28.54	0.65
66	cyanoethylene	17.94	2.63
67	cyanoethylene	24.13	1.08
68	cyanoethylene	26.15	0.85
69	cyanoethylene	30.57	0.53
70	cyanoethylene	13.41	6.29
71	cyanoethylene	17.69	2.74
72	cyanoethylene	17.46	2.85
73	cyanoethylene	20.05	1.88
74	cyanoethylene	15.64	3.96
75	cyanoethylene	17.66	2.75
76	triazine	21.39	0.90
77	triazine	25.71	0.52
78	triazine	25.42	0.54
79	triazine	27.87	0.41
80	triazine	30.04	0.33
81	triazine	17.30	1.71
82	triazine	21.61	0.88
83	triazine	21.35	0.91
84	triazine	23.92	0.65
85	triazine	25.94	0.51
86	boroxine	8.48	11.56
87	boroxine	8.48	11.56
88	boroxine	8.48	11.56
89	boroxine	12.80	3.36
90	boroxine	12.80	3.36
91	boroxine	12.80	3.36
92	boroxine	12.53	3.58
93	boroxine	12.53	3.58
94	boroxine	12.53	3.58
95	boroxine	15.10	2.05
96	boroxine	15.10	2.05
97	boroxine	15.10	2.05
98	boroxine	17.13	1.40
99	boroxine	17.13	1.40
100	boroxine	17.13	1.40

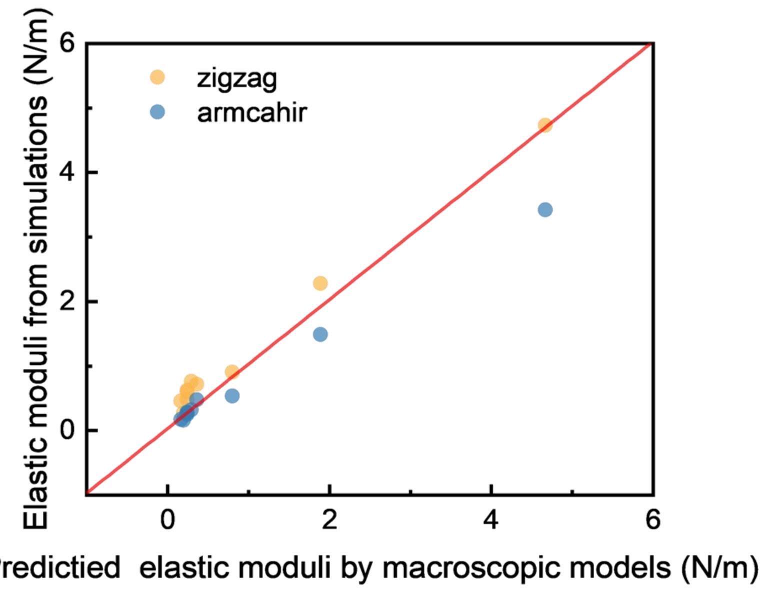

Figure S42. Calculated elastic moduli of the new COF structures in **Figure 5** using the DFTB Method.

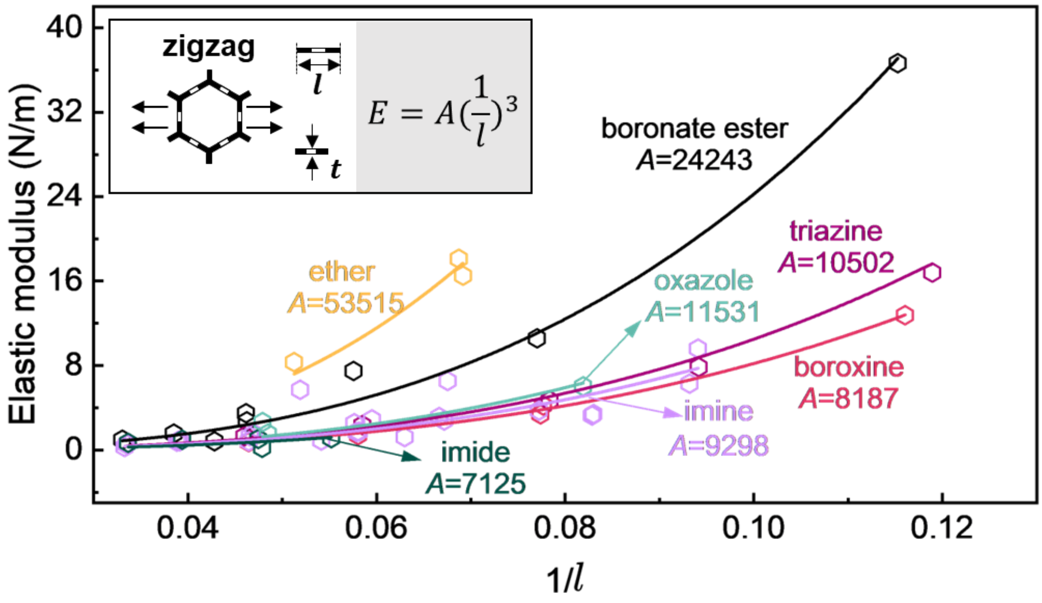

Figure S43. Relationships between the elastic modulus and the inverse of pore edge length (l) for hexagonal 2D COFs when stretched in the zigzag direction.

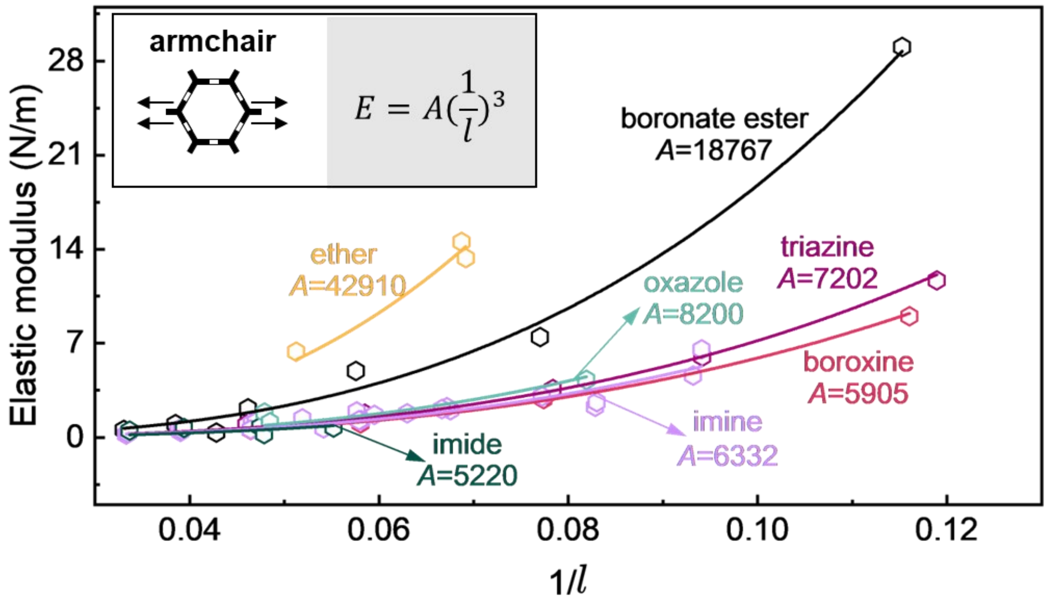

Figure S44. Relationships between the elastic modulus and the inverse of pore edge length for hexagonal 2D COFs when stretched in the armchair direction.

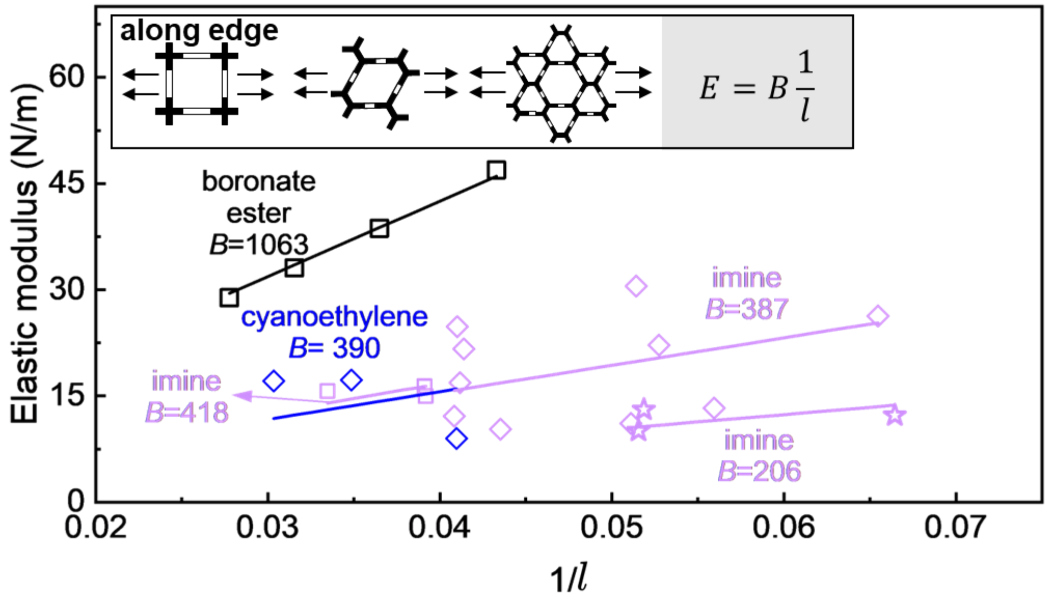

Figure S45. Relationships between the elastic modulus and the inverse of pore edge length for tetragonal, rhombic, and star-pore 2D COFs when stretched along the pore edge direction.

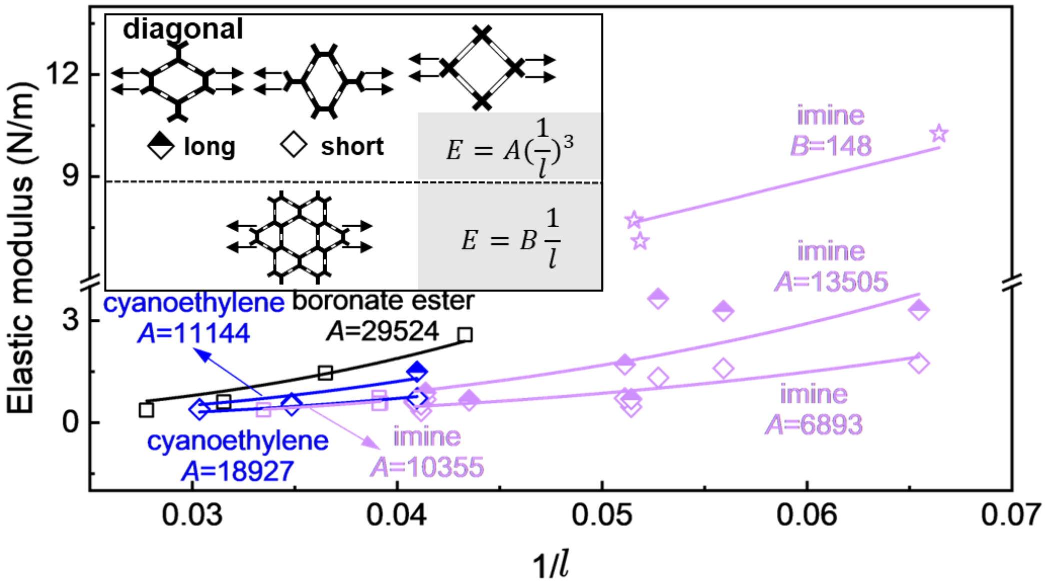

Figure S46. Relationships between the elastic modulus and the inverse of pore edge length for tetragonal, rhombic, and star-pore 2D COFs when stretched along diagonal direction.

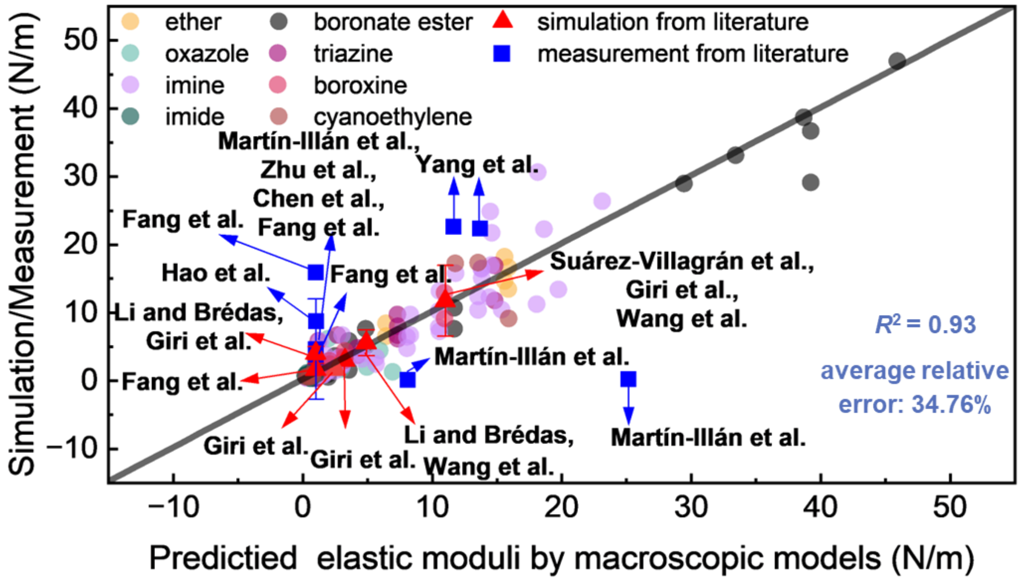

Figure S47. Comparative analysis of the predicted elastic moduli of 2D COFs obtained using macroscopic models.

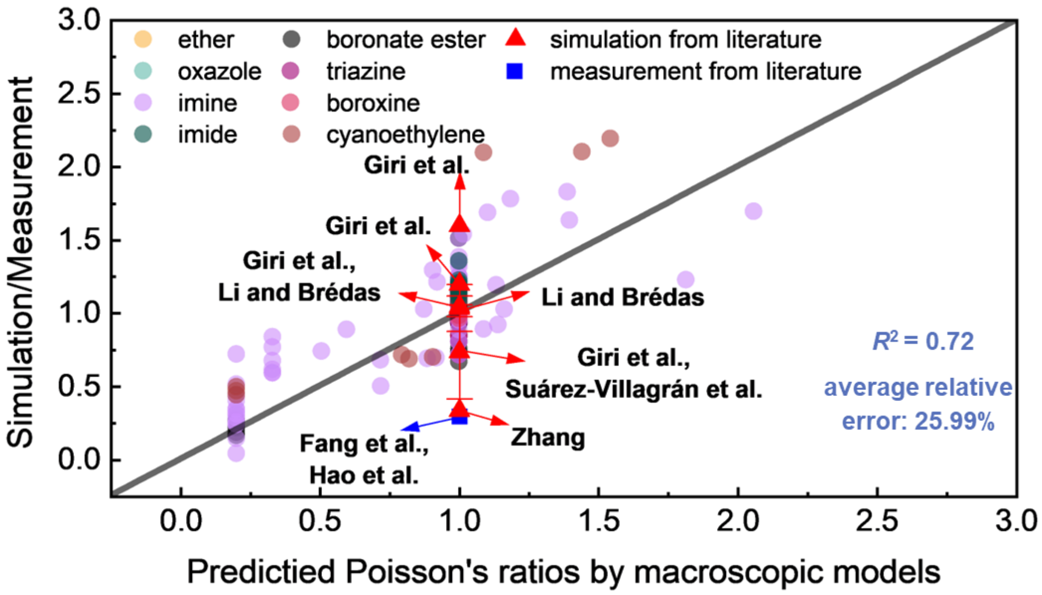

Figure S48. Comparative analysis of the predicted Poisson's ratios of 2D COFs obtained using macroscopic models.

Supplementary References

(1) Kudin, K. N.; Scuseria, G. E.; Yakobson, B. I. C_2F, BN, and C Nanoshell Elasticity from Ab Initio Computations. *Phys. Rev. B* **2001**, *64* (23), 235406.

(2) Tan, X.; Wu, J.; Zhang, K.; Peng, X.; Sun, L.; Zhong, J. Nanoindentation Models and Young's Modulus of Monolayer Graphene: A Molecular Dynamics Study. *Appl. Phys. Lett.* **2013**, *102* (7).

(3) Koenig, S. P.; Boddeti, N. G.; Dunn, M. L.; Bunch, J. S. Ultrastrong Adhesion of Graphene Membranes. *Nat. Nanotechnol.* **2011**, *6* (9), 543–546.

(4) Politano, A.; Chiarello, G. Probing the Young's Modulus and Poisson's Ratio in Graphene/Metal Interfaces and Graphite: A Comparative Study. *Nano Res.* **2015**, *8* (6), 1847–1856.

(5) Kuhn, P.; Antonietti, M.; Thomas, A. Porous, Covalent Triazine-Based Frameworks Prepared by Ionothermal Synthesis. *Angew. Chem. Int. Ed.* **2008**, *47* (18), 3450–3453.

(6) Côté, A. P.; El-Kaderi, H. M.; Furukawa, H.; Hunt, J. R.; Yaghi, O. M. Reticular Synthesis of Microporous and Mesoporous 2D Covalent Organic Frameworks. *J. Am. Chem. Soc.* **2007**, *129* (43), 12914–12915.

(7) Jackson, K. T.; Reich, T. E.; El-Kaderi, H. M. Targeted Synthesis of a Porous Borazine-Linked Covalent Organic Framework. *Chem. Commun.* **2012**, *48* (70), 8823.

(8) Côté, A. P.; Benin, A. I.; Ockwig, N. W.; O'Keeffe, M.; Matzger, A. J.; Yaghi, O. M. Porous, Crystalline, Covalent Organic Frameworks. *Science* **2005**, *310* (5751), 1166–1170.

(9) Li, Z.; Feng, X.; Zou, Y.; Zhang, Y.; Xia, H.; Liu, X.; Mu, Y. A 2D Azine-Linked Covalent Organic Framework for Gas Storage Applications. *Chem. Commun.* **2014**, *50* (89), 13825–13828.

(10) Gao, Q.; Bai, L.; Zhang, X.; Wang, P.; Li, P.; Zeng, Y.; Zou, R.; Zhao, Y. Synthesis of Microporous Nitrogen-Rich Covalent-Organic Framework and Its Application in CO_2 Capture. *Chin. J. Chem.* **2015**, *33* (1), 90–94.

(11) Bojdys, M. J.; Jeromenok, J.; Thomas, A.; Antonietti, M. Rational Extension of the Family of Layered, Covalent, Triazine-Based Frameworks with Regular Porosity. *Adv. Mater.* **2010**, *22* (19), 2202–2205.

(12) Wan, S.; Guo, J.; Kim, J.; Ihee, H.; Jiang, D. A Photoconductive Covalent Organic Framework: Self-Condensed Arene Cubes Composed of Eclipsed 2D Polypyrene Sheets for Photocurrent Generation. *Angew. Chem. Int. Ed.* **2009**, *48* (30), 5439–5442.

(13) Wei, P.-F.; Qi, M.-Z.; Wang, Z.-P.; Ding, S.-Y.; Yu, W.; Liu, Q.; Wang, L.-K.; Wang, H.-Z.; An, W.-K.; Wang, W. Benzoxazole-Linked Ultrastable Covalent Organic Frameworks for Photocatalysis. *J. Am. Chem. Soc.* **2018**, *140* (13), 4623–4631.

(14) Peng, Y.; Huang, Y.; Zhu, Y.; Chen, B.; Wang, L.; Lai, Z.; Zhang, Z.; Zhao, M.; Tan, C.; Yang, N.; Shao, F.; Han, Y.; Zhang, H. Ultrathin Two-Dimensional

Covalent Organic Framework Nanosheets: Preparation and Application in Highly Sensitive and Selective DNA Detection. *J. Am. Chem. Soc.* **2017**, *139* (25), 8698–8704.
(15) Ding, S.-Y.; Gao, J.; Wang, Q.; Zhang, Y.; Song, W.-G.; Su, C.-Y.; Wang, W. Construction of Covalent Organic Framework for Catalysis: Pd/COF-LZU1 in Suzuki-Miyaura Coupling Reaction. *J. Am. Chem. Soc.* **2011**, *133* (49), 19816–19822.
(16) Guan, X.; Li, H.; Ma, Y.; Xue, M.; Fang, Q.; Yan, Y.; Valtchev, V.; Qiu, S. Chemically Stable Polyarylether-Based Covalent Organic Frameworks. *Nat. Chem.* **2019**, *11* (6), 587–594.
(17) Zhang, B.; Wei, M.; Mao, H.; Pei, X.; Alshmimri, S. A.; Reimer, J. A.; Yaghi, O. M. Crystalline Dioxin-Linked Covalent Organic Frameworks from Irreversible Reactions. *J. Am. Chem. Soc.* **2018**, *140* (40), 12715–12719.
(18) Wang, P.; Xu, Q.; Li, Z.; Jiang, W.; Jiang, Q.; Jiang, D. Exceptional Iodine Capture in 2D Covalent Organic Frameworks. *Adv. Mater.* **2018**, *30* (29), 1801991.
(19) Chen, X.; Addicoat, M.; Jin, E.; Xu, H.; Hayashi, T.; Xu, F.; Huang, N.; Irle, S.; Jiang, D. Designed Synthesis of Double-Stage Two-Dimensional Covalent Organic Frameworks. *Sci. Rep.* **2015**, *5* (1), 14650.
(20) Zhai, L.; Huang, N.; Xu, H.; Chen, Q.; Jiang, D. A Backbone Design Principle for Covalent Organic Frameworks: The Impact of Weakly Interacting Units on CO_2 Adsorption. *Chem. Commun.* **2017**, *53* (30), 4242–4245.
(21) Albacete, P.; Martínez, J. I.; Li, X.; López-Moreno, A.; Mena-Hernando, S.; Platero-Prats, A. E.; Montoro, C.; Loh, K. P.; Pérez, E. M.; Zamora, F. Layer-Stacking-Driven Fluorescence in a Two-Dimensional Imine-Linked Covalent Organic Framework. *J. Am. Chem. Soc.* **2018**, *140* (40), 12922–12929.
(22) Wang, L.; Dong, B.; Ge, R.; Jiang, F.; Xu, J. Fluorene-Based Two-Dimensional Covalent Organic Framework with Thermoelectric Properties through Doping. *ACS Appl. Mater. Interfaces* **2017**, *9* (8), 7108–7114.
(23) Shinde, D. B.; Aiyappa, H. B.; Bhadra, M.; Biswal, B. P.; Wadge, P.; Kandambeth, S.; Garai, B.; Kundu, T.; Kurungot, S.; Banerjee, R. A Mechanochemically Synthesized Covalent Organic Framework as a Proton-Conducting Solid Electrolyte. *J. Mater. Chem. A* **2016**, *4* (7), 2682–2690.
(24) Gao, Q.; Bai, L.; Zeng, Y.; Wang, P.; Zhang, X.; Zou, R.; Zhao, Y. Reconstruction of Covalent Organic Frameworks by Dynamic Equilibrium. *Chem. Eur. J.* **2015**, *21* (47), 16818–16822.
(25) Lyu, H.; Diercks, C. S.; Zhu, C.; Yaghi, O. M. Porous Crystalline Olefin-Linked Covalent Organic Frameworks. *J. Am. Chem. Soc.* **2019**, *141* (17), 6848–6852.
(26) Fang, Q.; Zhuang, Z.; Gu, S.; Kaspar, R. B.; Zheng, J.; Wang, J.; Qiu, S.; Yan, Y. Designed Synthesis of Large-Pore Crystalline Polyimide Covalent Organic Frameworks. *Nat. Commun.* **2014**, *5* (1), 4503.
(27) Mullangi, D.; Dhavale, V.; Shalini, S.; Nandi, S.; Collins, S.; Woo, T.; Kurungot, S.; Vaidhyanathan, R. Low-Overpotential Electrocatalytic Water Splitting with Noble-Metal-Free Nanoparticles Supported in a Sp^3 N-Rich Flexible COF. *Adv. Energy Mater.* **2016**, *6* (13), 1600110.

(28) Wan, S.; Guo, J.; Kim, J.; Ihee, H.; Jiang, D. A Belt-Shaped, Blue Luminescent, and Semiconducting Covalent Organic Framework. *Angew. Chem. Int. Ed.* **2008**, *47* (46), 8826–8830.
(29) Zhu, X.; An, S.; Liu, Y.; Hu, J.; Liu, H.; Tian, C.; Dai, S.; Yang, X.; Wang, H.; Abney, C. W.; Dai, S. Efficient Removal of Organic Dye Pollutants Using Covalent Organic Frameworks. *AICHE J.* **2017**, *63* (8), 3470–3478.
(30) Kandambeth, S.; Venkatesh, V.; Shinde, D. B.; Kumari, S.; Halder, A.; Verma, S.; Banerjee, R. Self-Templated Chemically Stable Hollow Spherical Covalent Organic Framework. *Nat. Commun.* **2015**, *6* (1), 6786.
(31) Gomes, R.; Bhanja, P.; Bhaumik, A. A Triazine-Based Covalent Organic Polymer for Efficient CO_2 Adsorption. *Chem. Commun.* **2015**, *51* (49), 10050–10053.
(32) Zhao, Y.; Liu, H.; Wu, C.; Zhang, Z.; Pan, Q.; Hu, F.; Wang, R.; Li, P.; Huang, X.; Li, Z. Fully Conjugated Two-Dimensional Sp^2-Carbon Covalent Organic Frameworks as Artificial Photosystem I with High Efficiency. *Angew. Chem. Int. Ed.* **2019**, *58* (16), 5376–5381.
(33) Wang, S.; Ma, L.; Wang, Q.; Shao, P.; Ma, D.; Yuan, S.; Lei, P.; Li, P.; Feng, X.; Wang, B. Covalent Organic Frameworks: A Platform for the Experimental Establishment of the Influence of Intermolecular Distance on Phosphorescence. *J. Mater. Chem. C* **2018**, *6* (20), 5369–5374.
(34) Das, G.; Biswal, B. P.; Kandambeth, S.; Venkatesh, V.; Kaur, G.; Addicoat, M.; Heine, T.; Verma, S.; Banerjee, R. Chemical Sensing in Two Dimensional Porous Covalent Organic Nanosheets. *Chem. Sci.* **2015**, *6* (7), 3931–3939.
(35) Li, Z.-J.; Ding, S.-Y.; Xue, H.-D.; Cao, W.; Wang, W. Synthesis of –C=N– Linked Covalent Organic Frameworks via the Direct Condensation of Acetals and Amines. *Chem. Commun.* **2011**, *52* (45), 7217–7220.
(36) Chen, R.; Hu, T.; Li, Y. Stable Nitrogen-Containing Covalent Organic Framework as Porous Adsorbent for Effective Iodine Capture from Water. *React. Funct. Polym.* **2021**, *159*, 104806.
(37) Lin, Z.; Dai, S.; Yao, S.; Lin, Q.-C.; Fu, M.; Chung, L.-H.; Han, B.; He, J. Diacetylene-Bridged Covalent Organic Framework as Crystalline Graphdiyne Analogue for Photocatalytic Hydrogen Evolution. *Chem. Sci.* **2025**, *16* (4), 1948–1956.
(38) Spitler, E. L.; Dichtel, W. R. Lewis Acid-Catalysed Formation of Two-Dimensional Phthalocyanine Covalent Organic Frameworks. *Nat. Chem.* **2010**, *2* (8), 672–677.
(39) Liao, H.; Wang, H.; Ding, H.; Meng, X.; Xu, H.; Wang, B.; Ai, X.; Wang, C. A 2D Porous Porphyrin-Based Covalent Organic Framework for Sulfur Storage in Lithium–Sulfur Batteries. *J. Mater. Chem. A* **2016**, *4* (19), 7416–7421.
(40) Wan, S.; Gándara, F.; Asano, A.; Furukawa, H.; Saeki, A.; Dey, S. K.; Liao, L.; Ambrogio, M. W.; Botros, Y. Y.; Duan, X.; Seki, S.; Stoddart, J. F.; Yaghi, O. M. Covalent Organic Frameworks with High Charge Carrier Mobility. *Chem. Mater.* **2011**, *23* (18), 4094–4097.
(41) Zhao, S.; Dong, B.; Ge, R.; Wang, C.; Song, X.; Ma, W.; Wang, Y.; Hao, C.; Guo, X.; Gao, Y. Channel-Wall Functionalization in Covalent Organic Frameworks for

the Enhancement of CO_2 Uptake and CO_2/N_2 Selectivity. *RSC Adv.* **2016**, *6* (45), 38774–38781.
(42) Chen, R.; Shi, J.; Ma, Y.; Lin, G.; Lang, X.; Wang, C. Designed Synthesis of a 2D Porphyrin-Based Sp^2 Carbon-Conjugated Covalent Organic Framework for Heterogeneous Photocatalysis. *Angew. Chem. Int. Ed.* **2019**, *58* (19), 6430–6434.
(43) Jin, S.; Sakurai, T.; Kowalczyk, T.; Dalapati, S.; Xu, F.; Wei, H.; Chen, X.; Gao, J.; Seki, S.; Irle, S.; Jiang, D. Two-Dimensional Tetrathiafulvalene Covalent Organic Frameworks: Towards Latticed Conductive Organic Salts. *Chem. Eur. J.* **2014**, *20* (45), 14608–14613.
(44) Feng, S.; Xu, H.; Zhang, C.; Chen, Y.; Zeng, J.; Jiang, D.; Jiang, J.-X. Bicarbazole-Based Redox-Active Covalent Organic Frameworks for Ultrahigh-Performance Energy Storage. *Chem. Commun.* **2017**, *53* (82), 11334–11337.
(45) Dalapati, S.; Jin, S.; Gao, J.; Xu, Y.; Nagai, A.; Jiang, D. An Azine-Linked Covalent Organic Framework. *J. Am. Chem. Soc.* **2013**, *135* (46), 17310–17313.
(46) Rabbani, M. G.; Sekizkardes, A. K.; Kahveci, Z.; Reich, T. E.; Ding, R.; El-Kaderi, H. M. A 2D Mesoporous Imine-Linked Covalent Organic Framework for High Pressure Gas Storage Applications. *Chem. Eur. J.* **2013**, *19* (10), 3324–3328.
(47) Dong, B.; Wang, L.; Zhao, S.; Ge, R.; Song, X.; Wang, Y.; Gao, Y. Immobilization of Ionic Liquids to Covalent Organic Frameworks for Catalyzing the Formylation of Amines with CO_2 and Phenylsilane. *Chem. Commun.* **2016**, *52* (44), 7082–7085.
(48) Auras, F.; Ascherl, L.; Hakimioun, A. H.; Margraf, J. T.; Hanusch, F. C.; Reuter, S.; Bessinger, D.; Döblinger, M.; Hettstedt, C.; Karaghiosoff, K.; Herbert, S.; Knochel, P.; Clark, T.; Bein, T. Synchronized Offset Stacking: A Concept for Growing Large-Domain and Highly Crystalline 2D Covalent Organic Frameworks. *J. Am. Chem. Soc.* **2016**, *138* (51), 16703–16710.
(49) Jin, E.; Li, J.; Geng, K.; Jiang, Q.; Xu, H.; Xu, Q.; Jiang, D. Designed Synthesis of Stable Light-Emitting Two-Dimensional Sp^2 Carbon-Conjugated Covalent Organic Frameworks. *Nat. Commun.* **2018**, *9* (1), 4143.
(50) Ding, H.; Li, Y.; Hu, H.; Sun, Y.; Wang, J.; Wang, C.; Wang, C.; Zhang, G.; Wang, B.; Xu, W.; Zhang, D. A Tetrathiafulvalene-Based Electroactive Covalent Organic Framework. *Chem. Eur. J.* **2014**, *20* (45), 14614–14618.
(51) Tian, Y.; Xu, S.-Q.; Liang, R.-R.; Qian, C.; Jiang, G.-F.; Zhao, X. Construction of Two Heteropore Covalent Organic Frameworks with Kagome Lattices. *CrystEngComm* **2020**, *19* (33), 4877–4881.
(52) Dalapati, S.; Jin, E.; Addicoat, M.; Heine, T.; Jiang, D. Highly Emissive Covalent Organic Frameworks. *J. Am. Chem. Soc.* **2016**, *138* (18), 5797–5800.
(53) Zhou, T.-Y.; Xu, S.-Q.; Wen, Q.; Pang, Z.-F.; Zhao, X. One-Step Construction of Two Different Kinds of Pores in a 2D Covalent Organic Framework. *J. Am. Chem. Soc.* **2014**, *136* (45), 15885–15888.
(54) Dong, J.; Li, X.; Peh, S. B.; Yuan, Y. D.; Wang, Y.; Ji, D.; Peng, S.; Liu, G.; Ying, S.; Yuan, D.; Jiang, J.; Ramakrishna, S.; Zhao, D. Restriction of Molecular Rotors in Ultrathin Two-Dimensional Covalent Organic Framework Nanosheets for Sensing Signal Amplification. *Chem. Mater.* **2019**, *31* (1), 146–160.
(55) Bai, L.; Gao, Q.; Zhao, Y. Two Fully Conjugated Covalent Organic Frameworks

as Anode Materials for Lithium Ion Batteries. *J. Mater. Chem. A* **2016**, *4* (37), 14106–14110.
(56) Rodríguez-San-Miguel, D.; Yazdi, A.; Guillerm, V.; Pérez-Carvajal, J.; Puntes, V.; Maspoch, D.; Zamora, F. Confining Functional Nanoparticles into Colloidal Imine-Based COF Spheres by a Sequential Encapsulation–Crystallization Method. *Chem. Eur. J.* **2017**, *23* (36), 8623–8627.
(57) Miller, K. A.; Alemany, L. B.; Roy, S.; Yan, Q.; Demingos, P. G.; Singh, C. V.; Alahakoon, S.; Egap, E.; Thomas, E. L.; Ajayan, P. M. High-Strength, Microporous, Two-Dimensional Polymer Thin Films with Rigid Benzoxazole Linkage. *ACS Appl. Mater. Interfaces* **2022**, *14* (1), 1861–1873.
(58) Rizzo, D. J.; Dai, Q.; Bronner, C.; Veber, G.; Smith, B. J.; Matsumoto, M.; Thomas, S.; Nguyen, G. D.; Forrester, P. R.; Zhao, W.; Jørgensen, J. H.; Dichtel, W. R.; Fischer, F. R.; Li, H.; Bredas, J.-L.; Crommie, M. F. Revealing the Local Electronic Structure of a Single-Layer Covalent Organic Framework through Electronic Decoupling. *Nano Lett.* **2020**, *20* (2), 963–970.
(59) Wang, Y.; Li, X.; Dong, X.; Zhang, F.; Jiang, D.; Sun, Y.; Wang, R.; Lang, X. Tuning the Photocatalytic Activity through Linkages of Porous Conjugated Polymers Based on Triazines for a Selective Oxidation Reaction. *Adv. Energy Sustain. Res.* **2023**, *4* (1), 2200129.
(60) Zha, Z.; Xu, L.; Wang, Z.; Li, X.; Pan, Q.; Hu, P.; Lei, S. 3D Graphene Functionalized by Covalent Organic Framework Thin Film as Capacitive Electrode in Alkaline Media. *ACS Appl. Mater. Interfaces* **2015**, *7* (32), 17837–17843.
(61) Aiyappa, H. B.; Thote, J.; Shinde, D. B.; Banerjee, R.; Kurungot, S. Cobalt-Modified Covalent Organic Framework as a Robust Water Oxidation Electrocatalyst. *Chem. Mater.* **2016**, *28* (12), 4375–4379.
(62) Pyles, D. A.; Coldren, W. H.; Eder, G. M.; Hadad, C. M.; McGrier, P. L. Mechanistic Investigations into the Cyclization and Crystallization of Benzobisoxazole-Linked Two-Dimensional Covalent Organic Frameworks. *Chem. Sci.* **2018**, *9* (30), 6417–6423.
(63) Fang, Q.; Sui, C.; Wang, C.; Zhai, T.; Zhang, J.; Liang, J.; Guo, H.; Sandoz-Rosado, E.; Lou, J. Strong and Flaw-Insensitive Two-Dimensional Covalent Organic Frameworks. *Matter* **2021**, *4* (3), 1017–1028.
(64) Jhulki, S.; Evans, A. M.; Hao, X.-L.; Cooper, M. W.; Feriante, C. H.; Leisen, J.; Li, H.; Lam, D.; Hersam, M. C.; Barlow, S.; Brédas, J.-L.; Dichtel, W. R.; Marder, S. R. Humidity Sensing through Reversible Isomerization of a Covalent Organic Framework. *J. Am. Chem. Soc.* **2020**, *142* (2), 783–791.
(65) Hou, Y.; Zhang, X.; Sun, J.; Lin, S.; Qi, D.; Hong, R.; Li, D.; Xiao, X.; Jiang, J. Good Suzuki-Coupling Reaction Performance of Pd Immobilized at the Metal-Free Porphyrin-Based Covalent Organic Framework. *Microporous Mesoporous Mater.* **2015**, *214*, 108–114.
(66) Gao, Q.; Li, X.; Ning, G.-H.; Leng, K.; Tian, B.; Liu, C.; Tang, W.; Xu, H.-S.; Loh, K. P. Highly Photoluminescent Two-Dimensional Imine-Based Covalent Organic Frameworks for Chemical Sensing. *Chem. Commun.* **2018**, *54* (19), 2349–2352.

(67) Cui, F.-Z.; Xie, J.-J.; Jiang, S.-Y.; Gan, S.-X.; Ma, D.-L.; Liang, R.-R.; Jiang, G.-F.; Zhao, X. A Gaseous Hydrogen Chloride Chemosensor Based on a 2D Covalent Organic Framework. *Chem. Commun.* **2019**, *55* (31), 4550–4553.
(68) Li, H.; Brédas, J.-L. Impact of Structural Defects on the Elastic Properties of Two-Dimensional Covalent Organic Frameworks (2D COFs) under Tensile Stress. *Chem. Mater.* **2021**, *33* (12), 4529–4540.
(69) Wang, B.; Ying, P.; Zhang, J. The Thermoelastic Properties of Monolayer Covalent Organic Frameworks Studied by Machine-Learning Molecular Dynamics. *Nanoscale* **2023**, *16* (1), 237–248.
(70) Giri, A.; Evans, A. M.; Rahman, M. A.; McGaughey, A. J. H.; Hopkins, P. E. Highly Negative Poisson's Ratio in Thermally Conductive Covalent Organic Frameworks. *ACS Nano* **2022**, *16* (2), 2843–2851.
(71) Suárez-Villagrán, M. Y.; Botari, T.; Miller, J. H.; Machado, L. D. Prediction of Strain-Controlled Adhesion in a Single-Layer Covalent Organic Framework. *Carbon* **2019**, *143*, 172–178.
(72) Furukawa, H.; Yaghi, O. M. Storage of Hydrogen, Methane, and Carbon Dioxide in Highly Porous Covalent Organic Frameworks for Clean Energy Applications. *J. Am. Chem. Soc.* **2009**, *131* (25), 8875–8883.
(73) Hao, Q.; Zhao, C.; Sun, B.; Lu, C.; Liu, J.; Liu, M.; Wan, L.-J.; Wang, D. Confined Synthesis of Two-Dimensional Covalent Organic Framework Thin Films within Superspreading Water Layer. *J. Am. Chem. Soc.* **2018**, *140* (38), 12152–12158.
(74) Martín-Illán, J. Á.; Suárez, J. A.; Gómez-Herrero, J.; Ares, P.; Gallego-Fuente, D.; Cheng, Y.; Zhao, D.; Maspoch, D.; Zamora, F. Ultralarge Free-Standing Imine-Based Covalent Organic Framework Membranes Fabricated via Compression. *Adv. Sci.* **2022**, *9* (7), 2104643.
(75) Zhu, D.; Hu, Z.; Rogers, T. K.; Barnes, M.; Tseng, C.-P.; Mei, H.; Sassi, L. M.; Zhang, Z.; Rahman, M. M.; Ajayan, P. M.; Verduzco, R. Patterning, Transfer, and Tensile Testing of Covalent Organic Framework Films with Nanoscale Thickness. *Chem. Mater.* **2021**, *33* (17), 6724–6730.
(76) Chen, D.; Huang, S.; Zhong, L.; Wang, S.; Xiao, M.; Han, D.; Meng, Y. In Situ Preparation of Thin and Rigid COF Film on Li Anode as Artificial Solid Electrolyte Interphase Layer Resisting Li Dendrite Puncture. *Adv. Funct. Mater.* **2020**, *30* (7), 1907717.
(77) Fang, Q.; Pang, Z.; Ai, Q.; Liu, Y.; Zhai, T.; Steinbach, D.; Gao, G.; Zhu, Y.; Li, T.; Lou, J. Superior Mechanical Properties of Multilayer Covalent-Organic Frameworks Enabled by Rationally Tuning Molecular Interlayer Interactions. *Proc. Natl. Acad. Sci.* **2023**, *120* (15), e2208676120.
(78) Yang, Y.; Liang, B.; Kreie, J.; Hambsch, M.; Liang, Z.; Wang, C.; Huang, S.; Dong, X.; Gong, L.; Liang, C.; Lou, D.; Zhou, Z.; Lu, J.; Yang, Y.; Zhuang, X.; Qi, H.; Kaiser, U.; Mannsfeld, S. C. B.; Liu, W.; Gölzhäuser, A.; Zheng, Z. Elastic Films of Single-Crystal Two-Dimensional Covalent Organic Frameworks. *Nature* **2024**, *630* (8018), 878–883.
(79) Zhang, J. Phase Transformation in Two-Dimensional Covalent Organic Frameworks under Compressive Loading. *Phys. Chem. Chem. Phys.* **2018**, *20* (46),

29462–29471.
(80) Oliveira, F. L.; Esteves, P. M. pyCOFBuilder: A Python Package for Automated Creation of Covalent Organic Framework Models Based on the Reticular Approach. *J. Chem. Inf. Model.* **2024**, *64* (8), 3278–3289.
